# MGS-Fast: Metagenomic shotgun data fast annotation using microbial gene catalogs

**DOI:** 10.1093/gigascience/giz020

**Published:** 2019-04-03

**Authors:** Stuart M Brown, Hao Chen, Yuhan Hao, Bobby P Laungani, Thahmina A Ali, Changsu Dong, Carlos Lijeron, Baekdoo Kim, Claudia Wultsch, Zhiheng Pei, Konstantinos Krampis

**Affiliations:** 1 New York University Langonne Medical Center, 333 E 38th St, New York, NY, 10016, USA; 2 Department of Biological Sciences and Center for Translational and Basic Research, Belfer Research Building, Hunter College of The City University of New York, 333 E 38th St, New York, NY, 10016, US; 3 Research Foundation of The City University of New York, 333 E 38th St, New York, NY, 10016, USA; 4 Sackler Institute for Comparative Genomics, American Museum of Natural History, 333 E 38th St, New York, NY, 10016, USA; 5 Department of Veterans Affairs New York Harbor Healthcare System, 333 E 38th St, New York, NY, 10016, USA; 6 Institute of Computational Biomedicine, Weill Cornell Medical College, 333 E 38th St, New York, NY, 10016, USA

**Keywords:** metagenomics, annotation, cloud computing, Docker, Galaxy

## Abstract

**Background:**

Current methods used for annotating metagenomics shotgun sequencing (MGS) data rely on a computationally intensive and low-stringency approach of mapping each read to a generic database of proteins or reference microbial genomes.

**Results:**

We developed MGS-Fast, an analysis approach for shotgun whole-genome metagenomic data utilizing Bowtie2 DNA-DNA alignment of reads that is an alternative to using the integrated catalog of reference genes database of well-annotated genes compiled from human microbiome data. This method is rapid and provides high-stringency matches (>90% DNA sequence identity) of the metagenomics reads to genes with annotated functions. We demonstrate the use of this method with data from a study of liver disease and synthetic reads, and Human Microbiome Project shotgun data, to detect differentially abundant Kyoto Encyclopedia of Genes and Genomes gene functions in these experiments. This rapid annotation method is freely available as a Galaxy workflow within a Docker image.

**Conclusions:**

MGS-Fast can confidently transfer functional annotations from gene databases to metagenomic reads, with speed and accuracy.

## Background

The initial focus of metagenomics studies, such as the Human Microbiome Project (HMP) [1], was to survey the microbial communities present in various sites on and in the human body, but the focus of research has now shifted to understanding the functional role these microbes play in metabolic and disease processes. Assessment of the taxonomic diversity and composition of metagenome samples using amplicon sequencing of the 16S ribosomal RNA marker gene is inexpensive and has been applied to map a wide variety of microbial communities, but it is also subject to bias and lacks sensitivity below the species level. It is known that individual bacterial isolates with identical 16S genes may differ by as much as 15–30% in their genomes [2], which may include genes with toxin production, antimicrobial, or metabolic functions. Alternatively, metagenomics shotgun sequencing (MGS) of all DNA present in a biological sample can be used for computational prediction of gene functions of sequenced DNA fragments to infer differences in the biological function of microbial communities [3]. Existing bioinformatics tools to characterize MGS data face bottlenecks owing to the large computational task of comparing millions of short DNA sequences (50–200 nucleotides in length) to various databases of known proteins, conserved protein motifs, or annotated genomes. The BLAST [4] method is used to compare DNA sequences (i.e., reads) to a database, requiring hundreds of CPU hours to analyze a typical MGS sample containing hundreds of millions of reads.

Approaches to overcome this computational bottleneck include the reduction of read data file complexity, e.g., through deduplication or by *de novo* assembly. However, these data reduction methods themselves require substantial computational effort and can introduce significant bias. Furthermore, misassemblies can introduce significant biases because the whole-genome sequencing (WGS) reads correspond to hundreds of bacterial genomes and chimeric contigs can be created [5]. This is especially true for gut microbiomes, where closely related species with similar genomes are present, and this could be exacerbated in the case where significant gene transfer occurs across species (transposase, phage, and lateral gene transfer). The result of a misassembly is to distort abundance information because genomic sequences could be assembled together, resulting in losing the signal for species present in the sample. In addition, one of the advantages of the mapping approach is that it is possible to recover the presence of a gene even when the coverage for that gene is not sufficient for assembling it. Therefore, gene presence in the sample can be better identified using the raw reads and comparing them with annotation databases, rather than assemblies that make it difficult to ensure that species are not artificially masked during the assembly process.

Other methods involve the use of faster but less sensitive sequence-matching algorithms such as BLAT [6] or RAPSearch (MG-RAST webserver [7]), or reduced databases for functional protein identification, thus providing a less precise assay for microbial protein function. However, with the MG-RAST webserver, the wait queue for data processing can be up to several weeks. Carr and Borenstein [8] compared MGS annotation using BLAST with using BWA (a DNA sequence similarity tool very similar to Bowtie), and they conclude that at short evolutionary distances, BWA has a higher precision and recall than BLAST for identifying Kyoto Encyclopedia of Genes and Genomes (KEGG) orthologs, but recall and precision for BWA drops dramatically at greater evolutionary distances.

## Results

### MGS-Fast algorithm and software implementation

**Figure 1:**
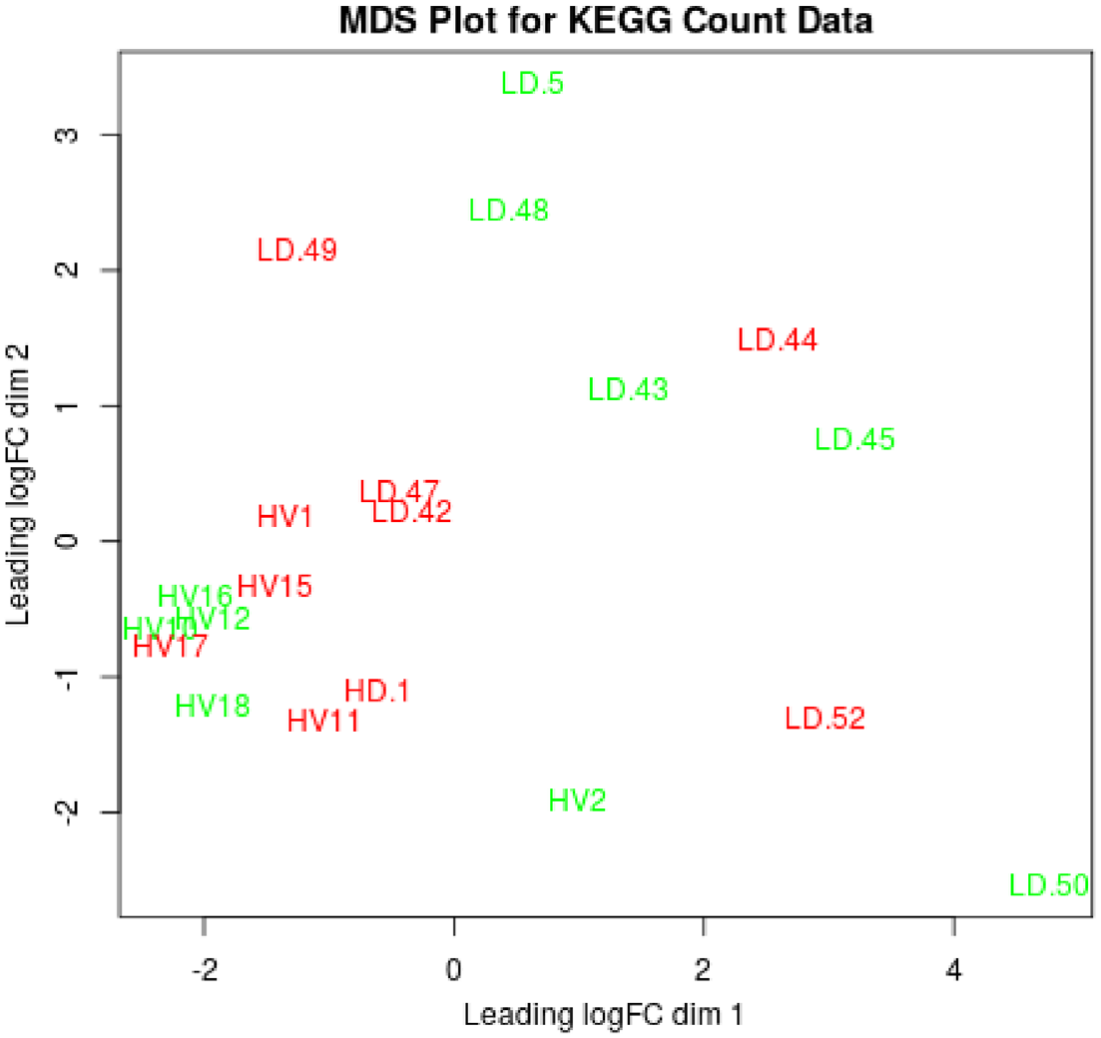
Multidimensional scaling (MDS) abundance plot generated using the R package *edgeR* and a mixture model with a negative binomial distribution, for the KEGG annotations generated by MGS-Fast using as input data from gut microbiomes of healthy patients (HV) and patients with liver cirrhosis (LD).

We created a computationally efficient pipeline for MGS data analysis called MGS-Fast, which combines several data preprocessing steps (read data trimming and filtering of low-quality sequences, removal of human host contaminant sequences) with taxonomic and functional profiling of metagenomic WGS data. The pipeline leverages software broadly used in the bioinformatics community for quality control, taxonomy, DNA sequence alignment, and taxonomic profiling (details in Methods section). The novelty of MGS-Fast lies in the use of stringent DNA-DNA matching to annotated and high-quality bacterial DNA sequences from the integrated reference catalog of the human gut microbiome (IGC) [9]. The IGC database contains 9 ,879 ,896 gut microbe genes with annotations based on the Kyoto Encyclopedia of Genes and Genomes (KEGG; [10, 11]). The bioinformatics workflow of MGS-Fast utilizes Bowtie 2 to rapidly map a MGS data set and assign known functions to reads originating from microbial genes, producing counts of KEGG gene orthologs as output. The KEGG counts are then applied to identify differentially abundant microbial gene functions in metagenomics data sets, and separate samples from different patient groups (Fig. 1). The IGC database is precompiled as a Bowtie 2 index that is deployed automatically during installation of MGS-Fast and is also available as a separate download (Availability section). Furthermore, the MGS-Fast pipeline is packaged as preconfigured, ready-to-execute software within a Docker container, which is easy to deploy without bioinformatics expertise through a single command (Supplementary Information: Software Manual). Researchers working with nonhuman MGS gut data can create their own custom database of microbial genes (Methods section) for functional profiling. Once a custumized database is prepared, MGS-Fast allows for parallel processing of multiple WGS metagenomic samples with increased accuracy and reduced computational time for the functional assignment of reads.

### Data analysis of liver cirrhosis metagenomic samples

In our study, the MGS-Fast pipeline was used for the analysis of gut microbiome samples from 10 patients with liver cirrhosis and 10 control samples from an earlier study by Qin et al. [12], obtained from the European Nucleotide Archive, accession ERP005860. Upon completion, the pipeline generated gene function abundance counts, with a total of 3,785 KEGG IDs, which was similar to the number (4,801) in the original study by Qin et al. Next, following the recommendations of McMurdie and Holmes [13], we analyzed 502 of 3,785 KEGG IDs that had significantly different abundance scores (false discovery rate–corrected *P*-value threshold 0.05; Suppl. KEGG-FDR.CSV) as a mixture model with a negative binomial distribution, using the R package *edgeR*, version 3.7 [14]. To visualize the differences between groups of KEGG ID abundances, we also used *edgeR* to create a multidimensional scaling plot (Fig. 1), in which clear separation was observed between the healthy and cirrhosis samples.

Next, we mapped 502 false discovery rate–corrected (0.05) KEGG IDs returned by MGS-Fast to pathways using the KEGG website tools [15]. We were able to identify functional groups and pathways modules that corresponded closely to the ones found in the original study by Qin et al. [12]. Specifically, the majority of the pathway modules detected were for membrane transport, including oligopeptide transport systems, in addition to zinc, glutamine, and energy coupling factor transport (complete list in Suppl. file KEGG modules–502 KEGG IDs.doc). Furthermore, and as reported in the original study, we found prevalent pathway modules for carbohydrate, amino acid, and energy metabolism, including the citrate, Krebs, and Calvin cycles, gluconeogenesis, and glyoxylate and glycolysis cycles. We also observed a set of liver cirrhosis–associated markers similarly to Qin et al., including assimilatory nitrate reduction, denitrification, γ-aminobutyric acid (GABA) biosynthesis, and GABA shunt, in addition to heme biosynthesis. The GABA neurotransmitter system is correlated with brain disease [16] as a result of liver dysfunction, because of increased GABA levels in the blood have the potential to penetrate the blood–brain barrier and cause hepatic encephalopathy. Finally, we also detected a set of pathway modules for ammonia production, which could lead to increases ammonia levels in the blood as described by the original study (Qin et al. 2014 [12]). In this respect, we also found the assimilatory nitrate reduction pathway module to be present, in addition to dissimilatory nitrate reduction and the complete nitrification pathway (Suppl. file KEGG modules–502 KEGG IDs.doc).

### Comparative pipeline performance and processing times

The MGS-Fast Docker container was deployed on an 8-CPU Intel Xeon Server supporting hyper-threading for a total of 8 parallel processes ("threads"), in addition to 128 GB RAM memory. This is a high-performance computing server, commonly found in laboratories performing genome sequencing bioinformatics. To compare the computational performance of MGS-Fast with that of other published pipelines for metagenomic annotation, we measured the processing time for each tool in the different pipelines using the patient data sets from our study. To ensure the compatibility of the results, we applied the option "–threads 8" or similar for all pipelines (Kraken, GOTTCHA [17, 18], and HumanAn2 [19]) included in our comparison. The pipelines were set up according to the documentation for each, using the standard full database for Kraken (kraken-build –standard –db $DBNAME) and the latest bacterial databases for GOTTCHA [20]. Processing times for the MGS-Fast workflow (sample ID ERR526291, number of reads 15,181, 542 × 2) in comparison with the other pipelines are presented in Table 1.

**Table 1:** Processing times for the MGS-Fast pipeline in comparison with other workflows (Kraken, HumanAn2, GOTTCHA) used for WGS metagenomics analysis

Workflow	Data set ID: No. of reads × paired	Data quality control (FASTQC) (min)	Data preparation (Groomer) (min)	Trimmomatic (min)	Human filtering (Bowtie) (min)	Taxonomic classification (MetaPhlAn) (min)	Annotation (Bowtie IGC) (min)	KEGG count (min)	Total (without filtering) (min)
MGS-Fast	ERR526291: 15,181,542 × 2	2	6	2	6	23	22	4	49 (16)
Kraken		N/A	N/A	N/A	N/A	254			254
HumanAn2		N/A	N/A	N/A	N/A	162			162
GOTTCHA		N/A	N/A	(1*)	N/A	17			17

Time for data preprocessing steps (quality control of metagenomic data, filtering of host DNA) performed by MGS-Fast is listed in parentheses. Time is with 8× threads. N/A: not applicable. (1*) The only tool that has a built-in trimming option.

**Figure 2:**
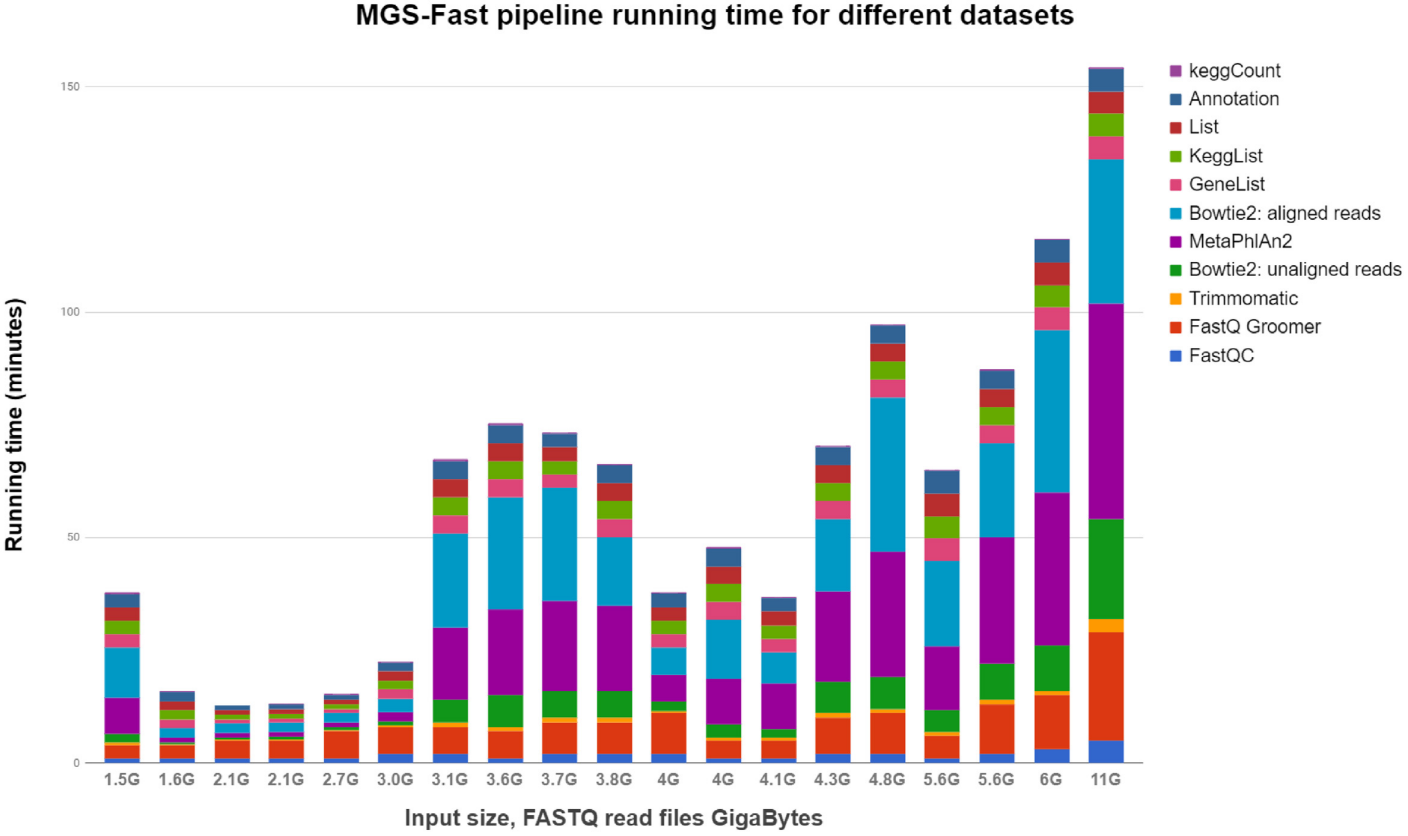
Processing times for all MGS-Fast pipeline steps when used for analysis of different patient metagenomic data sets ranging from 1.5 to 11 GB in size.

**Figure 3:**
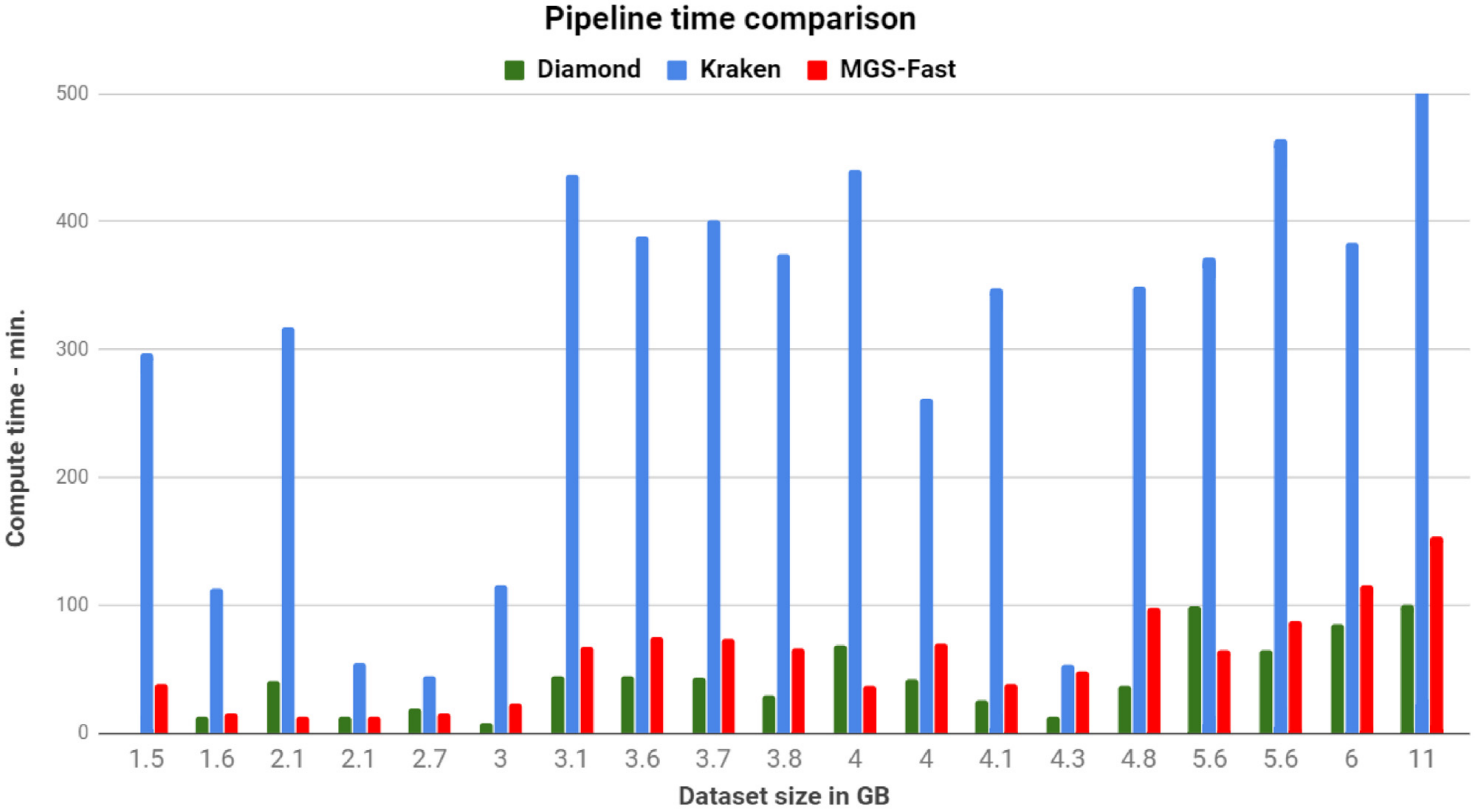
Comparison of running time for MGS-Fast, Kraken, and DIAMOND, using the same database built by the IGC sequence set for each tool. All input samples, compiled database, and outputs can be found at http://146.95.173.35:9988/MGS-FAST/.

Interestingly, most other pipelines besides MGS-Fast do not perform preprocessing of data such as quality control or removal of host WGS reads, except for GOTTCHA, which offers users the option to trim input DNA reads. Table 1 lists the times required by MGS-Fast for the data preprocessing steps, including read trimming and removal of host sequences. Furthermore, Fig. 2 reports processing times for all MGS-Fast pipeline steps when used for analysis of different patient metagenomic data sets, which ranged from 1.5 to 11 GB in size. Similarly, Fig. 3 compares total time of execution for the same data sets with Kraken, which uses a large in-memory k-mer database, versus the compressed Burrows-Wheeler transform (BWT)—Bowtie2 aligner used for MGS-Fast, and DIAMOND using protein-based alignment.

Our comparison data (Table 1) showed that MGS-Fast (49 min) was ∼3 times slower than GOTTCHA (17 min), while it was 4 and 5 times faster than HumanAn2 (162 min) and Kraken (254 min), respectively, for processing the ERR526291 data set (15,181, 542 × 2 paired reads). For HumanAn2, we observed that the software generates a bowtie index for the reads in the sample provided as input under a temporary files output directory ($OUTPUT_DIR/$SAMPLENAME_humann2_temp/, also described in its documentation), which might explain the additional time required in comparison to MGS-Fast. The significantly increased time required by Kraken to process the data set is not surprising because in the corresponding publication [17] it was reported that Kraken can process 150,000 reads per minute. With 30 million reads in the ERR526291 data set, it would require ≥200 minutes for the alignment, with additional time for the annotation, writing the output ,and other tasks. Furthermore, the computer server used for running all the software comparisons had ample RAM memory (128 GB), and examination of the Kraken output file revealed that no page faults were reported (this is a built-in feature of the software, where number memory faults minus disk swaps are reported), which, if present, would explain the increased time taken by Kraken. The database constructed by Kraken, using the "kraken-build" command that downloads reference data from the National Center for Biotechnology Information, was ∼164 GB in size. This is more than twice the 70 GB reported in the original 2015 publication,[16] and also reflects the fact that Kraken is a k-mer–based aligner and read sizes have increased (in our data set it was 100 base pairs × 2 paired end), resulting in more k-mers to be compared per read, necessitating increased time to process the sample.

### WGS metagenomic data sets used as controls for MGS-Fast

We further evaluated the performance of the MGS-Fast pipeline by using a range of metagenomic samples from mouse and human collected across different body sites (e.g., gut, mouth, skin), in addition to environmental samples (e.g., copper mine waste), and negative controls of simulated read data from real or synthetic genomes (Table 2). The Human Oral Microbiome Database (HOMD; [21]) was included in the analysis workflow as additional annotation data. In more detail, Bowtie 2 alignments of human gut (fecal) samples performed by MGS-Fast resulted in 95.62% of all reads in the sample being successfully mapped to the database. For human oral and skin microbiome samples 82.32% and 33.02% of the reads, respectively (Table 2), were mapped to the database.

**Table 2:** Bowtie 2 alignment of different metagenomic samples to our IGC/HOMD derivative database

Metagenome	Sample accession/source	Alignment to database (%)
Human gut	SRR2822459	95.62
Human gut, liver disease	ENA ERP005860	96.03
Mouse gut	MG-RAST 4535626.3	89.71
Human mouth	SRS016533	82.32
Human esophagus	SRS065335	71.39
Human vagina	SRS014465	66.39
Human skin	SRR1646957	33.02
Human genome GRCh38	MetaSim simulated	7.35 (false positives)
*E. coli* K12 genome	MetaSim simulated	98.50
HMP Mock	SRR172902	28.82
Synthetic microbial reads	SRR3732372	10.23
Copper mine waste	MG-RAST 4664533.3	8.69
Randomly generated reads	XS simulator	0.53

Simulated FASTQ reads from the human reference genome GRCh38 aligned at only 7.35%, which was expected because our pipeline filters out human sequences. As a positive control, we mapped MetaSim [22] simulated reads from the *Escherichia coli* K12 reference genome (GenBank: accession U00096.3) and 98.5% of the sample was aligned. Furthermore, as negative controls we included the HMP mock microbial community (SRR172902; 28.82% of reads aligned to database), a synthetic metagenome (SRR3732372) made from a mixture of DNA from laboratory strains of bacteria (10.23% of reads aligned to database), and a copper mine waste sample (MG-RAST accession 4664533.3; 8.69% of reads aligned to database; Table 2). Finally, false-positive matches were evaluated by aligning a set of randomly generated reads by means of the XS simulator [23]. As expected, only 0.53% of the sample reads aligned to our database.

## Methods

### MGS-Fast pipeline structure and data processing

**Figure 4:**
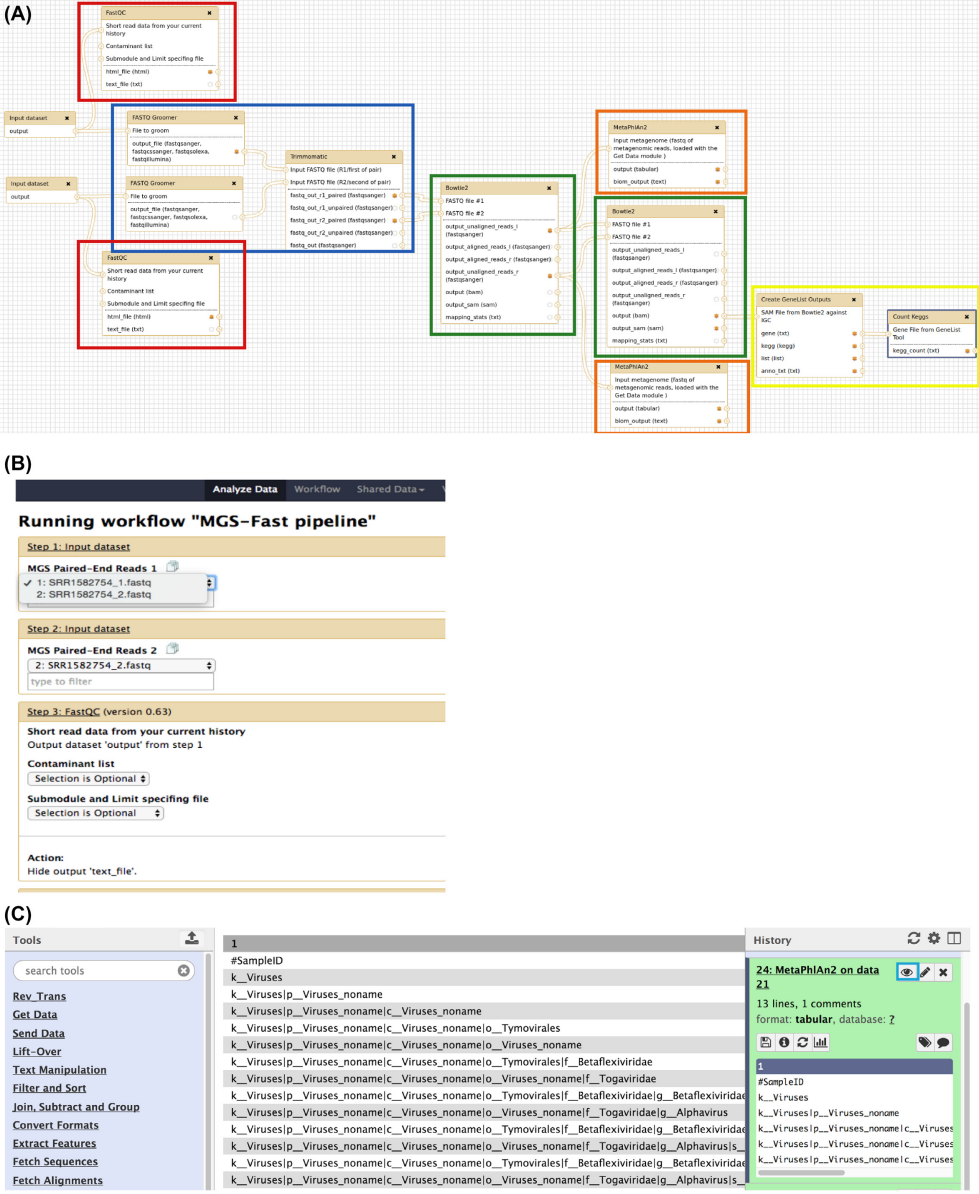
**(A)** MGS-Fast pipeline on the Galaxy workflow canvas, running on a Docker container. Read quality tools are outlined in red, score adjustment and trimming in blue, Bowtie 2 alignment to the IGC/HOMD or human reference in green, the MetaPhlAn analysis in orange, and annotation parsing from the Bowtie 2 results in yellow. **(B)** Interface of MGS-Fast pipeline on Galaxy web server running in the Docker container. Users can select the input data and parameters for the pipelines through dropdown menus and input boxes (details in Suppl. Software Manual). **(C)** The pipeline output for the MetaPhlAn tool, visualized within the Galaxy web interface.

The MGS-Fast workflow begins with quality control using FastQC (FastQC, RRID: SCR_014583) (red rectangles, Fig. 4A; [24]), which creates as output a report on many aspects of input data quality. Next, Trimmomatic (Trimmomatic, RRID: SCR_011848; [25], blue rectangle, Fig. 4A) is used to remove sequencing adapters, primers, and low-quality sequence data. Human host DNA is removed by alignment of reads to the human reference genome using Bowtie 2 (Bowtie, RRID: SCR_005476; [26], left green rectangle, Fig. 4A), using the human GRCh38 reference genome [27]. This filtering step retains only the "unmatched" reads corresponding to the metagenome as specified by the "–un" (unaligned) option from Bowtie 2. The retained reads are then aligned to the IGC microbiome gene catalog database with Bowtie 2 (right green rectangle, Fig. 4A), using the "–end-to-end –sensitive" option, in order to assign KEGG protein function IDs to each read. The IGC database [28] contains ∼10 million KEGG-annotated microbial genes, collected from 1,267 public human gut microbiome samples plus an additional 922 complete annotated prokaryotic genomes. The software versions included in this workflow are the following: FASTQC 0.11.6, Trimmomatic 0.32.1, Bowtie 2.2.6, and MetaPhlAn 2.5.0 (MetaPhlAn, RRID: SCR_004915) [29], with a default preset of parameters (details in **Supplementary Manual**) that can be easily adjusted and changed by the users through the Galaxy interface.

Next, a custom Python script integrated in the workflow (yellow rectangle, Fig. 4A) is used to count the number of IGC genes and KEGG IDs generated as output of Bowtie 2 with IGC. The script counts the number of reads aligned to each gene ID in the BAM file generated by Bowtie 2 and stores the counts in a 2-column "gene ID—abundances" file. The annotations were also parsed from the original IGC/HOMD FASTA files to produce a second file of "gene ID—KEGG ID" that was saved as a Python "dictionary" data structure. The gene ID—abundances are then read line by line using the Python script, which also loads the dictionary data structure and matches the gene ID—abundance list entries with these of the gene ID—KEGG ID based on the gene ID. The KEGG IDs of the matching lines from the 2 lists are then used by the script as a key for a new dictionary containing key-value pairs. The value corresponding to the KEGG ID keys of the dictionary is set to the corresponding abundance count; in addition, the count is incremented when the KEGG ID is already present in the dictionary as the lists are parsed. Following all data-processing steps, MGS-Fast prints the KEGG IDs and read counts for each gene in a text file, which is used as input file for the *EdgeR* script that creates abundance plots (“abundance-plot.R,” available at [30]). The R script filters out genes with low counts, keeping those rows where the count per million is ≥1 in ≥6 samples. The count per million for mapped reads is essentially counts scaled by the number of fragments sequenced in 1 million. Furthermore, we used the calcNormFactors function from *EdgeR* in our script, which normalizes for RNA composition by finding a set of scaling factors for the library sizes across samples. This essentially rescales the library size, resulting in an "effective" library size, which is then used for abundance calculations. This step helps to remove any further artifacts of read distributions per gene that might be introduced, e.g., at the initial stage of read trimming.

### MGS-Fast pipeline software distribution and data options

The MGS-Fast pipeline was developed using the workflow canvas of the Galaxy bioinformatics web server (Galaxy, RRID: SCR_006281; Fig. 4A, [31]), which was first pre-installed and configured to run within a Docker virtual machine container [32]. The Galaxy web server was chosen because it provides an intuitive web browser interface for nontechnical users. Users can easily access and run the MGS-Fast pipeline via the Galaxy interface, and developers can use the Galaxy workflow canvas to build and modify the pipeline.

Our goal was to develop a complete software bundle within a Docker container, which includes the MGS-Fast workflow and all required bioinformatics software, in addition to all other software dependencies. The entire pipeline is implemented in a series of steps (Fig. 4A), which are automated via Galaxy. The users only need to select the input data sets (Fig. 4B), and the Galaxy workflow engine will automatically execute all remaining analysis steps of the pipeline. Furthermore, the input data directory is attached and automatically available through the Galaxy interface when users set up MGS-Fast and specify the data directories (Suppl. Software Manual). Upon completion of an MGS-Fast pipeline run, the users can download all result files or simply view the output within the Galaxy interface (Fig. 4C). Furthermore, users can run MGS-Fast by reconfiguring the analysis steps or rerunning a single tool instead of the whole pipeline.

MGS-Fast also provides 2 options for users to create custom Bowtie 2 indexes both for filtering host genome reads and for annotating the metagenomic reads. Through the first option users can specify the location of a file containing the sequence of a host genome or metagenome, through a text-based menu during the initial run of the MGS-Fast container (Suppl. Manual). The scripts inside the container will automatically build an index for the provided genome and make it available for use on the Galaxy interface without any futher effort by the user. As a second option, we made an additional pipeline (Suppl. Material) available, which is called "Galaxy-Workflow-Custom_MGS-Fast.ga" and can be imported to an already installed and running MGS-Fast workflow. The custom workflow is identical to the regular workflow used by MGS-Fast but provides users the option to use a FASTA file containing the sequence(s) of the custom genome as input. The Bowtie 2 index for the provided genome is automatically built during the first run of the workflow and is then made available for all subsequent runs. Similarly, users can at any point add additional custom genome indexes, both for filtering host DNA reads and for classifying metagenomes. Users can download host genomes (e.g., mouse reference [33]) and also a range of WGS metagenomes from the Joint Genome Institute [34].

## Discussion

MGS-Fast can confidently transfer functional annotations from annotated gene databases to sequence reads in metagenomic data sets. For microbial read annotation and assignment of KEGG IDs by alignment to the International Genome Consortium, MGS-Fast uses the Bowtie 2 algorithm requiring by default 90% DNA sequence identity in finding matches. While the DNA to DNA alignment performed by Bowtie 2 is less sensitive than translated BLAST utilizing information from conservative amino acid substitutions, at the 90% level of identity, we only have exact matches from DNA fragments of the same species, or orthologs between closely related species [35]. We have also considered increasing the sensitivity of our method by changing the Bowtie 2 parameters and including the "–very-sensitive-local" parameter or increasing the number of allowed mismatches but decided against it because it would allow less stringent DNA-DNA alignments and more false-positive results. Nonetheless, this option is still available for users because the Bowtie 2 parameters can be easily adjusted through the Galaxy interface when a MGS-Fast pipeline run is initiated. However, caution is required in interpreting the results with increased sensitivity parameters because a high percentage of false-positive alignments will make assignment of metabolic function to metagenomic reads less reliable. In addition to Bowtie 2 our pipeline also provides annotations through MetaPhlAn2, using a database of ∼1 million unique clade-specific marker genes from 17,000 reference genomes. This enables MGS-Fast to identify taxa within narrow clades, even in the absence of reference genomes for species in the gut community. MetaPhlAn2 enabled us to identify a microbial organism at higher taxonomic levels of genera or family, and we observed identification of 8,931 organisms, of which 6,681 have been annotated by MetaPhlAn at the order taxonomic level.

Using Docker container technology, we bundled all required software components and the MGS-Fast pipeline as a preconfigured, ready-to use bioinformatics package for performing standardized, automated metagenomics analysis on any desktop or laptop computer running Windows, MacOS, or the Linux operating system. Both the container and source code are publicly available for download (Availability section) and can be easily installed by users without bioinformatics expertise with a single command (**Suppl**. Software Manual). Users can then simply access the MGS-Fast pipeline via the Galaxy interface by entering the network address of the container (made available to the user when the installation is complete) on their web browser. Furthermore, the Docker container can be deployed on the cloud or institutional clusters, where users can run multiple instances of MGS-Fast in parallel in order to process multiple NGS samples, or within a single instance of MGS-Fast using Galaxy's Data Collections input data options.

Regarding computational performance for large-scale studies, we tested MGS-Fast with a set of Illumina HiSeq 2000 oral microbiome data sets ranging from 1.5 to 11 GB (Fig. 2) in file size [36]. Using a computer server with average computational capacity (128 GB, 8-CPU core) the processing time for MGS-Fast ranged from 20 minutes for smaller read sets to 2.5 hours for the larger ones. The cumulative processing time to complete running MGS-Fast for all data sets included in this study was ∼15 hours (900 min). For large WGS metagenomic studies (100 samples), the complete study can be processed in the course of a few days. While running a single data set at a time on our computer server, we noticed that the hardware capacity was underutilized and decided to implement MGS-Fast analysis in parallel, reducing the total time required to process the data sets included here. In a production setting, where more computational capacity might also be available, researchers could use tens of instances at the same time and efficiently process large-scale data sets.

We then compared the performance of the compressed BWT–Bowtie 2 aligner used for MGS-Fast (Fig. 3) with Kraken [17] and DIAMOND [37], which use, respectively, a large in-memory k-mer database and protein-based alignment. The Kraken approach is based on a database of preclassified k-mers, and although it can classify millions of reads in just a few minutes, their memory requirements are usually high, requiring a high-performance, expensive computer server to which some laboratories do not have access in order to complete the analysis. In contrast, MGS-Fast is similar to multiple other published tools in the literature using the more efficient Bowtie 2 index structure, which allows for memory efficiency in storage and increased speed when querying the database [38], as also evidenced by our results in the present study.

Furthermore, our results are substantiated by the subsequent release by the authors of Kraken of a newer tool called Centrifuge [39], which, unlike Kraken and similar to other nucleotide-based classification tools in the literature, also uses the BWT for the genome database. This strategy uses one-tenth the space of a Kraken index for the same database, providing faster classification speed and lower memory requirements, making it possible to perform large-scale metagenomics annotation on a desktop computer. As reported in this study [39], Centrifuge took only 47 minutes on a standard desktop computer to analyze a total of 26 GB of input sequence data, and where Kraken and MegaBLAST required 100 and 25 GB of memory, respectively, for their indexes, Centrifuge requires only 4.2 GB. Similar results have been reported for multifold increased speed in comparison to Kraken with BWT use in PALADIN and Kaiju [40, 41], and as reported in these studies an important constraint for Kraken is its memory usage, where the database grows in linear proportion to the number of distinct k-mers in the genomic library (at 12 bytes per k-mer).

Regarding using nucleotide read alignment versus protein translated search for metagenomic classification, the fact is that amino acid sequences are conserved better at evolutionary distances, leading to more sensitive read classification in the case of distant species or taxa. Both DIAMOND and Kaiju [37, 41] align 6-frame translations of reads against a protein database. Similarly to what was mentioned above, Kaiju indexes the reference protein database using BWT as does our MGS-Fast tool, allowing metagenomic sequences to be searched quickly and with low memory footprint against a large protein database. Given a metagenomic sample and the prebuilt index, Kaiju first translates every read in all 6 reading frames, splitting the read at stop codons. As reported in this study, by using the BWT as an index for the reference protein database, Kaiju classifies up to millions of reads per minute and is typically faster than k-mer–based methods such Kraken and Clark [42].

## Availability of supporting data and materials

- GitHub repository with MGS-Fast code: https://github.com/BCIL/MGS-Fast
- Docker repository with the MGS-Fast container: https://hub.docker.com/r/bcil/metagenome/tags/(bcil/metagenome: nyu_4.0)
- IGC Indexes Database:http://bioitcore.hunter.cuny.edu:9988
- Human metagenomic reads, synthetic data, and *E. coli* data sets: http://bioitcore.hunter.cuny.edu:9988
- Testing data sets and precompiled genome indexes: http://bioitcore.hunter.cuny.edu:9988/Metagenomics_Package
- Furthermore, detailed instructions on the use of the Docker system and installation and use of the MGS-Fast image are available in the software manual as part of this manuscript.
- All software, indexes, and containers are released under open-source MIT license.

Snapshots of the code and sample data are also available in the *GigaScience* GigaDB repository [43].

## Additional files

V2 Suppl-Software-Manual.docx edger.r Suppl-KEGG-FDR.CSV KEGG modules-502 KEGG IDs.docx Galaxy-Workflow-Parallel_MGS-Fast.ga compute_times_compared.xlsx

## Abbreviations

BWT: Burrows-Wheeler transform; GABA: γ-aminobutyric acid; HMP: Human Microbiome Project; HOMD: Human Oral Microbiome Database; IGC: integrated reference catalog of the human gut microbiome; KEGG: Kyoto Encyclopedia of Genes and Genomes; MGS: metagenomics shotgun sequencing; PCR: polymerase chain reaction; WGS: whole-genome sequencing.

## Competing interests

The authors declare that they have no competing interests.

## Funding

Supported by CTBR NIMHD award G12 MD007599, WCMC-CTSC 2UL1TR000457, NYU Langone Medical Center, Assoc. of Chinese American Physicians, NCI, NIAID, NICDR awards UH3CA140233, U01CA182370, R01CA159036, R01AI110372, R21DE025352, U54CA22170401A16152.

## Author contributions

The construction and testing of the MGS-Fast method was implemented by S. Brown with assistance from Y. Hao and H. Chen. The Docker image for MGS-Fast was built by B. Laungani with assistance from T. Ali, C. Dong, C. Lijeron, and B. Kim. The data analysis was performed and the manuscript text was written by K. Krampis, S. Brown and C. Wultsch. The supplemental software manual was written jointly by B. Laungani with additions by K. Krampis, T. Ali, C. Dong, C. Lijeron, B. Kim and C. Wultsch.

## Author information

The content is the sole responsibility of the authors and does not represent the views of the National Institutes of Health, the U.S. Department of Veterans Affairs, or the U.S. Government.

## Supplementary Material

GIGA-D-18-00255_Original-Submission.pdf

GIGA-D-18-00255_Revision-1.pdf

Response-to-Reviewer-Comments_Original-Submission.pdf

Reviewer-1-Report-Original-Submission -- Alessia Visconti 8/10/2018 Reviewed

Reviewer-2-Report-Original-Submission -- Nicola Segata 8/10/2018 Reviewed

Supplement_Files.zip
