# Supplementary material for: MGS-Fast: Metagenomic shotgun data fast annotation using microbial gene catalogs
Source: Gigascience. 2019 Apr 3;8(4):giz020. doi: 10.1093/gigascience/giz020 (PMC6446249; doi:10.1093/gigascience/giz020)
Supplement: GIGA-D-18-00255_Revision-1.pdf [file giz020_giga-d-18-00255_revision-1.pdf]

GigaScience

MGS-Fast: MetaGenomic Shotgun data Fast annotation using microbial gene catalogs

--Manuscript Draft--

**Manuscript Number:**	GIGA-D-18-00255R1
**Full Title:**	MGS-Fast: MetaGenomic Shotgun data Fast annotation using microbial gene catalogs
**Article Type:**	Technical Note
**Funding Information:**	National Institute on Minority Health and Health Disparities (G12 MD007599) — Dr Konstantinos Krampis<br>Weill Cornell Medical College (2UL1TR000457) — Dr Konstantinos Krampis<br>NYU Langone Medical Center (US) (UH3CA140233) — Dr Stuart M. Brown<br>Association of Chinese American Physicians (U01CA182370) — Dr Zhiheng Pei<br>National Cancer Institute (R01CA159036) — Dr Zhiheng Pei<br>National Institute of Allergy and Infectious Diseases (R01AI110372) — Dr Zhiheng Pei<br>National Institute of Dental and Craniofacial Research (R21DE025352) — Dr Zhiheng Pei
**Abstract:**	Current methods used for annotating metagenomics shotgun sequencing (MGS) data, rely on a computationally intensive and low stringency approach of mapping each read to a generic database of proteins or reference microbial genomes. We developed MGS-Fast, an alternative analysis approach for shotgun whole genome metagenomic data utilizing Bowtie2 DNA-DNA alignment of reads, to the IGC database of well annotated genes compiled from human microbiome data. This method is rapid and provides high stringency matches (>90% DNA sequence identity) of the metagenomics reads to genes with annotated functions. We demonstrate the use of this method with data from a study of liver disease and synthetic reads, and Human Microbiome Project shotgun data, to detect differentially abundant KEGG gene functions in these experiments. This rapid annotation method is freely available as a Galaxy workflow within a Docker image.
**Corresponding Author:**	Konstantinos Krampis, PhD<br>Hunter College<br>New York, NY UNITED STATES
**Corresponding Author Secondary Information:**	
**Corresponding Author's Institution:**	Hunter College
**Corresponding Author's Secondary Institution:**	
**First Author:**	Stuart M. Brown, PhD
**First Author Secondary Information:**	
**Order of Authors:**	Stuart M. Brown, PhD
	Hao Chen, PhD
	Yuhan Hao, PhD
	Bobby P. Laungani, BSc
	Thahmina A. Ali, BSc
	Changsu Dong, MSc

Powered by Editorial Manager® and ProduXion Manager® from Aries Systems Corporation

	Carlos Lijeron, MSc
	Baekdoo Kim, BSc
	Claudia Wultsch, PhD
	Zhiheng Pei, PhD
	Konstantinos Krampis, PhD
**Order of Authors Secondary Information:**	
**Response to Reviewers:**	ATTACHED RESPONSE TO REVIEWERS AS SUPPLEMENTARY .doc FOR CLEARER FORMATTING.
**Additional Information:**	
**Question**	**Response**
Are you submitting this manuscript to a special series or article collection?	No
**Experimental design and statistics**<br><br>Full details of the experimental design and statistical methods used should be given in the Methods section, as detailed in our Minimum Standards Reporting Checklist. Information essential to interpreting the data presented should be made available in the figure legends.<br><br>Have you included all the information requested in your manuscript?	Yes
**Resources**<br><br>A description of all resources used, including antibodies, cell lines, animals and software tools, with enough information to allow them to be uniquely identified, should be included in the Methods section. Authors are strongly encouraged to cite Research Resource Identifiers (RRIDs) for antibodies, model organisms and tools, where possible.<br><br>Have you included the information requested as detailed in our Minimum Standards Reporting Checklist?	Yes
**Availability of data and materials**	Yes

All datasets and code on which the conclusions of the paper rely must be either included in your submission or deposited in publicly available repositories (where available and ethically appropriate), referencing such data using a unique identifier in the references and in the "Availability of Data and Materials" section of your manuscript.<br><br>Have you have met the above requirement as detailed in our Minimum Standards Reporting Checklist?	

Manuscript
Click here to access/download;Manuscript;MGSFast-Manuscript-Gigascience-V5.docx
Click here to view linked References

MGS-Fast: MetaGenomic Shotgun data Fast annotation using microbial gene catalogs

Stuart M. Brown[1], Hao Chen[1], Yuhan Hao[1], Bobby P. Laungani[2,3], Thahmina A. Ali[2,3], Changsu Dong[2,3], Carlos Lijeron[2,3], Baekdoo Kim[2,3], Claudia Wultsch[3,4], Zhiheng Pei[5], Konstantinos Krampis[2,3,6,*]

[1]New York University Langonne Medical Center, New York, NY; [2]Department of Biological Sciences and Center for Translational and Basic Research, Belfer Research Building, Hunter College of The City University of New York, New York, NY; [3]Research Foundation of The City University of New York, New York, NY; [4]Sackler Institute for Comparative Genomics, American Museum of Natural History, New York, NY; [5]Department of Veterans Affairs New York Harbor Healthcare System, New York, NY; [6]Institute of Computational Biomedicine, Weill Cornell Medical College, New York, NY.

ORCID IDs:
Stuart M. Brown: 0000-0002-0906-9907; Bobby P. Laungani: 0000-0003-4970-8192; Thahmina A. Ali: 0000-0002-7350-6163; Konstantinos Krampis: 0000-0002-9155-2390.

*Corresponding Author, email: agbiotec@gmail.com

ABSTRACT

Current methods used for annotating metagenomics shotgun sequencing (MGS) data, rely on a computationally intensive and low stringency approach of mapping each read to a generic database of proteins or reference microbial genomes. We developed MGS-Fast, an alternative analysis approach for shotgun whole genome metagenomic data utilizing Bowtie2 DNA-DNA alignment of reads, to the IGC database of well annotated genes compiled from human microbiome data. This method is rapid and provides high stringency matches (>90% DNA sequence identity) of the metagenomics reads to genes with annotated functions. We demonstrate the use of this method with data from a study of liver disease and synthetic reads, and Human Microbiome Project shotgun data, to detect differentially abundant KEGG gene functions in these experiments. This rapid annotation method is freely available as a Galaxy workflow within a Docker image.

Keywords: Metagenomics, Annotation, Cloud Computing, Docker, Galaxy

BACKGROUND

The initial focus of metagenomics studies, such as the Human Microbiome Project [1] was to survey the microbial communities present in various sites on and in the human body, but the focus of research has now shifted to understanding the functional role these microbes play in metabolic and disease processes. Assessment of the taxonomic diversity and composition of metagenome samples using amplicon sequencing of the 16S rRNA marker gene is inexpensive and has been applied to map a wide variety of microbial communities , but it is also subject to bias and lacks sensitivity below the species level. It is known that individual bacterial isolates with identical 16S genes may differ by as much as 15-30% in their genomes [2], which may include toxin production, antimicrobial, or metabolic genes. Alternatively, metagenomics shotgun sequencing (MGS) of all DNA present in a biological sample can be used for computational prediction of gene functions of sequenced DNA fragments to infer differences in the biological function of microbial communities [3]. Existing bioinformatics tools to characterize MGS data face bottlenecks due to the large computational task of comparing millions of short DNA sequences (50 to 200 nucleotides in length) to various databases of known proteins, conserved protein motifs, or

annotated genomes. The BLAST [4] method used to compare DNA sequences (i.e., reads) to a database, requiring hundreds of CPU hours to analyze a typical MGS sample containing hundreds of millions of reads.
Approaches to overcome this computational bottleneck include the reduction of read data file complexity, for example, through de-duplication or by de novo assembly. However, these data reduction methods themselves require substantial computational effort and can introduce significant bias. Furthermore, mis-assemblies can introduce significant biases, since the WGS reads correspond to hundreds of bacterial genomes and chimeric contigs can be created [5]. This is especially true for gut microbiomes where closely related species with similar genomes are present, and this could be exacerbated in the case where significant gene transfer occur across species (transposase, phage and lateral gene transfer). The result of a misassembly is to distort abundance information, as genomic sequences could be assembled together, resulting in losing the signal for species present in the sample. In addition, one of the advantages of the mapping approach, is that it is possible to recover the presence of a gene even when the coverage for that gene is not sufficient for assembling it. Therefore, gene presence in the sample can be better identified used the raw reads and comparing to annotation databases, rather assemblies that is difficult to ensure that species are not artificially masked during the assembly process.

Other methods involve the use of faster, but less sensitive sequence matching algorithms such as BLAT [6] or RAPSearch (MG-RAST webserver [7]), or reduced databases for functional protein identification, thus providing a less precise assay for microbial protein function. For example, the MG-RAST webserver, the wait queue for data processing can be up to several weeks. Carr and Borenstein [8] compared MGS annotation using BLAST vs. BWA (a DNA sequence similarity tool very similar to Bowtie) and they conclude that at short evolutionary distances, BWA has a higher precision and recall than BLAST for identifying KEGG orthologs, but recall and precision for BWA drops dramatically at greater evolutionary distances.

RESULTS

MGS-Fast Algorithm and Software Implementation.

We created a computationally efficient pipeline for MGS data analysis called MGS-Fast, which combines several data pre-processing steps (read data trimming and filtering of low-quality sequences, removal of human host contaminant sequences), with taxonomic and functional profiling of metagenomic WGS sequence data. The pipeline leverages software broadly used in the bioinformatics community for quality control, taxonomy, DNA sequence alignment, and taxonomic profiling (details in Methods section). The novelty of MGS-Fast is based on the use of stringent DNA-DNA matching to annotated and high-quality bacterial DNA sequences from the integrated catalog of reference genes (IGC) in the human gut microbiome [9]. The IGC database contains 9,879,896 gut microbe genes with annotations based on the Kyoto Encyclopedia of Genes and Genomes (KEGG, http://www.genome.jp/kegg/kegg1.html). The bioinformatics workflow of MGS-Fast utilizes Bowtie2 to rapidly map a MGS dataset and assign known functions to reads originating from microbial genes, producing counts of KEGG gene orthologs as output. The KEGG counts are then applied to identify differentially abundant microbial gene functions in metagenomics datasets, and separate samples from different patient groups (**Fig. 1**). The IGC database is precompiled as a Bowtie 2 index which is deployed automatically during installation of MGS-Fast, and is also available as separate download (Availability section). Furthermore, the MGS-Fast pipeline is packaged as a pre-configured, ready to execute software within a Docker container, that is easy to deploy by non-bioinformatics experts through a single command (Supplementary Information - Software-Manual). Researchers working with non-human MGS gut data, can create their own custom database of microbial genes (Methods section) for functional profiling. Once a custumized database is prepared, MGS-

Fast allows for parallel processing of multiple WGS metagenomic samples with, increasing accuracy and reducing computational time for the functional assignment of reads.

Data Analysis of Liver Cirrhosis Metagenomic Samples.

In our study, the MGS-Fast pipeline was used for the analysis of gut microbiome samples from 10 patients with liver cirrhosis, and 10 control samples from an earlier study by Qin et. al [10], obtained from the European Nucleotide Archive accession ERP005860. Upon completion, the pipeline generated gene function abundance counts, with a total of 3785 KEGG IDs that was similar to the number (4,801) in the original study by Qin et. al. Next, following the recommendations in [11], we analyzed 502 out of 3785 KEGG IDs that had significantly different abundance scores (FDR corrected *P*-value threshold 0.05, Suppl. KEGG-FDR.CSV) as a mixture model with a Negative Binomial distribution, using the R package *edgeR*, version 3.7 [12]. In order to visualize the differences between groups of KEGG ID abundances, we also used *edgeR* to create a multi-dimensional scaling plot (MDS, **Fig. 1**), were clear separation was observed between the healthy vs. cirrhosis samples.

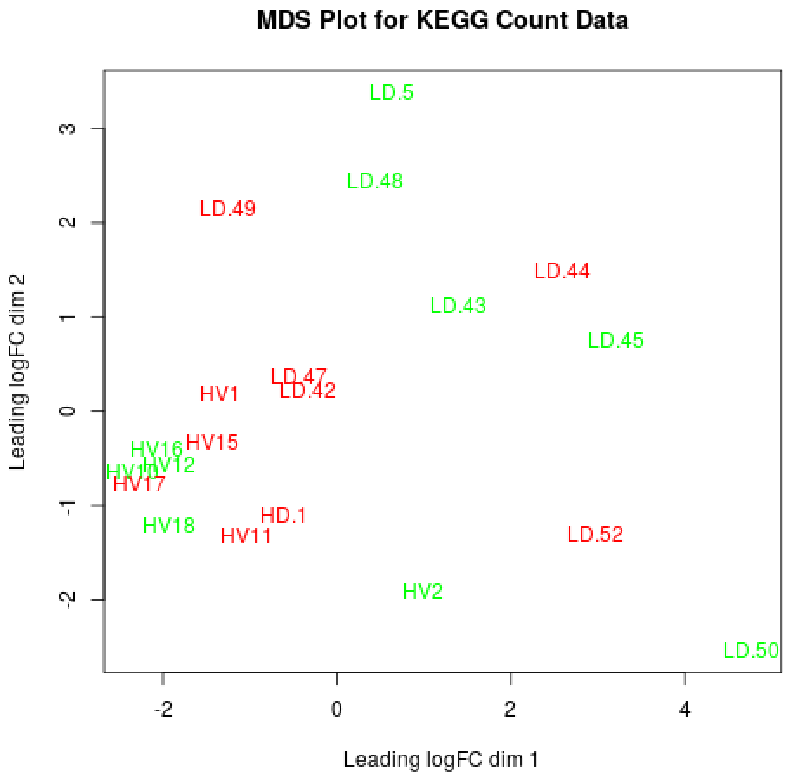

Figure 1. Abundance plot generated using the R package *edgeR* and a mixture model with a Negative Binomial distribution, for the KEGG annotations generated by MGS-Fast using as input data from gut microbiomes of healthy patients (HV) and patients with liver cirrhosis (LD).

Next, we mapped 502 FDR-corrected (0.05) KEGG IDs returned by MGS-Fast to pathways using the KEGG website tools (http://www.genome.jp/kegg/tool/map_pathway2.html). We were able to identify functional groups and pathways modules which corresponded closely to the ones found in the original study by Qin et al. Specifically, the majority of the pathway modules detected were for membrane transport, including oligopeptide transport systems, in addition to zinc, glutamine and energy coupling factor transport (complete list in Suppl. file “KEGG modules - 502 KEGG IDs.doc”). Furthermore, and as reported in the original study we found prevalent pathway modules for carbohydrate, amino-acid and

energy metabolism, including the citrate and Krebs and Calvin cycles, gluconeogenesis, glyoxylate and glycolysis cycle. We also observed a set of liver-cirrhosis-associated markers similarly with Qin et al., including assimilatory nitrate reduction, denitrification, GABA biosynthesis and GABA shunt, in addition to heme biosynthesis. The GABA neurotransmitter system is correlated to brain disease [13] as a result of liver dysfunction, because of increased GABA levels in the blood have the potential to go through the blood–brain barrier and cause hepatic encephalopathy. Finally, we also detected a set of pathway modules for ammonia production, which could lead to increases ammonia levels in the blood as described by the original study (Qin et al. 2014). In this respect, we also found the assimilatory nitrate reduction pathway module to be present, in addition to dissimilatory nitrate reduction and the complete nitrification pathway (Suppl. file "KEGG modules - 502 KEGG IDs.doc").

Comparative Pipeline Performance and Processing Times.

The MGS-Fast Docker container was deployed on an 8-CPU Intel Xeon Server supporting hyper-threading for a total of 8 parallel processes ("threads"), in addition to 128 GB RAM memory. This is a high-performance computing server, commonly found in laboratories performing genome sequencing bioinformatics. In order to compare computational performance of MGS-Fast to other published pipelines for metagenomic annotation, we measured the processing time for each tool in the different pipelines using the patient datasets from our study. For ensuring compatibility of the results we applied the option "--threads 8" or similar for all pipelines (Kraken, GOTTCHA [14-15], and HumanAn2 [https://huttenhower.sph.harvard.edu/humann2]) included in our comparison. The pipelines have been setup according to the documentation for each, using the standard full database for Kraken ("kraken-build --standard --db $DBNAME") and the latest bacterial databases for GOTTCHA (ftp://ftp.lanl.gov/public/genome/gottcha/. Processing times for the MGS-Fast workflow (sample id ERR526291, number of reads 15,181,542 x 2), in comparison to the other pipelines are shown in **Table 1**. Interestingly, most other pipelines compared with MGS-Fast do not perform preprocessing of data such as quality control or removal of host WGS reads, except for GOTTCHA which offers users the option to trim input DNA reads. **Table 1** lists the times required by MGS-Fast for the data pre-processing steps, including read trimming and removing of host sequences. Furthermore, **Fig. 2** reports processing times for all MGS-Fast pipeline steps when used for analysis of different patient metagenomic data sets, which ranged from 1.5 GB to 11GB in size. Similarly, **Fig. 3** provides total time of execution comparison for the same datasets with Kraken that uses a large in-memory k-mer database versus the compressed Burrows–Wheeler Transform (BWT) - Bowtie2 aligner used for MGS-Fast, and DIAMOND using protein based alignment.

Based on our comparison data (**Table 1**), MGS-Fast was approximately three times slower GOTTCHA (49 versus 17 minutes), while it was four and five times faster than HumanAn2 and Kraken (49 versus 162 and 254 minutes respectively) for processing the ERR526291 dataset (15,181,542 x 2 paired reads). For HumanAn2, we observed that the software generates a bowtie index for the reads in the sample provided as input under a temporary files output directory ("$OUTPUT_DIR/$SAMPLENAME_humann2_temp/", also described in its documentation), which might explain the additional time required in comparison to MGS-Fast. The significantly increased time required by Kraken to process the dataset, is not surprising as in the corresponding publication [14], it was reported that Kraken can process 150,000 reads per minute. With 30 million reads in the ERR526291 dataset, it would requires- at least 200 minutes for the alignment, with additional time for the annotation, writing the output and other tasks. Furthermore, the compute server used for running all the software comparisons had ample amount of RAM memory (128GB), and by looking at the Kraken output file we noticed that no page faults were reported (this is a build in feature in the software, where number memory faults - disk swaps are reported), which if was the case would explain the increased time taken by Kraken. The database constructed by Kraken, using the "kraken-build" command that downloads reference data by from NCBI was approximately 164 GB in size.. This is more than twice the size of 70GB reported in the original 2015 publication, and also given

that Kraken is a k-mer based aligners and read sizes have increased(in our dataset it was 100bp x 2 paired end), meaning more k-mers to be compared per read, requiring increased time to process the sample.

Table 1. Processing times (in minutes) for the MGS-Fast pipeline in comparison to other workflows (Kraken, HumanAn2, GOTTCHA) used for WGS metagenomics analysis. Time for data preprocessing steps (quality control of metgenomic data, filtering of host DNA) performed by MGS-Fast is listed in parenthesis.

Time in minutes, with 8x threads	***Dataset ID, number of reads x paired***	***Data QC (FASTQC)***	***Data prep (Groomer)***	***Trimmo matic***	***Human filtering (Bowtie)***	***Taxonomic classification (MetaPhlan)***	***Annotation (Bowtie IGC)***	***KEGG count***	***Total (w/o filtering)***
MGS-Fast	*ERR526291 number of reads: 15,181,542 x 2*	*2*	*6*	*2*	*6*	*23*	*22*	*4*	*49 (16)*
Kraken		*N/A*	*N/A*	*N/A*	*N/A*	*254*			*254*
HumanAn2		*N/A*	*N/A*	*N/A*	*N/A*	*162*			*162*
GOTTCHA		*N/A*	*N/A*	*(*1)*	*N/A*	*17*			*17*

Figure 2. Processing times for all MGS-Fast pipeline steps when used for analysis of different patient metagenomic data sets ranging from 1.5GB to 11GB in size.

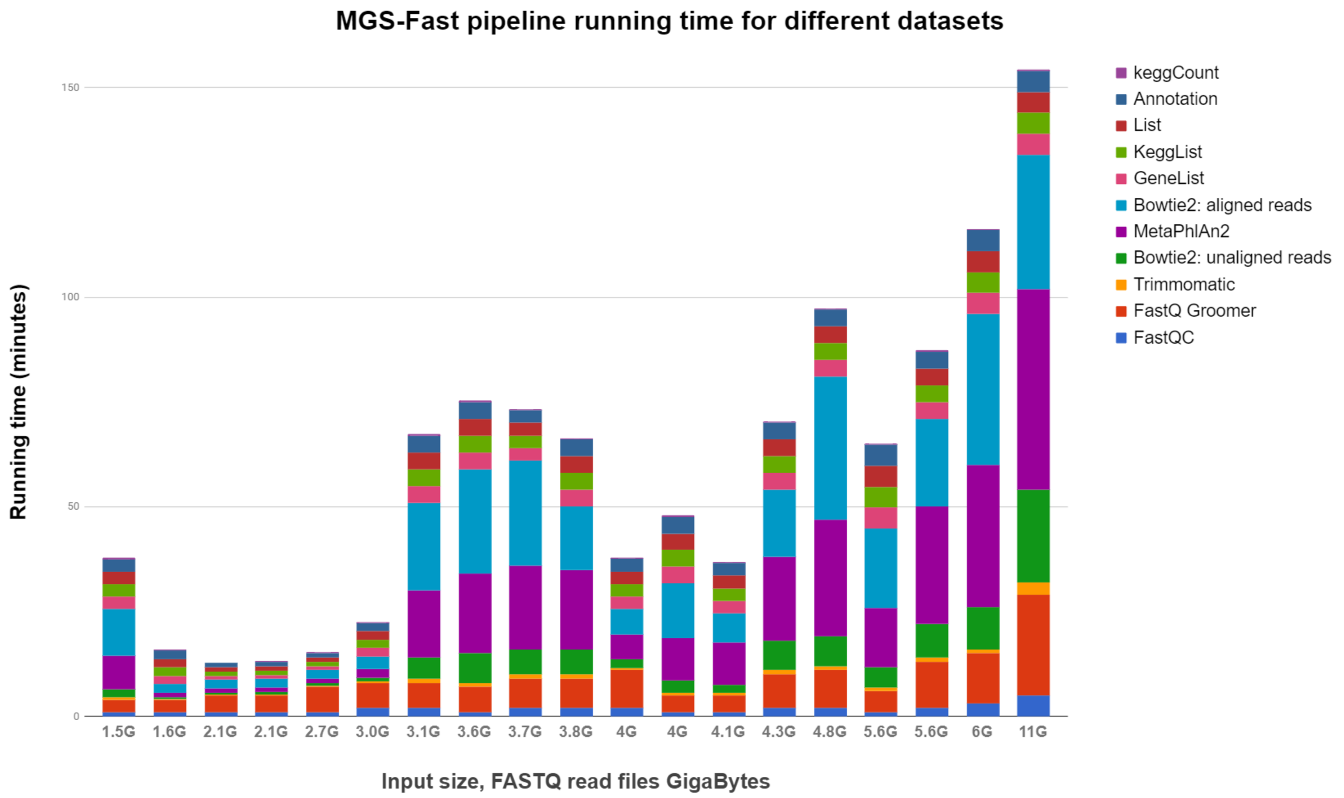

Fig. 3 comparison of MGS-Fast running to Kraken and DIAMOND, using the same database build by the IGC sequence set for each tool. All input samples, compiled database and outputs can be found at http://146.95.173.35:9988/MGS-FAST/.

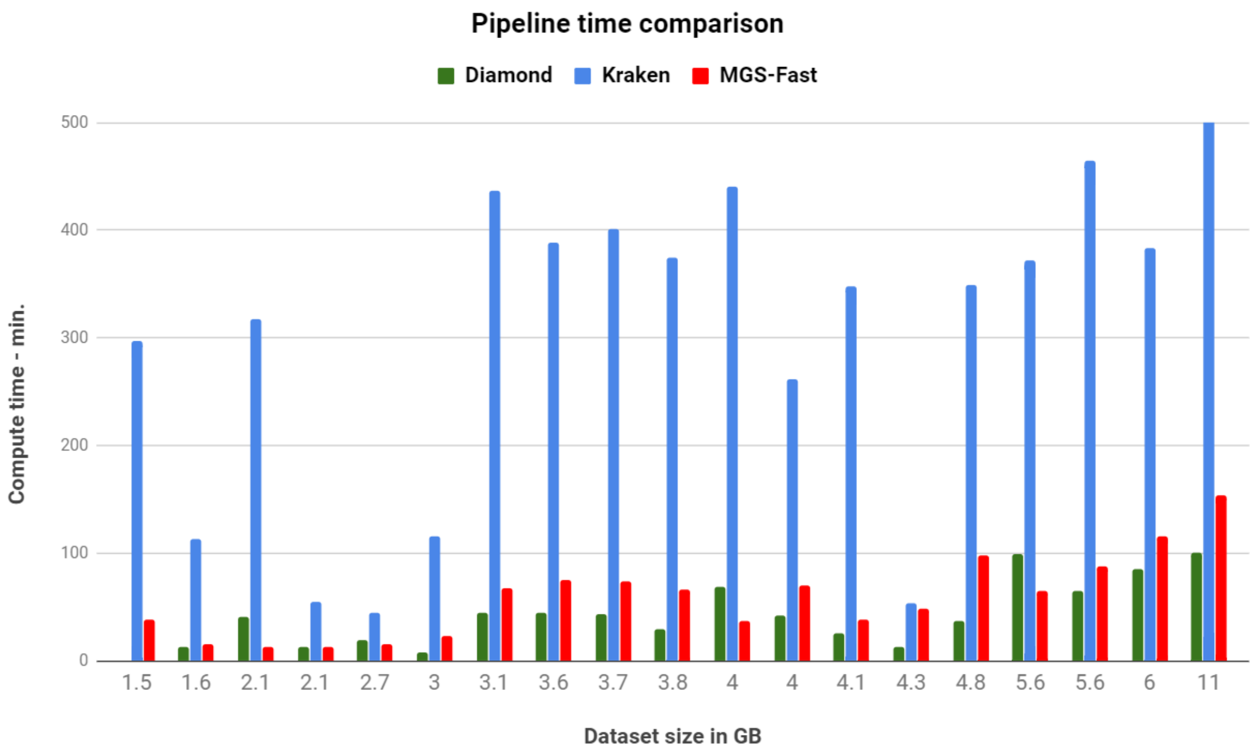

WGS metagenomic datasets used as controls for MGS-Fast.

We evaluated the performance of the MGS-Fast pipeline further by using a range of metagenomic samples from mouse and human collected across different body sites (e.g., gut, mouth, skin), in addition to environmental samples (e.g., copper mine waste), and negative controls of simulated read data from real or synthetic genomes (**Table 2**). The Human Oral Microbiome Database (HOMD, [16]) was included to the analysis workflow as additional annotation data. In more detail, Bowtie2 alignments of human gut (fecal) samples performed by MGS-Fast resulted in 95.62% of all reads in the sample being successfully mapped to the database. For human oral and skin microbiome samples 82.32% and 33.02% % of the reads, respectively (**Table 2**) were mapped to the database.

Simulated FASTQ reads from the human reference genome GRCh38 aligned at only 7.35%, which was expected since our pipeline filters out human sequences. As a positive control, we mapped MetaSim [17] simulated reads from the *E. coli* K12 reference genome (GenBank: accession U00096.3) and 98.5% of the sample was aligned. Furthermore, as negative controls we included the HMP mock microbial community (SRR172902; 28.82% of reads aligned to database), a synthetic metagenome (SRR3732372) made from a mixture of DNA from lab strains of bacteria (10.23% of reads aligned to database), and a copper mine waste sample (MG-RAST accession 4664533.3; 8.69% of reads aligned to database; **Table 2**). Finally, false positive matches were evaluated by aligning a set of randomly generated reads by the XS simulator [18]. As expected, only 0.5% of the sample reads aligned to our database.

Table 3. Bowtie2 alignment of different metagenomic samples to our IGC/HOMD derivative database.

	Metagenome	Sample Accession/Source	% Aligned to database
1	Human gut	SRR2822459	95.62

2	Human gut - liver disease	ENA ERP005860	96.03
3	Mouse gut	MG-RAST 4535626.3	89.71
4	Human mouth	SRS016533	82.32
5	Human esophagus	SRS065335	71.39
6	Human vagina	SRS014465	66.39
7	Human skin	SRR1646957	33.02
8	Human genome GRCh38	MetaSim simulated	7.35 (false positives)
9	*E. coli* K12 genome	MetaSim simulated	98.5
11	HMP Mock	SRR172902	28.82
10	Synthetic microbial reads	SRR3732372	10.23
12	Copper mine waste	MG-RAST 4664533.3	8.69
13	Randomly generated reads	XS simulator	0.53

METHODS

MGS-Fast Pipeline Structure and Data Processing.

The MGS-Fast workflow begins with quality control using FastQC (FastQC , RRID:SCR_014583)(red rectangles, **Fig. 4A**, http://www.bioinformatics.babraham.ac.uk/projects/fastqc), which creates as output a report on many aspects of input data quality. Next, Trimmomatic (Trimmomatic , RRID:SCR_011848)([20], blue rectangle, **Fig. 4A**) is used to remove sequencing adapters, primers and low quality sequence data. Human host DNA is removed by alignment of reads to the human reference genome using Bowtie2 (Bowtie , RRID:SCR_005476)([21], left green rectangle, **Fig. 4A**), using the human GRCh38 reference genome (https://www.ncbi.nlm.nih.gov/grc/human). This filtering step retains only the "unmatched" reads corresponding to the metagenome as specified by the "--un" (unaligned) option from Bowtie2. The retained reads are then aligned to the IGC microbiome gene catalog database with Bowtie2 (right green rectangle, **Fig. 4A**), using the "--end-to-end –sensitive" option, in order to assign KEGG protein function IDs to each read. The IGC database (http://meta.genomics.cn/meta/dataTools) contains approximately 10 million KEGG-annotated microbial genes, collected from 1267 public human gut microbiome samples plus an additional 922 complete annotated prokaryotic genomes. The software versions included in this workflow are the following: FASTQC 0.11.6, Trimmomatic 0.32.1, Bowtie 2.2.6 and MetaPhlAn 2.5.0 (MetaPhlAn, RRID:SCR_004915)[22], with a default pre-set of parameters (details in **Supplementary Manual**) that can be easily adjusted and changed by the users through the Galaxy interface.

(A).

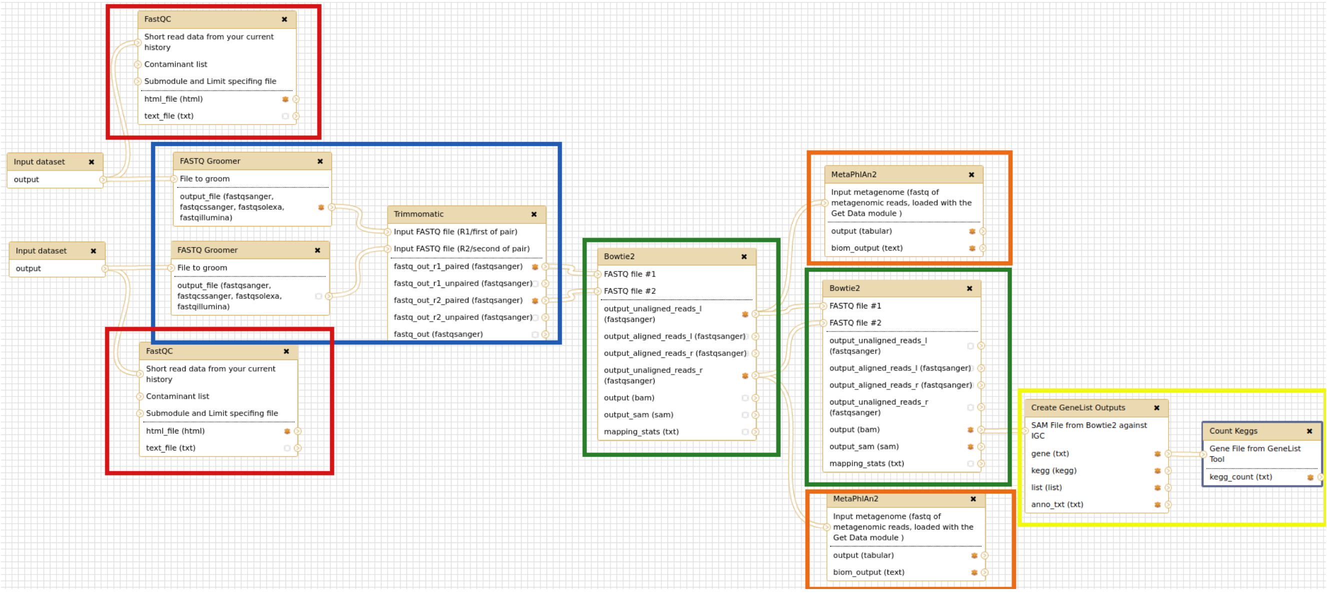

(B).

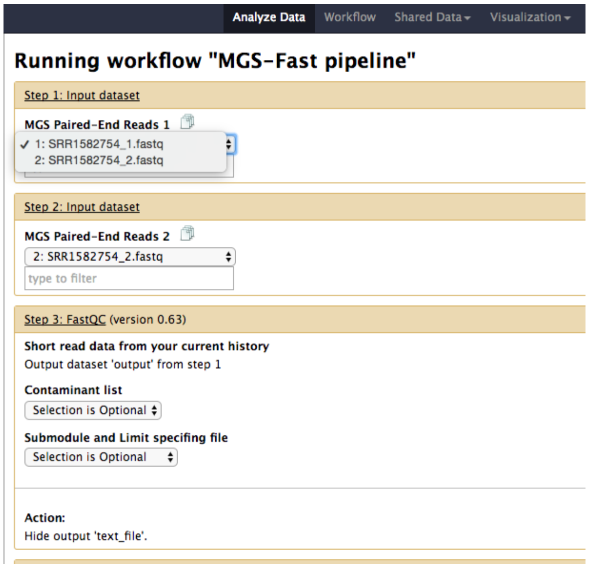

Figure 4. (A). MGS-Fast pipeline on the Galaxy workflow canvas, running on a Docker container. Read quality tools outlined in red, blue for score adjustment and trimming, green for Bowtie2 alignment to the IGC/HOMD or human reference, orange for the Metaphlan analysis and yellow for annotation parsing from the Bowtie2 results; **(B)**.Interface of MGS-Fast pipeline on Galaxy web server running in the Docker container. Users can select the input data and parameters for the pipelines through drop-down menus and input boxes (details in Suppl. Software Manual); **(C).** The pipeline output for the MetaPhlan tool, visualized within the Galaxy web interface

(C).

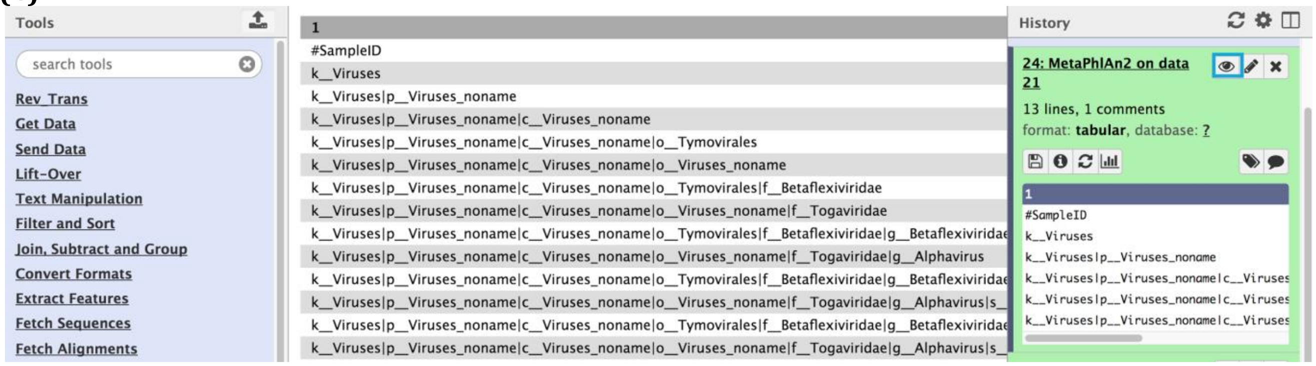

Next, a custom Python script integrated in the workflow (yellow rectangle, **Fig. 4A**) is used to count the number of IGC genes and KEGG IDs generated as output of Bowtie2 with IGC. The script counts the

number of reads aligned to each gene ID in the BAM file generated by Bowtie2, and stores the counts in a two column "gene ID - abundances" file. The annotations were also parsed from the original IGC/HOMD FASTA files, in order to produce a second file of "gene ID - KEGG ID" that was saved as a Python 'dictionary' data structure. The "gene ID - abundances" are then read line by line using the Python script, which also loads the dictionary data structure, and matches the "gene ID - abundance" list entries with these of the "gene ID - KEGG ID" based on the gene ID. The KEGG IDs of the matching lines from the two lists, are then used by the script as a key for a new dictionary containing key-value pairs. The value corresponding to the KEGG ID keys of the dictionary is set to the corresponding abundance count, and also the count is incremented when the KEGG ID has is already present the dictionary as the lists are parsed. Following all data processing steps, MGS-Fast prints the KEGG IDs and read counts for each gene in a text file, which is used as input file for the *EdgeR* script that creates abundance plots ("abundance-plot.R", available at https://github.com/BCIL/MGS-Fast). The R script filters out genes with low counts, keeping those rows where the count per million (cpm) is ≥1 in at least 6 samples. The cpm for mapped reads is essentially counts scaled by the number of fragments sequenced in one million. Furthermore, we used the "calcNormFactors" function from *EdgeR* in our script, which normalizes for RNA composition by finding a set of scaling factors for the library sizes across samples. This essentially re-scales the library size resulting in an "effective" library size, which is then used for abundance calculations. This step helps to remove any further artefacts of read distributions per gene that might be introduced for example at the initial stage of read trimming.

MGS-Fast Pipeline Software Distribution and Data Options.

The MGS-Fast pipeline was developed using the workflow canvas of the Galaxy bioinformatics web server (Galaxy , RRID:SCR_006281)(**Fig. 4A**, [23]), which was first pre-installed and configured to run within a Docker virtual machine container (http://www.docker.com). The Galaxy web server was chosen since it provides an intuitive, web-browser interface for non-technical users. Users can easily access and run the MGS-Fast pipeline via the Galaxy interface and developers can use the Galaxy workflow canvas to build and modify the pipeline.

Our goal was to develop a complete software bundle within a docker container, which includes the MGS-Fast workflow and all required bioinformatics software, in addition to all other software dependencies. The entire pipeline is implemented in a series of steps (**Fig. 4A**), which are automated via Galaxy. The users only need to select the input datasets (**Fig. 4B**), and the Galaxy workflow engine will automatically execute all remaining analysis steps of the pipeline. Furthermore, the input data directory is attached and automatically available through the Galaxy interface when users set up MGS-Fast and specify the data directories (Suppl. Software Manual). Upon completion of an MGS-Fast pipeline run, the users can download all result files, or simply view the output within the Galaxy interface (**Fig. 4C**). Furthermore, users can run MGS-Fast by reconfiguring the analysis steps, or rerunning a single tool instead of the whole pipeline.

MGS-Fast also provides two options for users to create custom Bowtie2 indexes, for filtering both host genome reads and for annotating the metagenomic reads. Through the first option users can specify the location of a file containing the sequence of a host genome or metagenome, through a text-based menu during the initial run of the MGS-Fast container (Suppl. Manual). The scripts inside the container will automatically build an index for the provided genome, and make it available for use on the Galaxy interface without any futher effort by the user. As a second option, we made an additional pipeline (Suppl. Material) available which is called "Galaxy-Workflow-Custom_MGS-Fast.ga" and can be imported to an already installed and running MGS-Fast workflow. The custom workflow is identical with the regular workflow used by MGS-Fast, but provides users the option to use a FASTA file containing the sequence(s) of the custom genome as input. The Bowtie2 index for the provided genome is automatically built during the first run of the workflow, and is then made available for all subsequent runs. Similarly, users can at

any point add additional custom genome indexes, both for filtering host DNA reads and for classifying metagenomes. Users can download host genomes (e.g., mouse reference https://www.ncbi.nlm.nih.gov/genome/?term=Mouse), and also a range of WGS metagenomes from the Joint Genome Institute [https://img.jgi.doe.gov/cgi-bin/m/main.cgi] .

DISCUSSION

MGS-Fast can confidently transfer functional annotations from annotated gene databases to sequence reads in metagenomic datasets. For microbial read annotation and assignment of KEGG IDs by alignment to the International Genome Consortium (IGC), the MGS-Fast uses the Bowtie2 algorithm requiring by default 90% DNA sequence identity in finding matches. While the DNA to DNA alignment performed by Bowtie2 is less sensitive than translated BLAST utilizing information from conservative amino acid substitutions, at the 90% level of identity, we only have exact matches from DNA fragments of the same species, or orthologs between closely related species [24]. We have also considered increasing the sensitivity of our method, by changing the Bowtie2 parameters and including the "--very-sensitive-local" parameter or increasing the number of allowed mismatches, but decided against it since it would allow less stringent DNA-DNA alignments and more false positives. Nonetheless, this option is still available for users as the Bowtie2 parameters can be easily adjusted through the Galaxy interface, when initiating a MGS-Fast pipeline run. However, caution is required in interpreting the results with increased sensitivity parameters, since a high percentage of false positive alignments will make assignment of metabolic function to metagenomic reads less reliable. In addition to Bowtie2 our pipeline also provides annotations through MetaPhlAn2, using a database of approximately one million unique clade-specific marker genes from 17,000 reference genomes. This enables MGS-Fast to identify taxa within narrow clades, even in the absence of reference genomes for species in the gut community. MetaPhlAn2 enabled us to identify a microbial organism at higher taxonomic levels of genera or family, and we observed identification of 8931 organisms, out of which 6681 have been annotated by MetaPhlan at the order taxonomic level (“ceae”).

Using Docker container technology, we bundled all required software components and the MGS-Fast pipeline as a pre-configured, ready to use bioinformatics package for performing standardized, automated metagenomics analysis on any desktop or laptop computer running Windows, MacOS or the Linux operating system. Both the container and source code are publicly available for download (Availability section), and can be easily installed by non-bioinformatics experts with a single command (**Suppl.** Software-Manual). Users can then simply access the MGS-Fast pipeline via the Galaxy interface by entering the network address of the container (made available to the user when the installation is complete) on their web browser. Furthermore, the Docker container can be deployed on the cloud or institutional clusters, where users can run multiple instances of MGS-Fast in parallel in order to process multiple NGS samples, or within a single instance of MGS-Fast using Galaxy’s Data Collections input data options .

Regarding computational performance for large-scale studies, we tested MGS-Fast with a set of Illumina HiSeq 2000 oral microbiome datasets ranging from 1.5GB to 11GB (**Fig. 2**) in file size (https://www.ebi.ac.uk/ena/data/view/SRX978361). Using a compute server with average computational capacity (128GB, 8 CPU core) the processing time for MGS-Fast ranged from 20 minutes for smaller read sets to 2.5 hours for the larger ones. The cumulative processing time to complete running MGS-Fast for all datasets included in this study was approximately 15 hours (900 minutes). Processing times for large WGS metagenomic studies (100 samples), the complete study can be processed in the course of a few days. While running a single dataset at a time on our computer server, we noticed that the hardware capacity was underutilized and decided to implement MGS-Fast analysis in parallel, reducing

the total time required to process the datasets included here. In a production setting, where also more computational capacity might be available, researchers could use tens of instances at the same time and efficiently process large-scale data sets.

We then compared the performance of the Burrows–Wheeler Transform (BWT) MGS-Fast used for MGS-Fast. (**Fig. 3**) with Kraken [14] and DIAMOND [25] that use respectively large in-memory k-mer database and using protein based alignment, versus the compressed Burrows–Wheeler Transform (BWT) - Bowtie2 aligner used for MGS-Fast. The Kraken approach is based on a database of pre-classified k-mers, and although it classify millions of reads in just a few minutes, their memory requirements are usually high requiring a high-performance, expensive compute server that some labs do not have access to in order to be able to complete the analysis. In contrast, MGS-Fast similar to multiple other published tools in the literature using the more efficient Bowtie2 index structure, which allows for memory efficiency storage and increased speed when querying the database [26], as also evidenced by our results in the present study.

Furthermore, our results are substantiated by the a subsequent release by the authors of Kraken of a newer tool called Centrifuge [27], that unlike Kraken and similar to other nucleotide-based classification tools in the literature is using is also the Burrows–Wheeler transform (BWT) for the genome database. This strategy uses one-tenth the space of a Kraken index for the same database, providing faster classification speed and lower memory requirements, making it possible to perform large-scale metagenomics annotation on a desktop computer. As reported in this study [27], Centrifuge took only 47 min on a standard desktop computer to analyze a total of 26 GB of input sequence data, and where Kraken and MegaBLAST required 100 GB and 25 GB of memory respectively for their indexes, Centrifuge requires only 4.2 GB. Similar results have been reported for multi-fold increased speed in comparison to Kraken with BWT use in PALADIN and Kaiju [28-29], and as reported in these studies an important constraint for Kraken is its memory usage, where the database grows in linear proportion to the number of distinct k-mers in the genomic library (at 12 bytes per k-mer).

Regarding using nucleotide read alignment versus protein translated search for metagenomic classification, the fact is that amino acid sequences are conserved better at evolutionary distances, leading to more sensitive read classification in the case of distant species or tax. Both DIAMOND and Kaiju [25, 29] align six-frame translations of reads against a protein database. Similarly to what was mentioned above, Kaiju indexes the reference protein database using BWT as does our MGS-Fast tool, allowing metagenomic sequences to be searched fast and with low-memory footprint against a large protein database. Given a metagenomic sample and the pre-built index, Kaiju first translates every read in all six reading frames, splitting the read at stop codons. As reported in this study, by using the BWT as an index for the reference protein database, Kaiju classifies up to millions of reads per minute and is typically faster than k-mer-based methods such Kraken and Clark [30].

DECLARATIONS

Competing Interests

The authors declare that they have no competing interests.

Authors' contributions

The construction and testing of the MGS-Fast method was implemented by S. Brown with assistance from Y. Hao and H. Chen. The Docker image for MGS-Fast was built by B. Laungani with assistance from T. Ali, C. Dong, C. Lijeron, and B. Kim. The data analysis was performed and the manuscript text was written by K.

Krampis, S. Brown and C. Wultsch. The supplemental software manual was written jointly by B. Laungani with additions by K. Krampis, T. Ali, C. Dong, C. Lijeron, B. Kim and C. Wultsch.

Availability of software and materials

- GitHub repository with MGS-Fast code: https://github.com/BCIL/MGS-Fast
- Docker repository with the MGS-Fast container: https://hub.docker.com/r/bcil/metagenome/tags/ (bcil/metagenome:nyu_4.0)
- IGC Indexes Database: http://www.hpc.med.nyu.edu/~browns02/meta/
- Human metagenomic reads, synthetic data and E.coli datasets: http://www.hpc.med.nyu.edu/~browns02/meta/
-Testing datasets and precompiled genome indexes: http://bioitcore.hunter.cuny.edu:9988/metagenomics_package.tar.gz
- Furthermore, detailed instructions on the use of the Docker system and installation and use of the MGS-Fast image are available in the software manual as part of this manuscript.
-All software, indexes and containers are released under open-source MIT licence.

Availability of supporting data

Snapshots of the code and sample data are also available in the *GigaScience* GigaDB repository[31].

List of Abbreviations

HOMD - Human Oral Microbiome Database
IGC - Integrated catalog of reference genes in the human gut microbiome
KEGG - Kyoto Encyclopedia of Genes and Genomes
MGS - Metagenomic Shotgun Sequencing
PCR - Polymerase Chain Reaction
WGS - Whole Genome Sequencing

Acknowledgements

Supported by CTBR NIMHD award G12 MD007599, WCMC-CTSC 2UL1TR000457, NYU Langone Medical Center, Assoc. of Chinese American Physicians, NCI, NIAID, NICDR awards UH3CA140233, U01CA182370, R01CA159036, R01AI110372, R21DE025352. The content is sole responsibility of the authors and does not represent the views of NIH, the U.S. Department. of Veteran Affairs or the U.S. Government.

References

[1] NIH HMP Working Group, The NIH Human Microbiome Project. Genome Res. 2009 Dec;19(12):2317-23. PMID: 19819907

[2] Thompson JR, Pacocha S, Pharino C, Klepac-Ceraj V, Hunt DE, Benoit J, Sarma-Rupavtarm R, Distel DL, Polz MF. Genotypic diversity within a natural coastal bacterioplankton population. Science. 2005 Feb 25;307(5713):1311-3. PubMed PMID: 15731455.

[3] Glass EM, Wilkening J, Wilke A, Antonopoulos D, Meyer F. Using the metagenomics RAST server (MG-RAST) for analyzing shotgun metagenomes. Cold Spring Harbor Protoc. 2010 Jan;2010(1):pdb.prot5368.

[4] Altschul, S.F., Madden, T.L., Schäffer, A.A., Zhang, J., Zhang, Z., Miller, W. & Lipman, D.J. (1997) "Gapped BLAST and PSI-BLAST: a new generation of protein database search programs." Nucleic Acids Res. 25:3389-3402. PMID: 9254694

[5] Teeling H, Glöckner FO. Current opportunities and challenges in microbial metagenome analysis—a bioinformatic perspective. Briefings in Bioinformatics. 2012 Sep 8;13(6):728-42.

[6] Kent WJ. BLAT--the BLAST-like alignment tool. Genome Res. 2002 Apr;12(4):656-64. PMID: 11932250

[7] Wilke A, Bischof J, Gerlach W, Glass E, Harrison T, Keegan KP, Paczian T, Trimble WL, Bagchi S, Grama A, Chaterji S, Meyer F. The MG-RAST metagenomics database and portal in 2015. Nucleic Acids Res. 2016 Jan 4;44(D1):D590-4. PMID: 26656948

[8] Carr R, Borenstein E. Comparative analysis of functional metagenomic annotation and the mappability of short reads. PLoS ONE. 2014 Aug 22;9(8):e105776. PMID: 25148512

[9] Li J, Jia H, Cai X, Zhong H, Feng Q, et al; MetaHIT Consortium. An integrated catalog of reference genes in the human gut microbiome. Nat Biotechnol. 2014 Aug;32(8):834-41. PMID: 24997786.

[10] Qin N, Yang F, Li A, Prifti E, Chen Y et al. Alterations of the human gut microbiome in liver cirrhosis. Nature. 2014 Sep 4;513(7516):59-64. PMID: 25079328.

[11] McMurdie PJ, Holmes S. Waste not, want not: why rarefying microbiome data is inadmissible. PLoS Comput Biol. 2014 Apr 3;10(4):e1003531. doi: 10.1371/journal.pcbi.1003531. PubMed PMID: 24699258

[12] Robinson MD, McCarthy DJ, Smyth GK. edgeR: a Bioconductor package for differential expression analysis of digital gene expression data. Bioinformatics. 2010 Jan 1;26(1):139-40. PMID:19910308

[13] Kim YS, Yoon BE. Altered GABAergic Signaling in Brain Disease at Various Stages of Life. Experimental Neurobiology. 2017 Jun 1;26(3):122-31.

[14] Wood DE, Salzberg SL. Kraken: ultrafast metagenomic sequence classification using exact alignments. Genome Biology. 2014 Mar;15(3):R46.

[15] Freitas TA, Li PE, Scholz MB, Chain PS. Accurate read-based metagenome characterization using a hierarchical suite of unique signatures. Nucleic Acids Research. 2015 Mar 12;43(10):e69-.

[16] Chen T, Yu WH, Izard J, Baranova OV, Lakshmanan A, Dewhirst FE. The Human Oral Microbiome Database: A web accessible resource for investigating oral microbe taxonomic and genomic information. 2010. Database, Vol. 2010, Article ID baq013.

[17] Richter DC, Ott F, Auch AF, Schmid R, Huson DH. MetaSim—a sequencing simulator for genomics and metagenomics. PLoS ONE. 2008 Oct 8;3(10):e3373.

[18] Pratas D, Pinho AJ, Rodrigues JM. XS: a FASTQ read simulator. BMC Research Notes. 2014 Jan 16;7(1):40.

[19] Kanehisa M, Goto S. KEGG: Kyoto Encyclopedia of Genes and Genomes. Nucleic Acids Res. 2000 Jan 1;28(1):27-30. PMID: 10592173

[20] Bolger AM, Lohse M, Usadel B. Trimmomatic: a flexible trimmer for Illumina sequence data. Bioinformatics. 2014 Aug 1;30(15):2114-20. PMID: 24695404

[21] Langmead B, Salzberg SL. Fast gapped-read alignment with Bowtie 2. Nat Methods. 2012 Mar 4;9(4):357-9. PMID: 22388286

[22] Segata N, Waldron L, Ballarini A, Narasimhan V, Jousson O, Huttenhower C. Metagenomic microbial community profiling using unique clade-specific marker genes. Nat Methods. 2012 Jun 10;9(8):811-4. PMID: 22688413

[23] Afgan E, Baker D, Van den Beek M, Blankenberg D, Bouvier D, Čech M, Chilton J, Clements D, Coraor N, Eberhard C, Grüning B. The Galaxy platform for accessible, reproducible and collaborative biomedical analyses: 2016 update. Nucleic Acids Research. 2016 May 2;44(W1):W3-10.

[24] Konstantinidis KT, Ramette A, Tiedje JM. The bacterial species definition in the genomic era. Philosophical Transactions of the Royal Society B: Biological Sciences. 2006;361(1475):1929-1940. doi:10.1098/rstb.2006.1920.

[25]. Buchfink B, Xie C, Huson DH. Fast and sensitive protein alignment using DIAMOND. Nature methods. 2014 Nov 17;12(1):59.

[26]. Breitwieser FP, Lu J, Salzberg SL. A review of methods and databases for metagenomic classification and assembly. Briefings in bioinformatics. 2017 Sep 23.

[27]. Kim D, Song L, Breitwieser FP, Salzberg SL. Centrifuge: rapid and sensitive classification of metagenomic sequences. Genome research. 2016 Oct 17.

[28]. Westbrook A, Ramsdell J, Schuelke T, Normington L, Bergeron RD, Thomas WK, MacManes MD. PALADIN: protein alignment for functional profiling whole metagenome shotgun data. Bioinformatics. 2017 Jan 31;33(10):1473-8.

[29]. Menzel P, Ng KL, Krogh A. Fast and sensitive taxonomic classification for metagenomics with Kaiju. Nat Commun. 2016 Apr 13;7:11257. doi: 10.1038/ncomms11257.

[30]. McIntyre AB, Ounit R, Afshinnekoo E, Prill RJ, Hénaff E, Alexander N, Minot SS, Danko D, Foox J, Ahsanuddin S, Tighe S. Comprehensive benchmarking and ensemble approaches for metagenomic classifiers. Genome biology. 2017 Dec;18(1):182.

[31]. Brown SM; Chen H; Hao Y; Laungani BP; Ali TA; Dong C; Lijeron C; Kim B; Wultsch C; Pei Z; Krampis K (2019): Supporting data for "MGS-Fast: MetaGenomic Shotgun data Fast annotation using microbial gene catalogs" GigaScience Database. http://dx.doi.org/10.5524/100557

Click here to access/download
Supplementary Material
V2 Suppl-Software-Manual.docx

R script - EdgeR plot

Click here to access/download
Supplementary Material
edger.r

Click here to access/download
Supplementary Material
Suppl-KEGG-FDR.CSV

Click here to access/download
Supplementary Material
KEGG modules - 502 KEGG IDs.docx

Click here to access/download
Supplementary Material
Galaxy-Workflow-Parallel_MGS-Fast.ga

Compute times comparison

Click here to access/download
Supplementary Material
compute_times_compared.xlsx

Response to Reviewer Comments
Click here to access/download;Personal Cover;MGSFast-reviewers-response-v5.docx

Revisions - Resubmission 2 for Manuscript #GIGA-D-18-00255

Reviewer or editor comment.
Response by the authors.

Reviewer #1:

The authors appreciate the feedback and comments by the reviewer, which helped us to significantly improve the manuscript following the initial submission.

Minor Comments:

1. There are no units in Table 2. This reviewer suggests adding to the Table the information that the figures represent minutes.

We have updated ***Table 2*** *(leftmost column, top row) with the information that the figures represent minutes. We have also added one more* ***Fig. 3*** *with detailed comparison in minutes for 20 samples between our tool MGS-Fast and Kraken (nucleic acid based search) in addition to Diamond (protein based search).*

2. In this reviewer's opinion, Figure 2 will improve if the authors sorted the bars by increasing size of the input files.

*We have sorted the bars by increasing size of the input files to represent the data better in the graph. Overall, it appears to be a linear relationship in the time taken to complete the pipeline in relation to the sample size, byt there are some datasets that deviate from the linear pattern. This is expected given the variability that is inherent in the composition of each WGS metagenomic dataset - similarly this pattern was observed with the other tools (Kraken, Diamond) which we have run and included in our comparison (****Fig. 3****) in this resubmission.*

Reviewer #2 - response to comments from 2nd resubmission :

The authors appreciate the feedback and comments by the reviewer, which helped us to significantly improve the manuscript following the initial submission.

1. Because this is a re-submission of a previous version of the work, I was expecting a point-by-point response to the issues raised by the reviewers, but I didn't find such document in the submission material.

The authors presume that the reviewer possibly missed the extensive point by point address to his / her initial comments in the first resubmission, due to entering these only in the text field for reviewer comments of the manuscript central submission website (as advised by the journal), and not as an attached document with the submitted files for the manuscript. Nonetheless, in the present submission we have also included the response to the points of the reviewer from the first resubmission further below, and we will also included all responses as a .doc file along with the rest of the files for the updated manuscript.

2. The authors applied the method on 20 samples from a larger study with hundreds of samples. Current metagenomic studies generally have hundreds of samples.

*This is a point brought by the reviewer in the first resubmission whether our method "is readily applicable to the number of metagenomes (typically >100) present in current metagenomic investigations". This has been addressed already in the first resubmission (included again below) by reporting the times taken by the various tools of MGS-Fast for metagenomic datasets ranging from 1.5GB to 11GB (as a **Fig. 2** added in the manuscript), to complete study of a WGS microbiome dataset (https://www.ebi.ac.uk/ena/data/view/SRX978361). The times ranged from 20 minutes for the smaller read sets, to 2.5 hours for the largest files. The cumulative time to process all the datasets was approximately 15 hours (900 minutes). Therefore, for five times larger WGS metagenomic studies of 100 samples requested by the reviewer, the complete study could be processed in the course of a few days. The authors stand by the position that reporting the numbers for 20 samples and extrapolating to 100 is more than adequate, given the fact than none of the other published studies in the literature for Kraken, Centrifuge, HumanAn2, DIAMOND, PALADIN, GOTTCHA referenced in the manuscript discussion, present data for that number of 100 samples requested by the reviewer (usually the data reported in these studies is for a small number of samples).*

3. For a fair comparison on the performances, the authors should have used the same database for all the used methods.

*As part of the second resubmission, we have added an additional new figure (**Fig. 3**) and supplementary file with all the data for comparison of MGS-Fast to Kraken and DIAMOND, all with the same database as requested by the reviewer. The reviewer can see details of all the IGC version of the database compiled for each tool, input samples and outputs at http://146.95.173.35:9988/MGS-FAST/. We also have added new paragraphs with explanation and references from the literature for the observed results in the manuscript discussion, and also this is briefly discussed in point 4 below.*

4. It is known that Kraken is faster than BowTie2, so I would expect the proposed pipeline to be slower when using the same database.

The authors believe this is not correct based on information found in the literature for the Bowtie2 / Burrows–Wheeler Aligner (BWA) / Burrows–Wheeler Transform (BWT) used for MGS-Fast. Specifically the authors of Kraken Breitwieser FP, Lu J, Salzberg SL. have stated in a 2017 manuscript (citation below) that the "... the Burrows–Wheeler transform (BWT) and an FM-index to store and index the genome database. This strategy uses only about one-tenth the space of a Kraken index for the same database." We have also added two additional paragraphs in the end of the discussion section of the paper on this point.

- *Breitwieser FP, Lu J, Salzberg SL. A review of methods and databases for metagenomic classification and assembly. Briefings in Bioinformatics. 2017 Sep 23.*

Another example are the data in the paper by Menzel et al. in Nature Comm. 2016 where it was stated that "by using the BWT as an index for the reference protein database, Kaiju is fast enough for classifying up to millions of reads per minute ... and is typically faster than the two k-mer-based methods Kraken and Clark.... In addition, the memory-efficient implementation of the FM-index and suffix array make Kaiju's memory footprint small enough (below 6 GB...) for running it on a standard PC".

- *Menzel P, Ng KL, Krogh A. Fast and sensitive taxonomic classification for metagenomics with Kaiju. Nature communications. 2016 Apr 13;7:11257.*

We have clearly seen this fact in our experiments as well, with Kraken taking 70GB of memory to store its index and requiring a high-performance, expensive compute server that some labs do not have access to in order to be able to complete the analysis, in contrast to MGS-Fast and other tools using the more efficient Bowtie2 index structure. Additional examples similar to these are available in other citations in the literature as well.

Furthermore, our results are substantiated by the a subsequent release by the authors of Kraken of a newer tool called Centrifuge, that unlike Kraken and similar to other nucleotide-based classification tools in the literature is using is also the Burrows–Wheeler transform (BWT) for the genome database. This strategy uses one-tenth the space of a Kraken index for the same database, providing faster classification speed and lower memory requirements, making. As reported in this study , Centrifuge took only 47 min on a standard desktop computer to analyze a total of 26 GB of input sequence data, and where Kraken and MegaBLAST required 100 GB and 25 GB of memory respectively for their indexes, Centrifuge requires only 4.2 GB. Similar results have been reported for multi-fold increased speed in comparison to Kraken with BWT use in PALADIN and Kaiju cited below.

- *Kim D, Song L, Breitwieser FP, Salzberg SL. Centrifuge: rapid and sensitive classification of metagenomic sequences. Genome research. 2016 Oct 17.*

- *Westbrook A, Ramsdell J, Schuelke T, Normington L, Bergeron RD, Thomas WK, MacManes MD. PALADIN: protein alignment for functional profiling whole metagenome shotgun data. Bioinformatics. 2017 Jan 31;33(10):1473-8.*

- *Kaiju (Menzel et al. 2016), a protein homology-based classifier, and Centrifuge (Kim et al. 2016) have addressed the issue of memory consumption*

5. A validation with mock communities on the task of functional profiling is needed to convince a potential user.

*We have performed validation with mock communities from the HMP since the first version of the manuscript (**Table 3**), in addition to synthetic microbial reads,environmental samples (e.g., copper mine waste) and simulated read data from real or synthetic genomes (**Table 3**). In all these cases the matches were from 0.5% up to 28% of our test samples, verifying that our method works properly without functionally annotating false positives in the mock communities.*

6. Line 57. It i not true that "BLAST [4], is the most commonly used" approach to map reads against references. I would say that BLAST is not used at all for metagenomic reads.

We have removed this statement from the manuscript as per reviewer's suggestion.

7. The second paragraph of the "Background" is a bit confusing. There are advantages in using assembly to profile genes, and the real advantages of the mapping approach are not clearly state.

We have updated the background paragraph with the point raised by the reviewer, that by mapping it is possible to recover the presence of a gene even when the coverage for that gene is not sufficient for assembling it.

<u>Reviewer #2 - response to comments from 1st resubmission :</u>

(included again as reviewer might have missed from 1st resubmission)

1. There is no comparison of the pipeline with other available alternatives.

See response to comment 2 below, the pipeline has been compared both in its computational performance, to other similar tools performing WGS metagenomic data analysis and annotation. Similarly, discussion on comparison with available tools can be found in the comment 4 below, and this discussion has been also added to the manuscript, in order to provide readers with an overview and comparison of MGS-Fast with other WGS metagenomics pipelines.

2. Several tools for mapping reads against reference genomes specifically for metagenomics are available (Kraken, CLARK, GOTTCHA, others) and they should be considered (they are frequently faster than BowTie2).

For the comparison of different metagenomic annotation pipelines we used an 8-CPU Xeon Server, with 128 GB RAM, supporting hyper-threading for a total of 8 parallel processes ("threads"). In order to compare computational performance of MGS-Fast with other published pipelines, we measured the time taken by each tool using one of the patient datasets from our study (sample id ERR526291, number of reads 15,181,542 x 2). We used the option "--threads 8" (or similar) for each of the other pipelines of Kraken, HumanAn2 and GOTTCHA included in our comparison. The results on time required for each tool in the MGS-Fast pipeline to run are summarized on the table below (which has been also added to the manuscript). It should be noted here that the pipelines we compared with MGS-Fast do not perform quality control or removal of host reads and require using an external

program for this purpose, except the GOTTCHA that provides only a trimming option. The parts of the MGS-Fast pipeline performing the MetaPhlan Classification, Bowtie2 alignment to IGC, and KEGG counts, correspond to the metagenomic annotation steps taken for by other pipelines included in our comparison. The total time required by MGS-Fast to perform the classification and KEGG annotation steps is shown on the table below, and in parenthesis we have included the additional time required for the removing host sequences and performing read trimming. Furthermore, we have included an additional figure in the manuscript, where we report in detail the time taken by the various steps of MGS-Fast for our set of patient metagenomic data that ranged from 1.5GB to 11GB in size.

time in min, run with 8x threads	***Dataset ID, number of reads x paired***	***Data QC (FASTQC)***	***Data prep (Groomer)***	***Trimmo matic***	***Human filtering (Bowtie)***	***Classification (MetaPhlan)***	***Annotation (Bowtie IGC)***	***KEGG count***	***Total (w/o filtering)***
MGS-Fast	*ERR526291 number of reads: 15,181,542 x 2*	*2*	*6*	*2*	*6*	*23*	*22*	*4*	*49 (16)*
Kraken		*N/A*	*N/A*	*N/A*	*N/A*	*254*			*254*
HumanAn2		*N/A*	*N/A*	*N/A*	*N/A*	*162*			*162*
GOTTCHA		*N/A*	*N/A*	*(*1)*	*N/A*	*17*			*17*

The pipelines have been setup according to the documentation for each, using the standard full database for Kraken ("kraken-build --standard --db $DBNAME") and the latest bacterial databases for GOTTCHA (ftp://ftp.lanl.gov/public/genome/gottcha/. As shown on the table above MGS-Fast was approximately three times slower GOTTCHA (49 versus 17 minutes), while it was four and five times faster than HumanAn2 and Kraken (49 versus 162 and 254 minutes respectively) for processing the ERR526291 dataset. For HumanAn2, we observed that the software generates a bowtie index for the reads in the sample provided as input under a temporary files output directory ("$OUTPUT_DIR/$SAMPLENAME_humann2_temp/" as also described in that software's documentation), which might explain the additional time required in comparison to MGS-Fast. The significantly increased time required by Kraken to process the same dataset as MGS-Fast, which is not surprising given the 150,000 reads per minute reported for this software in the corresponding publication, and with 30 million reads in our dataset it would requires at least 200 minutes for alignment. Furthermore, the compute server used for running all the software comparisons had ample amount of RAM memory (128GB), and by looking at the Kraken output file we noticed that no page faults were reported (this is a build in feature in the software, where memory faults and disk swap are reported), which would explain the increased time to complete. We have also tried a second server and found similar results. We then looked in the database constructed by Kraken using the "kraken-build" command, which downloaded reference data by from NCBI and build a index of approximately 164 GB in size, including supporting files. This database is more than twice the size of 70GB reported in the original 2015 publication. Furthermore, Kraken is k-mer based and read sizes have also increased significantly in recent years (in our dataset it was 100bp x 2 paired end), meaning more k-mers per read, and more comparisons to the also larger database, which explains the increased time required to process the sample.

3. The pipeline is applied on few metagenomes only. The authors thus fail here to show that their pipeline is readily applicable to the number of metagenomes (typically >100) present in current metagenomic investigations.

We have included a new figure in the manuscript, where we report the times taken by the various tools of MGS-Fast for metagenomic datasets ranging from 1.5GB to 11GB in file size, from an Illumina HiSeq 2000 microbiome dataset (https://www.ebi.ac.uk/ena/data/view/SRX978361). The times for MGS-Fast to complete the analysis ranged from 20 minutes for the smaller read sets, to 2.5 hours for the largest files. The cumulative time to process all the datasets was approximately 15 hours (900 minutes). Therefore, for five times larger WGS metagenomic studies (for example 100 samples), the complete study could be processed in the course of a few days. Furthermore, in order to remove any bias due to contention of hard disk or memory input / output (I/O), in our measurements we run a single dataset at a time on the server, both for MGS-Fast and the other pipelines it was compared to. In a production setting and depending on the available computational capacity, researchers could of course run multiple instances of MGS-Fast in parallel, further reducing the required time for completing the analysis of the data in such large-scale studies.

4. It is unclear what are the advantages of the approach compared to existing alternatives and how the computational performances of the proposed pipeline compares against existing alternatives.

Comparison of the computational performance with other pipelines for metagenomic reads annotation has been described in the earlier response to comment 2.

Regarding usability, we have leveraged Docker container technology, and bundled all the required software components and the MGS-Fast pipeline as a pre-configured, ready to execute bioinformatics package for performing standardized, automated metagenomics analysis on any desktop or laptop computer running Windows, MacOS or Linux. Besides the main pipeline for metagenomic annotation, MGS-Fast also includes data pre-processing, filtering, and quality control, along with all the required supporting data such as genome indexes and databases, within the Docker container. The container can be easily installed by non-bioinformatics experts with a single command, as described in our Suppl. manual and as result have a fully functional MGS-Fast. For the other metagenomic annotation pipelines included in our comparison, we found that we needed to spend significant amount of time to install all the dependencies for each compared pipeline to be fully functional. For example, HUMAnN2 requires users to install MetaPhlAn2, Bowtie2, Diamond and MinPath, and each of these tools requires to be retrieved from a separate software repository and has a different set of dependencies. From our experience during the process we found that the cumulative time and effort setting up all the dependencies, surpases the actually for setting up the intended pipeline, which can be difficult for laboratories who do not have bioinformatics engineers or software developers on staff. In addition, the other pipelines require users to perform quality control and other corrections such as read trimming, using external tools which means additional installation and configuration effort. Furthermore, besides software expertise for the tools installation, significant computational resources might be required as well during setup. For example, Kraken requires a server with at least 250GB of RAM memory and five or more hours of computation (as stated on the Kraken documentation (http://ccb.jhu.edu/software/kraken/MANUAL.html), in order to complete the building of the database used as genome annotation index. In our case for example, we had to utilize a university computer cluster to complete the database building for Kraken, while with MGS-Fast we offer pre-build indexes that are automatically downloaded during installation.

Overall, our target audience are laboratories that have minimal or no bioinformatics expertise, and with the lowering in costs of NGS technology they can now start generate metagenomic data but

need a user-friendly, accessible method for metagenomic annotation. Our approach with MGS-Fast is to provide an all inclusive, automatically deployable data analysis pipeline that comes pre-configured and ready to use within a Docker container. Upon installation, MGS-Fast provides direct access to the Galaxy interface, and users can run the pipeline without any prior bioinformatics or software development expertise. In this regard, MGS-Fast provides a unique advantage when compared to the other available approaches. Furthermore, beside the software usability aspect of MGS-Fast, we have added results and discussion in our manuscript regarding its metagenomic annotation capabilities, since we found that our results align closely with these from the original study of liver cirrhosis gut microbiome data by Qin et al. We have also added discussion regarding the precision of Metaphlan2 used in our pipeline, and how it enables us to provide researchers with precise metagenomic annotation, in a user friendly and accessible format through Galaxy interface of MGS-Fast.

With MGS-Fast we can contribute a novel tool to the metagenomics field, benefiting the "long-tail" of researchers running laboratories in smaller institutions, who want to incorporate metagenomics sequencing approaches in their research but do not have access to bioinformatics core facilities or the funds to hire professional bioinformaticians on staff. We believe that MGS-Fast can lower the data analysis barrier for such laboratories, and enable them to routinely integrate metagenomic sequencing technology as part of the laboratory standard research techniques.
