# Supplementary material for: MGS-Fast: Metagenomic shotgun data fast annotation using microbial gene catalogs
Source: Gigascience. 2019 Apr 3;8(4):giz020. doi: 10.1093/gigascience/giz020 (PMC6446249; doi:10.1093/gigascience/giz020)
Supplement: Supplement_Files.zip [file giz020_supplement_files.zip › KEGG modules - 502 KEGG IDs.docx]

**List of KEGG modules matching the 502 KEGG IDs found to have significantly different abundance scores (FDR > 0.05).**

[M00173 Reductive citrate cycle (Arnon-Buchanan cycle)](http://www.genome.jp/kegg-bin/show_module?1525556305102537/M00173.args) (5)

[M00439 Oligopeptide transport system](http://www.genome.jp/kegg-bin/show_module?1525556305102537/M00439.args) (5)

[M00550 Ascorbate degradation, ascorbate => D-xylulose-5P](http://www.genome.jp/kegg-bin/show_module?1525556305102537/M00550.args) (4)

[M00324 Dipeptide transport system](http://www.genome.jp/kegg-bin/show_module?1525556305102537/M00324.args) (4)

[M00529 Denitrification, nitrate => nitrogen](http://www.genome.jp/kegg-bin/show_module?1525556305102537/M00529.args) (4)

[M00034 Methionine salvage pathway](http://www.genome.jp/kegg-bin/show_module?1525556305102537/M00034.args) (4)

[M00307 Pyruvate oxidation, pyruvate => acetyl-CoA](http://www.genome.jp/kegg-bin/show_module?1525556305102537/M00307.args) (4)

[M00009 Citrate cycle (TCA cycle, Krebs cycle)](http://www.genome.jp/kegg-bin/show_module?1525556305102537/M00009.args) (4)

[M00319 Manganese/zinc/iron transport system](http://www.genome.jp/kegg-bin/show_module?1525556305102537/M00319.args) (4)

[M00270 PTS system, trehalose-specific II component](http://www.genome.jp/kegg-bin/show_module?1525556305102537/M00270.args) (4)

[M00228 Putative glutamine transport system](http://www.genome.jp/kegg-bin/show_module?1525556305102537/M00228.args) (3)

[M00360 Aminoacyl-tRNA biosynthesis, prokaryotes](http://www.genome.jp/kegg-bin/show_module?1525556305102537/M00360.args) (3)

[M00276 PTS system, mannose-specific II component](http://www.genome.jp/kegg-bin/show_module?1525556305102537/M00276.args) (3)

[M00806 PTS system, maltose-specific II component](http://www.genome.jp/kegg-bin/show_module?1525556305102537/M00806.args) (3)

[M00011 Citrate cycle, second carbon oxidation, 2-oxoglutarate => oxaloacetate](http://www.genome.jp/kegg-bin/show_module?1525556305102537/M00011.args) (3)

[M00582 Energy-coupling factor transport system](http://www.genome.jp/kegg-bin/show_module?1525556305102537/M00582.args) (3)

[M00064 ADP-L-glycero-D-manno-heptose biosynthesis](http://www.genome.jp/kegg-bin/show_module?1525556305102537/M00064.args) (3)

[M00115 NAD biosynthesis, aspartate => NAD](http://www.genome.jp/kegg-bin/show_module?1525556305102537/M00115.args) (3)

[M00271 PTS system, beta-glucoside-specific II component](http://www.genome.jp/kegg-bin/show_module?1525556305102537/M00271.args) (3)

[M00530 Dissimilatory nitrate reduction, nitrate => ammonia](http://www.genome.jp/kegg-bin/show_module?1525556305102537/M00530.args) (3)

[M00165 Reductive pentose phosphate cycle (Calvin cycle)](http://www.genome.jp/kegg-bin/show_module?1525556305102537/M00165.args) (3)

[M00725 Cationic antimicrobial peptide (CAMP) resistance, dltABCD operon](http://www.genome.jp/kegg-bin/show_module?1525556305102537/M00725.args) (3)

[M00003 Gluconeogenesis, oxaloacetate => fructose-6P](http://www.genome.jp/kegg-bin/show_module?1525556305102537/M00003.args) (3)

[M00121 Heme biosynthesis, glutamate => protoheme/siroheme](http://www.genome.jp/kegg-bin/show_module?1525556305102537/M00121.args) (3)

[M00269 PTS system, sucrose-specific II component](http://www.genome.jp/kegg-bin/show_module?1525556305102537/M00269.args) (3)

[M00283 PTS system, ascorbate-specific II component](http://www.genome.jp/kegg-bin/show_module?1525556305102537/M00283.args) (3)

[M00227 Glutamine transport system](http://www.genome.jp/kegg-bin/show_module?1525556305102537/M00227.args) (3)

[M00273 PTS system, fructose-specific II component](http://www.genome.jp/kegg-bin/show_module?1525556305102537/M00273.args) (3)

[M00429 Competence-related DNA transformation transporter](http://www.genome.jp/kegg-bin/show_module?1525556305102537/M00429.args) (3)

[M00238 D-Methionine transport system](http://www.genome.jp/kegg-bin/show_module?1525556305102537/M00238.args) (3)

[M00096 C5 isoprenoid biosynthesis, non-mevalonate pathway](http://www.genome.jp/kegg-bin/show_module?1525556305102537/M00096.args) (2)

[M00472 NarQ-NarP (nitrate respiration) two-component regulatory system](http://www.genome.jp/kegg-bin/show_module?1525556305102537/M00472.args) (2)

[M00026 Histidine biosynthesis, PRPP => histidine](http://www.genome.jp/kegg-bin/show_module?1525556305102537/M00026.args) (2)

[M00707 Multidrug resistance, MdlAB/SmdAB transporter](http://www.genome.jp/kegg-bin/show_module?1525556305102537/M00707.args) (2)

[M00025 Tyrosine biosynthesis, chorismate => tyrosine](http://www.genome.jp/kegg-bin/show_module?1525556305102537/M00025.args) (2)

[M00171 C4-dicarboxylic acid cycle, NAD - malic enzyme type](http://www.genome.jp/kegg-bin/show_module?1525556305102537/M00171.args) (2)

[M00361 Nucleotide sugar biosynthesis, eukaryotes](http://www.genome.jp/kegg-bin/show_module?1525556305102537/M00361.args) (2)

[M00699 Multidrug resistance, efflux pump AmeABC](http://www.genome.jp/kegg-bin/show_module?1525556305102537/M00699.args) (2)

[M00024 Phenylalanine biosynthesis, chorismate => phenylalanine](http://www.genome.jp/kegg-bin/show_module?1525556305102537/M00024.args) (2)

[M00209 Osmoprotectant transport system](http://www.genome.jp/kegg-bin/show_module?1525556305102537/M00209.args) (2)

[M00287 PTS system, galactosamine-specific II component](http://www.genome.jp/kegg-bin/show_module?1525556305102537/M00287.args) (2)

[M00647 Multidrug resistance, efflux pump AcrAB-TolC/SmeDEF](http://www.genome.jp/kegg-bin/show_module?1525556305102537/M00647.args) (2)

[M00331 Type II general secretion pathway](http://www.genome.jp/kegg-bin/show_module?1525556305102537/M00331.args) (2)

[M00172 C4-dicarboxylic acid cycle, NADP - malic enzyme type](http://www.genome.jp/kegg-bin/show_module?1525556305102537/M00172.args) (2)

[M00234 Cystine transport system](http://www.genome.jp/kegg-bin/show_module?1525556305102537/M00234.args) (2)

[M00022 Shikimate pathway, phosphoenolpyruvate + erythrose-4P => chorismate](http://www.genome.jp/kegg-bin/show_module?1525556305102537/M00022.args) (2)

[M00453 QseC-QseB (quorum sensing) two-component regulatory system](http://www.genome.jp/kegg-bin/show_module?1525556305102537/M00453.args) (2)

[M00116 Menaquinone biosynthesis, chorismate => menaquinone](http://www.genome.jp/kegg-bin/show_module?1525556305102537/M00116.args) (2)

[M00804 Complete nitrification, comammox, ammonia => nitrite => nitrate](http://www.genome.jp/kegg-bin/show_module?1525556305102537/M00804.args) (2)

[M00012 Glyoxylate cycle](http://www.genome.jp/kegg-bin/show_module?1525556305102537/M00012.args) (2)

[M00001 Glycolysis (Embden-Meyerhof pathway), glucose => pyruvate](http://www.genome.jp/kegg-bin/show_module?1525556305102537/M00001.args) (2)

[M00082 Fatty acid biosynthesis, initiation](http://www.genome.jp/kegg-bin/show_module?1525556305102537/M00082.args) (2)

[M00740 Methylaspartate cycle](http://www.genome.jp/kegg-bin/show_module?1525556305102537/M00740.args) (2)

[M00095 C5 isoprenoid biosynthesis, mevalonate pathway](http://www.genome.jp/kegg-bin/show_module?1525556305102537/M00095.args) (2)

[M00718 Multidrug resistance, efflux pump MexAB-OprM](http://www.genome.jp/kegg-bin/show_module?1525556305102537/M00718.args) (2)

[M00191 Thiamine transport system](http://www.genome.jp/kegg-bin/show_module?1525556305102537/M00191.args) (2)

[M00120 Coenzyme A biosynthesis, pantothenate => CoA](http://www.genome.jp/kegg-bin/show_module?1525556305102537/M00120.args) (2)

[M00167 Reductive pentose phosphate cycle, glyceraldehyde-3P => ribulose-5P](http://www.genome.jp/kegg-bin/show_module?1525556305102537/M00167.args) (2)

[M00728 Cationic antimicrobial peptide (CAMP) resistance, envelope protein folding and degrading factors DegP and DsbA](http://www.genome.jp/kegg-bin/show_module?1525556305102537/M00728.args) (2)

[M00002 Glycolysis, core module involving three-carbon compounds](http://www.genome.jp/kegg-bin/show_module?1525556305102537/M00002.args) (2)

[M00215 D-Xylose transport system](http://www.genome.jp/kegg-bin/show_module?1525556305102537/M00215.args) (2)

[M00089 Triacylglycerol biosynthesis](http://www.genome.jp/kegg-bin/show_module?1525556305102537/M00089.args) (1)

[M00378 F420 biosynthesis](http://www.genome.jp/kegg-bin/show_module?1525556305102537/M00378.args) (1)

[M00136 GABA biosynthesis, prokaryotes, putrescine => GABA](http://www.genome.jp/kegg-bin/show_module?1525556305102537/M00136.args) (1)

[M00357 Methanogenesis, acetate => methane](http://www.genome.jp/kegg-bin/show_module?1525556305102537/M00357.args) (1)

[M00346 Formaldehyde assimilation, serine pathway](http://www.genome.jp/kegg-bin/show_module?1525556305102537/M00346.args) (1)

[M00479 DesK-DesR (membrane lipid fluidity regulation) two-component regulatory system](http://www.genome.jp/kegg-bin/show_module?1525556305102537/M00479.args) (1)

[M00349 Microcin C transport system](http://www.genome.jp/kegg-bin/show_module?1525556305102537/M00349.args) (1)

[M00524 FixL-FixJ (nitrogen fixation) two-component regulatory system](http://www.genome.jp/kegg-bin/show_module?1525556305102537/M00524.args) (1)

[M00125 Riboflavin biosynthesis, GTP => riboflavin/FMN/FAD](http://www.genome.jp/kegg-bin/show_module?1525556305102537/M00125.args) (1)

[M00569 Catechol meta-cleavage, catechol => acetyl-CoA / 4-methylcatechol => propanoyl-CoA](http://www.genome.jp/kegg-bin/show_module?1525556305102537/M00569.args) (1)

[M00564 Helicobacter pylori pathogenicity signature, cagA pathogenicity island](http://www.genome.jp/kegg-bin/show_module?1525556305102537/M00564.args) (1)

[M00168 CAM (Crassulacean acid metabolism), dark](http://www.genome.jp/kegg-bin/show_module?1525556305102537/M00168.args) (1)

[M00525 Lysine biosynthesis, acetyl-DAP pathway, aspartate => lysine](http://www.genome.jp/kegg-bin/show_module?1525556305102537/M00525.args) (1)

[M00004 Pentose phosphate pathway (Pentose phosphate cycle)](http://www.genome.jp/kegg-bin/show_module?1525556305102537/M00004.args) (1)

[M00646 Multidrug resistance, efflux pump AcrAD-TolC](http://www.genome.jp/kegg-bin/show_module?1525556305102537/M00646.args) (1)

[M00317 Manganese/iron transport system](http://www.genome.jp/kegg-bin/show_module?1525556305102537/M00317.args) (1)

[M00129 Ascorbate biosynthesis, animals, glucose-1P => ascorbate](http://www.genome.jp/kegg-bin/show_module?1525556305102537/M00129.args) (1)

[M00027 GABA (gamma-Aminobutyrate) shunt](http://www.genome.jp/kegg-bin/show_module?1525556305102537/M00027.args) (1)

[M00726 Cationic antimicrobial peptide (CAMP) resistance, lysyl-phosphatidylglycerol (L-PG) synthase MprF](http://www.genome.jp/kegg-bin/show_module?1525556305102537/M00726.args) (1)

[M00377 Reductive acetyl-CoA pathway (Wood-Ljungdahl pathway)](http://www.genome.jp/kegg-bin/show_module?1525556305102537/M00377.args) (1)

[M00394 RNA degradosome](http://www.genome.jp/kegg-bin/show_module?1525556305102537/M00394.args) (1)

[M00362 Nucleotide sugar biosynthesis, prokaryotes](http://www.genome.jp/kegg-bin/show_module?1525556305102537/M00362.args) (1)

[M00566 Dipeptide transport system, Firmicutes](http://www.genome.jp/kegg-bin/show_module?1525556305102537/M00566.args) (1)

[M00122 Cobalamin biosynthesis, cobinamide => cobalamin](http://www.genome.jp/kegg-bin/show_module?1525556305102537/M00122.args) (1)

[M00456 ArcB-ArcA (anoxic redox control) two-component regulatory system](http://www.genome.jp/kegg-bin/show_module?1525556305102537/M00456.args) (1)

[M00308 Semi-phosphorylative Entner-Doudoroff pathway, gluconate => glycerate-3P](http://www.genome.jp/kegg-bin/show_module?1525556305102537/M00308.args) (1)

[M00035 Methionine degradation](http://www.genome.jp/kegg-bin/show_module?1525556305102537/M00035.args) (1)

[M00579 Phosphate acetyltransferase-acetate kinase pathway, acetyl-CoA => acetate](http://www.genome.jp/kegg-bin/show_module?1525556305102537/M00579.args) (1)

[M00290 Holo-TFIIH complex](http://www.genome.jp/kegg-bin/show_module?1525556305102537/M00290.args) (1)

[M00743 Aminoglycoside resistance, protease HtpX](http://www.genome.jp/kegg-bin/show_module?1525556305102537/M00743.args) (1)

[M00521 CiaH-CiaR two-component regulatory system](http://www.genome.jp/kegg-bin/show_module?1525556305102537/M00521.args) (1)

[M00366 C10-C20 isoprenoid biosynthesis, plants](http://www.genome.jp/kegg-bin/show_module?1525556305102537/M00366.args) (1)

[M00374 Dicarboxylate-hydroxybutyrate cycle](http://www.genome.jp/kegg-bin/show_module?1525556305102537/M00374.args) (1)

[M00192 Putative thiamine transport system](http://www.genome.jp/kegg-bin/show_module?1525556305102537/M00192.args) (1)

[M00113 Jasmonic acid biosynthesis](http://www.genome.jp/kegg-bin/show_module?1525556305102537/M00113.args) (1)

[M00454 KdpD-KdpE (potassium transport) two-component regulatory system](http://www.genome.jp/kegg-bin/show_module?1525556305102537/M00454.args) (1)

[M00166 Reductive pentose phosphate cycle, ribulose-5P => glyceraldehyde-3P](http://www.genome.jp/kegg-bin/show_module?1525556305102537/M00166.args) (1)

[M00268 PTS system, alpha-glucoside-specific II component](http://www.genome.jp/kegg-bin/show_module?1525556305102537/M00268.args) (1)

[M00741 Propanoyl-CoA metabolism, propanoyl-CoA => succinyl-CoA](http://www.genome.jp/kegg-bin/show_module?1525556305102537/M00741.args) (1)

[M00505 KinB-AlgB (alginate production) two-component regulatory system](http://www.genome.jp/kegg-bin/show_module?1525556305102537/M00505.args) (1)

[M00266 PTS system, maltose/glucose-specific II component](http://www.genome.jp/kegg-bin/show_module?1525556305102537/M00266.args) (1)

[M00169 CAM (Crassulacean acid metabolism), light](http://www.genome.jp/kegg-bin/show_module?1525556305102537/M00169.args) (1)

[M00303 PTS system, N-acetylmuramic acid-specific II component](http://www.genome.jp/kegg-bin/show_module?1525556305102537/M00303.args) (1)

[M00183 RNA polymerase, bacteria](http://www.genome.jp/kegg-bin/show_module?1525556305102537/M00183.args) (1)

[M00170 C4-dicarboxylic acid cycle, phosphoenolpyruvate carboxykinase type](http://www.genome.jp/kegg-bin/show_module?1525556305102537/M00170.args) (1)

[M00083 Fatty acid biosynthesis, elongation](http://www.genome.jp/kegg-bin/show_module?1525556305102537/M00083.args) (1)

[M00506 CheA-CheYBV (chemotaxis) two-component regulatory system](http://www.genome.jp/kegg-bin/show_module?1525556305102537/M00506.args) (1)

[M00580 Pentose phosphate pathway, archaea, fructose 6P => ribose 5P](http://www.genome.jp/kegg-bin/show_module?1525556305102537/M00580.args) (1)

[M00178 Ribosome, bacteria](http://www.genome.jp/kegg-bin/show_module?1525556305102537/M00178.args) (1)

[M00491 arabinogalactan oligomer/maltooligosaccharide transport system](http://www.genome.jp/kegg-bin/show_module?1525556305102537/M00491.args) (1)

[M00040 Tyrosine biosynthesis, prephanate => pretyrosine => tyrosine](http://www.genome.jp/kegg-bin/show_module?1525556305102537/M00040.args) (1)

[M00251 Teichoic acid transport system](http://www.genome.jp/kegg-bin/show_module?1525556305102537/M00251.args) (1)

[M00211 Putative ABC transport system](http://www.genome.jp/kegg-bin/show_module?1525556305102537/M00211.args) (1)

[M00364 C10-C20 isoprenoid biosynthesis, bacteria](http://www.genome.jp/kegg-bin/show_module?1525556305102537/M00364.args) (1)

[M00010 Citrate cycle, first carbon oxidation, oxaloacetate => 2-oxoglutarate](http://www.genome.jp/kegg-bin/show_module?1525556305102537/M00010.args) (1)

[M00447 CpxA-CpxR (envelope stress response) two-component regulatory system](http://www.genome.jp/kegg-bin/show_module?1525556305102537/M00447.args) (1)

[M00468 SaeS-SaeR (staphylococcal virulence regulation) two-component regulatory system](http://www.genome.jp/kegg-bin/show_module?1525556305102537/M00468.args) (1)

[M00495 AgrC-AgrA (exoprotein synthesis) two-component regulatory system](http://www.genome.jp/kegg-bin/show_module?1525556305102537/M00495.args) (1)

[M00023 Tryptophan biosynthesis, chorismate => tryptophan](http://www.genome.jp/kegg-bin/show_module?1525556305102537/M00023.args) (1)

[M00615 Nitrate assimilation](http://www.genome.jp/kegg-bin/show_module?1525556305102537/M00615.args) (1)

[M00631 D-Galacturonate degradation (bacteria), D-galacturonate => pyruvate + D-glyceraldehyde 3P](http://www.genome.jp/kegg-bin/show_module?1525556305102537/M00631.args) (1)

[M00189 Molybdate transport system](http://www.genome.jp/kegg-bin/show_module?1525556305102537/M00189.args) (1)

[M00709 Macrolide resistance, MacAB-TolC transporter](http://www.genome.jp/kegg-bin/show_module?1525556305102537/M00709.args) (1)

[M00118 Glutathione biosynthesis, glutamate => glutathione](http://www.genome.jp/kegg-bin/show_module?1525556305102537/M00118.args) (1)

[M00727 Cationic antimicrobial peptide (CAMP) resistance, N-acetylmuramoyl-L-alanine amidase AmiA and AmiC](http://www.genome.jp/kegg-bin/show_module?1525556305102537/M00727.args) (1)

[M00018 Threonine biosynthesis, aspartate => homoserine => threonine](http://www.genome.jp/kegg-bin/show_module?1525556305102537/M00018.args) (1)

[M00541 Benzoyl-CoA degradation, benzoyl-CoA => 3-hydroxypimeloyl-CoA](http://www.genome.jp/kegg-bin/show_module?1525556305102537/M00541.args) (1)

[M00044 Tyrosine degradation, tyrosine => homogentisate](http://www.genome.jp/kegg-bin/show_module?1525556305102537/M00044.args) (1)

[M00334 Type VI secretion system](http://www.genome.jp/kegg-bin/show_module?1525556305102537/M00334.args) (1)

[M00531 Assimilatory nitrate reduction, nitrate => ammonia](http://www.genome.jp/kegg-bin/show_module?1525556305102537/M00531.args) (1)

[M00048 Inosine monophosphate biosynthesis, PRPP + glutamine => IMP](http://www.genome.jp/kegg-bin/show_module?1525556305102537/M00048.args) (1)

[M00133 Polyamine biosynthesis, arginine => agmatine => putrescine => spermidine](http://www.genome.jp/kegg-bin/show_module?1525556305102537/M00133.args) (1)

[M00552 D-galactonate degradation, De Ley-Doudoroff pathway, D-galactonate => glycerate-3P](http://www.genome.jp/kegg-bin/show_module?1525556305102537/M00552.args) (1)

[M00021 Cysteine biosynthesis, serine => cysteine](http://www.genome.jp/kegg-bin/show_module?1525556305102537/M00021.args) (1)

[M00007 Pentose phosphate pathway, non-oxidative phase, fructose 6P => ribose 5P](http://www.genome.jp/kegg-bin/show_module?1525556305102537/M00007.args) (1)

[M00080 Lipopolysaccharide biosynthesis, inner core => outer core => O-antigen](http://www.genome.jp/kegg-bin/show_module?1525556305102537/M00080.args) (1)

[M00246 Nickel transport system](http://www.genome.jp/kegg-bin/show_module?1525556305102537/M00246.args) (1)

[M00440 Nickel transport system](http://www.genome.jp/kegg-bin/show_module?1525556305102537/M00440.args) (1)

[M00060 Lipopolysaccharide biosynthesis, KDO2-lipid A](http://www.genome.jp/kegg-bin/show_module?1525556305102537/M00060.args) (1)

[M00335 Sec (secretion) system](http://www.genome.jp/kegg-bin/show_module?1525556305102537/M00335.args) (1)

[M00272 PTS system, beta-glucoside (arbutin/salicin/cellobiose)-specific II component](http://www.genome.jp/kegg-bin/show_module?1525556305102537/M00272.args) (1)

[M00081 Pectin degradation](http://www.genome.jp/kegg-bin/show_module?1525556305102537/M00081.args) (1)

[M00532 Photorespiration](http://www.genome.jp/kegg-bin/show_module?1525556305102537/M00532.args) (1)

[M00577 Biotin biosynthesis, BioW pathway, pimelate => pimeloyl-CoA => biotin](http://www.genome.jp/kegg-bin/show_module?1525556305102537/M00577.args) (1)

[M00365 C10-C20 isoprenoid biosynthesis, archaea](http://www.genome.jp/kegg-bin/show_module?1525556305102537/M00365.args) (1)

[M00338 Cysteine biosynthesis, homocysteine + serine => cysteine](http://www.genome.jp/kegg-bin/show_module?1525556305102537/M00338.args) (1)

[M00134 Polyamine biosynthesis, arginine => ornithine => putrescine](http://www.genome.jp/kegg-bin/show_module?1525556305102537/M00134.args) (1)

[M00648 Multidrug resistance, efflux pump MdtABC](http://www.genome.jp/kegg-bin/show_module?1525556305102537/M00648.args) (1)

[M00436 Sulfonate transport system](http://www.genome.jp/kegg-bin/show_module?1525556305102537/M00436.args) (1)

[M00367 C10-C20 isoprenoid biosynthesis, non-plant eukaryotes](http://www.genome.jp/kegg-bin/show_module?1525556305102537/M00367.args) (1)

[M00376 3-Hydroxypropionate bi-cycle](http://www.genome.jp/kegg-bin/show_module?1525556305102537/M00376.args) (1)

[M00013 Malonate semialdehyde pathway, propanoyl-CoA => acetyl-CoA](http://www.genome.jp/kegg-bin/show_module?1525556305102537/M00013.args) (1)

[M00061 D-Glucuronate degradation, D-glucuronate => pyruvate + D-glyceraldehyde 3P](http://www.genome.jp/kegg-bin/show_module?1525556305102537/M00061.args) (1)

[M00017 Methionine biosynthesis, apartate => homoserine => methionine](http://www.genome.jp/kegg-bin/show_module?1525556305102537/M00017.args) (1)

[M00051 Uridine monophosphate biosynthesis, glutamine (+ PRPP) => UMP](http://www.genome.jp/kegg-bin/show_module?1525556305102537/M00051.args) (1)

[M00274 PTS system, mannitol-specific II component](http://www.genome.jp/kegg-bin/show_module?1525556305102537/M00274.args) (1)

[M00265 PTS system, glucose-specific II component](http://www.genome.jp/kegg-bin/show_module?1525556305102537/M00265.args) (1)

[M00126 Tetrahydrofolate biosynthesis, GTP => THF](http://www.genome.jp/kegg-bin/show_module?1525556305102537/M00126.args) (1)

[M00087 beta-Oxidation](http://www.genome.jp/kegg-bin/show_module?1525556305102537/M00087.args) (1)

[M00839 PdtaS-PdtaR two-component regulatory system](http://www.genome.jp/kegg-bin/show_module?1525556305102537/M00839.args) (1)

[M00549 Nucleotide sugar biosynthesis, glucose => UDP-glucose](http://www.genome.jp/kegg-bin/show_module?1525556305102537/M00549.args) (1)
