# Supplementary material for: MGS-Fast: Metagenomic shotgun data fast annotation using microbial gene catalogs
Source: Gigascience. 2019 Apr 3;8(4):giz020. doi: 10.1093/gigascience/giz020 (PMC6446249; doi:10.1093/gigascience/giz020)
Supplement: Supplement_Files.zip [file giz020_supplement_files.zip › V2 Suppl-Software-Manual.docx]

Fast functional annotation of metagenomic shotgun data by DNA alignment to a microbial gene catalog

**SUPPLEMENTARY USER MANUAL**

____________________________________________________________________________

____________________________________________________________________________

Table Of Contents

[Step 1: Downloading Our Package](#_1fob9te)

[Step 2: Setting Up Your Data](#_3znysh7)

[Step 3: Downloading Docker](#_2et92p0)

[Step 4: Accessing Your CLI and Logging Into Docker](#_tyjcwt)

[Step 4a: Terminal on MacOS/Linux](#_3dy6vkm)

[Step 4b: Command Prompt or PowerShell on Windows](#_1t3h5sf)

[Step 4c: Docker Login](#_4d34og8)

[Step 5: Pulling and Running the Docker Image With Data](#_3rdcrjn)

[Step 5a: Pulling the Image](#_26in1rg)

[Step 5b: Making Data Accessible (Windows Only)](#_lnxbz9)

[Step 5c: Running the Image and Mounting your Data](#_35nkun2)

[Step 6: Accessing and Logging into Galaxy](#_1ksv4uv)

[Step 6a: Accessing Galaxy via Web Browser](#_44sinio)

[Step 6b: Logging into the User Account](#_2jxsxqh)

[Step 7: Running The Workflow](#_z337ya)

[Step 7a: Switching Histories](#_3j2qqm3)

[Step 7b: Setting your Inputs and Running the Workflow](#_1y810tw)

[Step 8: Viewing and Downloading Your Results](#_4i7ojhp)

[Step 8a: Viewing your Results](#_2xcytpi)

[Step 8b: Downloading your Results](#_1ci93xb)

Creating a custom genome index and importing data [in Galaxy](#_3whwml4)

Details of tools and parameters used in the MGS-Fast workflow.

[Troubleshooting](#_2bn6wsx)

[Appendix](#_qsh70q)

[About Docker](#_3as4poj)

[About Galaxy](#_1pxezwc)

# Step 1: Downloading Our Package

We have hosted a package on our server that contains several items for your workflow. We understand that the package is quite large^^[[1]](#footnote-0)^^ so you can keep it for downloading and move to [Step 3: Downloading Docker](#_2et92p0)) so that you can move forward with the instructions and come back when this step is complete. You can download it from the link listed below. It is a large file of 17GB, therefore it will take some time to download based on your network connection speed:

<http://146.95.173.35:9988/metagenomics_package.tar.gz>

Simply click the link and your download will begin. A window will pop up for you to choose where to download it-- this image is taken on a MacOS machine, but works the same on all systems:


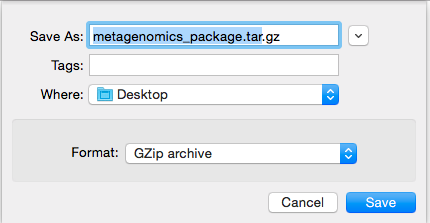


Downloading it on your Desktop is usually the easiest, so that everything can be viewed easily. For the rest of this text, we will have examples showing the package on your Desktop, however you can place the package wherever you feel comfortable.

The package contains the following items:

- 1. Human reference genome GRCh38 (hg38) in fasta format
  2. [P](http://hgdownload.cse.ucsc.edu/goldenPath/hg38/bigZips/hg38.fa.gz)re-built Bowtie2 indiceso[f](http://hgdownload.cse.ucsc.edu/goldenPath/hg38/bigZips/hg38.fa.gz) human reference genome GRCh38 (hg38)
  3. IGC catalog of microbial reference genomes from the Beijing Genome Institute
  4. Pre-built Bowtie2 indices of IGC catalog of microbial reference genomes
  5. Sample paired-end raw datasets in fastq format

# Step 2: Setting Up Your Data

Once downloaded, you can decompress the package by simply double clicking on it. Your computer will start extracting the data inside the package into a folder called ‘Metagenomics_Package’ on your Desktop (or the folder you downloaded your package.) You must decompress everything such that there are no files or folders with .tar or .gz suffixes.

In this ‘Metagenomics_Package’ folder, you can drag and drop any raw paired-end data that you may have to test. If you do not have any raw data and you are just trying out our workflow, there are sample datasets included in the package that can be used for testing.

After dragging and dropping your raw data (optional), your data structure should be similar to this:

Desktop [or desired filepath]/

|
 |----------- /Metagenomics_Package/
 |
 | ----------- **IGC**/

|

|----------- IGC.fa

|----------- IGC.1.bt2l

|----------- IGC.2.bt2l

|----------- IGC.3.bt2l

|----------- IGC.4.bt2l

|----------- IGC.rev.1.bt2l

|----------- IGC.rev.2.bt2l

| ----------- **hg38**/

|----------- hg38.fa

|----------- hg38.1.bt2

|----------- hg38.2.bt2

|----------- hg38.3.bt2

|----------- hg38.4.bt2

|----------- hg38.rev.1.bt2

|----------- hg38.rev.2.bt2

| ----------- **Sample_Datasets**/

|----------- SRR1582754_1.fastq

|----------- SRR1582754_2.fastq

| ----------- raw_data_1.fastq [optional, and any name with .fastq]

| ----------- raw_data_2.fastq [optional, and any name with .fastq]

# Step 3: Downloading Docker

Firstly, setup a Docker Hub account at:

<https://hub.docker.com/>

If you have a Docker account already, you can skip this step. This account will be necessary later on in the manual.

You can download the Docker engine from the Docker main page. Use this link and scroll down to select your operating system:

<https://www.docker.com/>


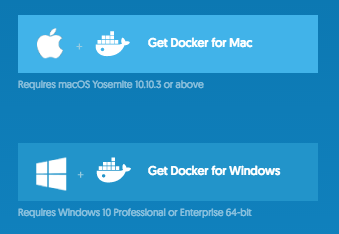


Download Docker for your operating system (Windows^^[[2]](#footnote-1)^^, MAC, Linux or Cloud) and be sure to download the **stable** release, not Beta. For further information about Docker installation (and installation via command line and Linux operating systems), you can visit this link:

<https://docs.docker.com/engine/installation/>

Once your Docker installer is downloaded, follow the on-screen instructions to complete the installation.

# Step 4: Accessing Your CLI and Logging Into Docker

After successful installation of the docker engine in your computer environment, you will need to access Docker via a Command Line Interface (CLI). In Linux and MacOS operating systems, the CLI is accessed via an application called Terminal, in which you can access Docker. On Windows operating systems, the CLI is available on the Command Prompt and on Windows 10 and above systems, it is also available through a program called Windows PowerShell (which is a powerful tool that accepts Unix commands as well.)

We will demonstrate how to access the Command Line Interface on each of these systems. If you already know how to do this, you can proceed to [Step 4c: Docker Login](#_4d34og8).

## Step 4a: Terminal on MacOS/Linux

You can quickly access your computer’s Terminal by searching for it. On MacOS, there is a search called Spotlight Search, which can be accessed by clicking on the magnifying glass on the top right corner in the Toolbar. In Linux systems, the Search bar is accessed via the applications drawer. In both cases you can simply type in Terminal and click on the application.

We have a demonstration video for both MacOS and Linux for reference:

**MacOS Environment:** <https://www.youtube.com/watch?v=0nidMS8kc04>

**Linux Environment**: <https://www.youtube.com/watch?v=72KieyXey28&feature=youtu.be>

## Step 4b: Command Prompt or PowerShell on Windows

On Windows, the Docker software can be accessed via both the Command prompt and the Windows PowerShell that contains the Command Line Interface. To operate and interact with Docker, open one of these programs by typing the name into the Search bar and pressing enter. During the course of this manual, we will be using the Windows PowerShell as an example.

**Windows Environment**: <https://www.youtube.com/watch?v=SUt_xUQjDoU>

## Step 4c: Docker Login

Once you are facing the CLI blank screen, you are ready to begin retrieving the MGS-Fast pipeline. Although the CLI screen is quite daunting, with a few commands you will have your Galaxy interface ready to go and you will be able to start performing your analyses.

To begin, we need to log into Docker using the account you created earlier in [Step 3: Downloading Docker](#_2et92p0). To do this, type the following command to login to your docker hub account on your respective Operating System’s CLI:

**>> docker login**

This will prompt you to login with your Docker username and password as such:


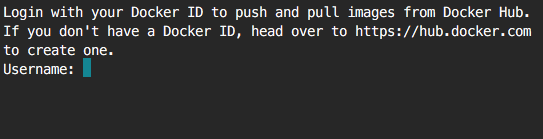


Once you are logged in, you have activated Docker in your environment and you can begin retrieving the Docker image for our pipeline.

#

#

# Step 5: Pulling and Running the Docker Image With Data

Docker^^[[3]](#footnote-2)^^ functions by having a computer environment stored in files called “images” or “containers.” We have our bioinformatics pipeline stored in one such container so the user can simply “pull” the image from the Docker Hub and “run” it to activate it on their own computer via a platform called Galaxy.^^[[4]](#footnote-3)^^ ^^[[5]](#footnote-4)^^

## Step 5a: Pulling the Image

Now we can pull the image for the Metagenomics Pipeline. To do this, type this command on your Command Line Interface after logging into your Docker account:

**>> docker pull bcil/metagenome:nyu_3.0**

You should see the “nyu_3.0” image from our bcil/metagenome repository start getting pulled. You can view the progress on your screen, where you can see each layer being downloaded on your local computer. Once the image has been complete, your screen should look similar to this:


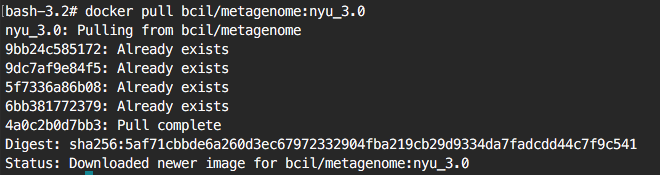


To test that your docker image has been downloaded correctly, type this command into your CLI:

**>> docker images**

This command tells you which images downloaded on your local computer. If everything went well, your screen should look like this (your image ID will be different):


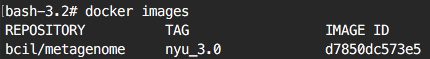


## Step 5b: Making Data Accessible (Windows Only)

If you are running the pipeline from a MacOS or Linux machine, you can skip forward to the [Step 5c: Running the Image and Mounting your Data](#_35nkun2). If you are running it from a Windows machine, it is important that you run through the following steps in order to make your data accessible to your virtual container on Docker. You must make your C: drive accessible through the Docker for Windows settings. You can access the settings through the Docker icon on the system tray drawer:


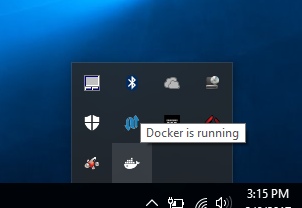


Simply right-click on the Docker icon and click on Settings...:


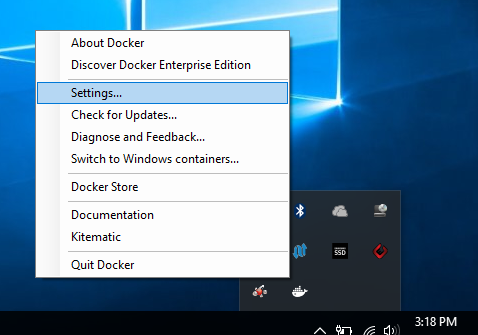


From the settings window, you can click on “Shared Drives” on the right pane and you will see a list of drives on your computer. Be sure to check the C drive in order to allow Docker to access any data on it. This allows us to mount our package (if it is on the C: drive).


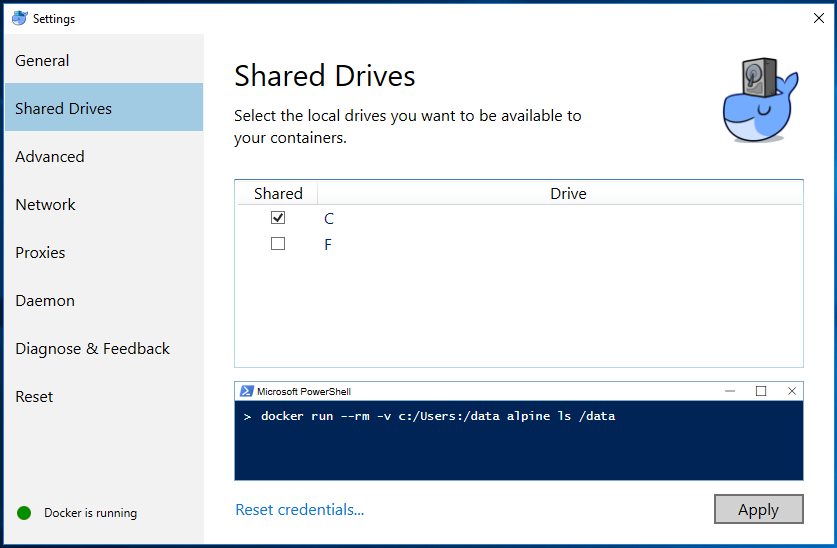


Be sure to click “Apply” and then type your Windows login password if Docker prompts you to sign in using your Windows user account. Now you are ready to run the image and mount your data.

## Step 5c: Running the Image and Mounting your Data

In order to run the workflow, you need to run the container that you pulled and also mount the Metagenomics_Package data folder into that container. Fortunately, you can do this in one line using Docker. In order to run the image, mount your data into the container, and broadcast Galaxy on your local computer, run this command from your Command Line Interface:

**>> docker run -v [Package File Location]:/home/data -tip 0.0.0.0:9020:8090 bcil/metagenome:nyu_5.0 /bin/bash**

Be sure to type the command identical to the way it is written above, except you must write your own full path location of the Metagenomics Package (the next page contains examples of how to do this). This command runs the image bcil/metagenome:nyu_3.0, specifies the path of the Metagenomics_Package and mounts it on the container to the /home/data path, and broadcasts the Galaxy workflow of the MGS-Fast pipeline inside the container on the local computer through port 9020.

The full Metagenomics_Package path must be used to mount the package onto the container. For users that may not be familiar with how to get the full path, here are examples of the full path of the package on the Desktop of different operating systems:

**MacOS:** /Users/[username]/Desktop/Metagenomics_Package

**Linux:** /home/[username]/Desktop/Metagenomics_Package

**Windows:** C:\Users\[username]\Desktop\Metagenomics_Package

Windows users must be wary that their local file paths contain backslashes instead of forward slashes, but the /home/data path must still contain forward slashes.

For a demonstration video of how to run the container and mount the data click on the link below. Included in the video is also a quick method of retrieving the file path of the package by simply dragging and dropping the folder into the CLI:

<http://www.youtube.com/watch?v=uB5KbRwT1rc>

Advanced users, if you are running your container remotely, you can broadcast to the remote host IP address instead of 127.0.0.1 (local host.) Additionally, you can change the 9020 to any port you would like, but you cannot change the 8090 port. For reference, I will be using port 9020 for the rest of the instructions.

The container will run, and the Galaxy interface will automatically start. After starting Galaxy, your files will be mounted onto the interface as well. If you followed all the directions correctly (and in this case you do not have your own data), you should see the following screen:


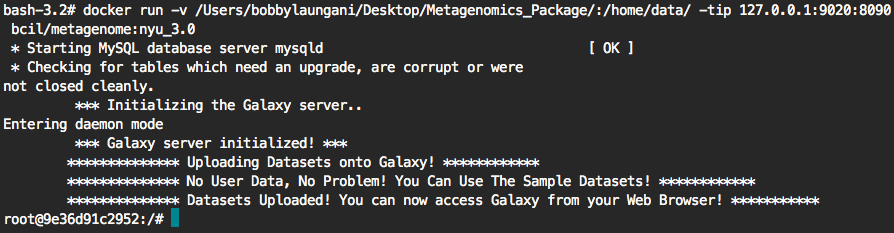


Once you have received the message that “You can now access Galaxy from your Web Browser!” then you can proceed to [Step 6: Accessing and Logging into Galaxy](#_1ksv4uv)

# Step 6: Accessing and Logging into Galaxy

Galaxy is a user-friendly web platform used to perform data-intensive biomedical research that is easily accessible to the research community.^^[[6]](#footnote-5)^^ We have implemented Galaxy to perform the bioinformatics analysis of the MGS-Fast pipeline in order to provide an easy-to-use workspace.

## Step 6a: Accessing Galaxy via Web Browser

To access the web interface of Galaxy that exists inside your virtual Docker container, open your favorite browser and open the following URL:

<http://127.0.0.1:9020>

Where 127.0.0.1 is the IP address of your own local device from and port 9020 is the front interface of your virtual Docker machine that we had declared when we ran the image. For the advanced users who are working remotely, you can access it via the remote IP address and/or the port you specified when running the container.

If everything went correctly, your screen should look like this (we used the Google Chrome):


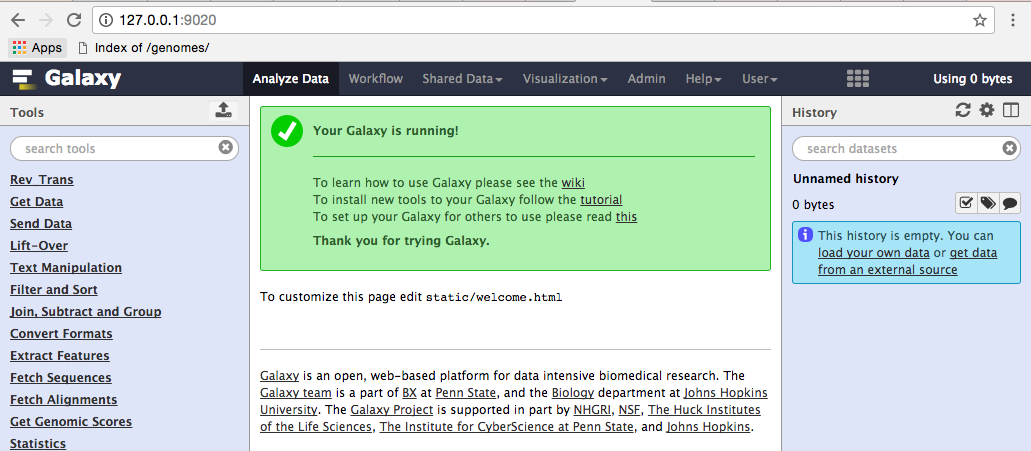


Here is a demonstration video on how to access Galaxy:

<https://www.youtube.com/watch?v=-sZmEDB_Mak>

## Step 6b: Logging into the User Account

To run the MGS-Fast workflow to perform metagenomics analyses on Galaxy, you must be logged into the **user@galaxy.edu** account on the interface. In order to be sure you are logged in, you can click on the User tab on the Galaxy toolbar and see if the screen looks like this:


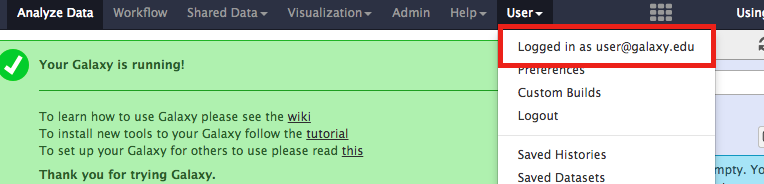


As you can see, in the user tab the first option says “Logged in as user@galaxy.edu.” If you do not have this option, you should login with the following credentials:

**Username / Email Address:** user@galaxy.edu

**Password:** galaxyuser

Logging in is necessary in order to save your work if need be and also to access the MGS-Fast workflow that we have created. The Metagenomics Package data also should have been successfully uploaded into this account. You can be sure that you have logged in correctly if you click on the User tab and match your screen to the image above. Once you are correctly logged in, you can proceed to running the workflow.

# Step 7: Running The Workflow

Once you have access to the Galaxy interface and are logged into the **user@galaxy.edu** user account, you are ready to run the workflow. All your data is already injected into the Galaxy interface and the histories are already created that contains your test data.

## Step 7a: Switching Histories

The first step is to switch to the history that contains the data that you will be running. To do this you will have to navigate to the right side of the Galaxy window, on the History pane. This contains a Wheel icon called “History options” in which you can view your saved histories.


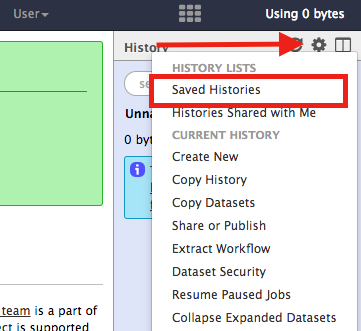


From the “Saved Histories” screen, you will notice that you have two histories created-- one called “Sample_Datasets” and one called “Metagenomics_Data.” The green box next to the histories indicate the number of datasets in the History. Since for this example I do not have any raw read data, I will switch to the Sample Data by simply clicking on the arrow next to the desired History and clicking “Switch”:


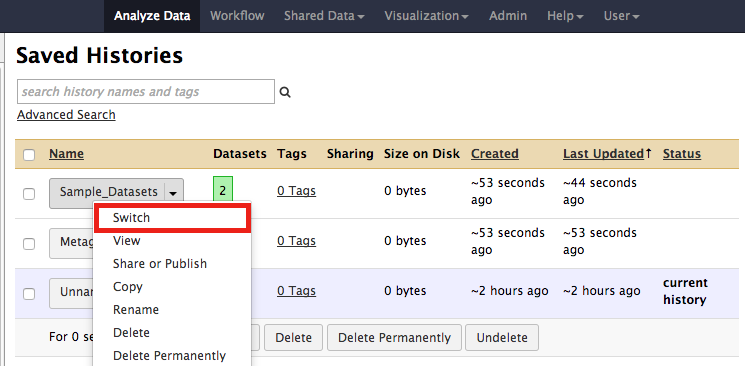


Once you click “Switch,” you will notice that the History pane will be updated and will reflect the data you selected. In my case again, the pane shows the Sample Datasets in the Metagenomics_Package. Your History pane should look the same or similar to this:


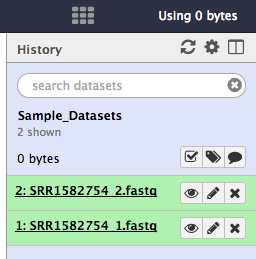


## Step 7b: Setting your Inputs and Running the Workflow

Now we are ready to run the pipeline on these datasets! In order to get started, simply click on the Workflow tab on the toolbar of Galaxy:


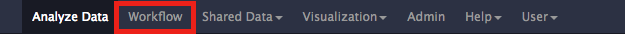


From the Workflows page, click on the arrow next to the MGS-Fast Pipeline and click Run:


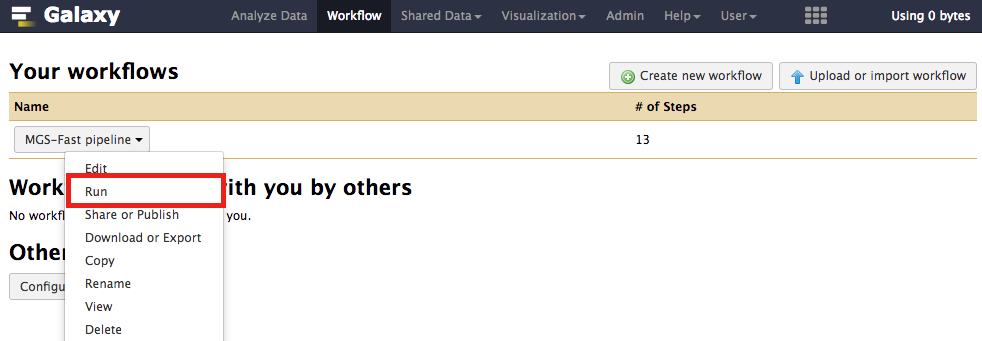


After clicking “Run,” you will see the entire workflow in a series of numbered steps on your screen. The first step is your input datasets, so make sure to select your paired end data for each input dataset. You will notice that the dataset options are from the your selected history. Also notice that there are two inputs so be sure to select one paired end sample per input:


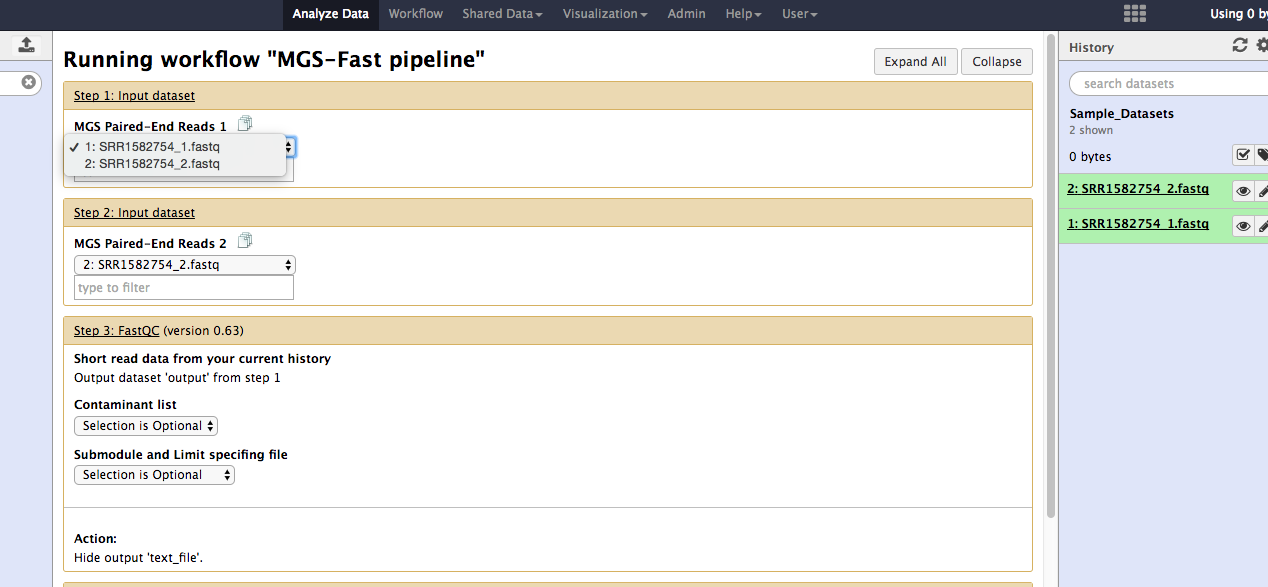


Once you have selected your inputs, scroll down to the bottom of the screen and check the box that says “Send Results to a New History.” This will run the workflow in a new History, and you can proceed to name it whatever you like (such as MGS-Fast Run 1):


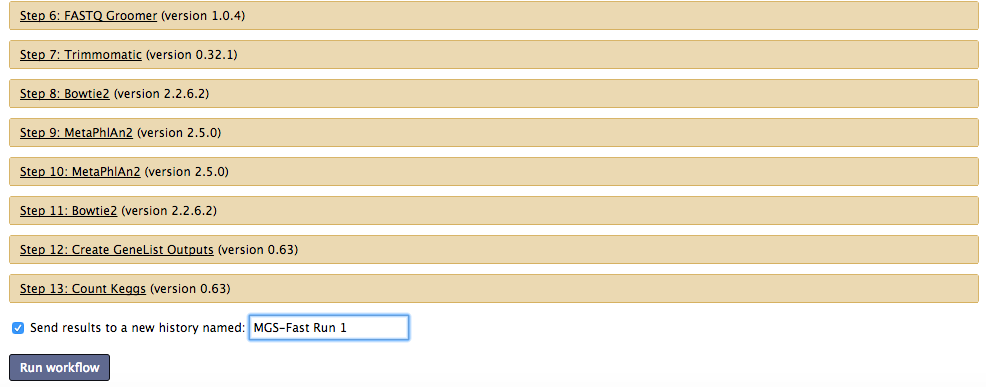


Once you are sure you have selected the correct input datasets and have chosen a good name for your run, click “Run Workflow”

This video shows how to run Docker MGS-Fast pipeline on Galaxy:

<https://www.youtube.com/watch?v=RNjntdYmuFs>

# Step 8: Viewing and Downloading Your Results

After clicking “Run Workflow,” your pipeline has officially begun. Since we sent our results to a new history, we can view the progress of our run by switching to that history. You can review how to do this by going back to [Step 7a: Switching Histories](#_3j2qqm3) and selecting the name of your History which you specified earlier. Once you switch to the history, you can see how far along each step is based on the color of the tool. If it is **Green**, it is complete; if it is **Yellow,** it is still in progress and if it is **Red**, you have approached some sort of error (for common errors, be sure to visit the [Troubleshooting](#_2bn6wsx) section.) While your pipeline is running, your screen should look something like this:


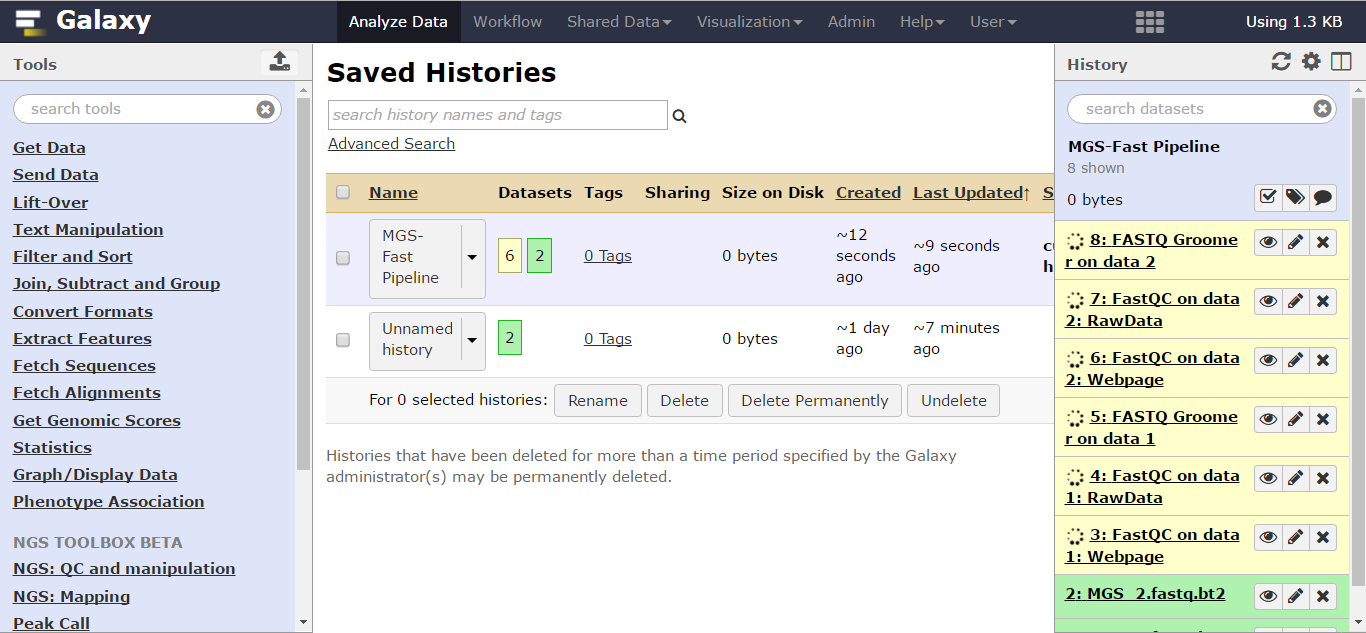


## Step 8a: Viewing your Results

You will know your run is complete when all of your tools in the pipeline turn green. Now you can view your results!

You can see a preview of each dataset and some details about each run by clicking on the name of each tool in the History pane. You can also see how large each dataset is, the format of the file, and see the first few lines from the dataset. On the right of each dataset, there are three icons:


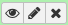


By clicking on the eye icon, you can view the dataset in the main pane of Galaxy, by clicking on the pencil icon, you can edit certain attributes of the dataset, and by clicking the cross icon you can delete the dataset.

Let’s view our results! Click on the eye icon for any dataset outputs generated from the tools in the pipeline on the History panel to view the results. Here is what a sample output from MetaPhlAn2 looks like:


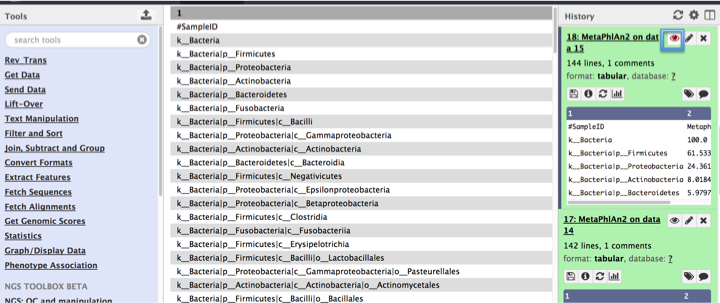


## Step 8b: Downloading your Results

You can even download the results from each tool to your local computer. When you click on the name of each tool, under the file details and above the dataset preview you will see the following four buttons:


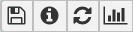


By clicking on the floppy disk drive icon, you can download your results. By clicking on the ‘i’ icon you can view the run details on the Galaxy main pane. By clicking on the “refresh” icon, you can run the tool again and configure the options. By clicking on the histogram, you can visualize your dataset into various kinds of data graphics.

Simply by clicking on the floppy disk drive icon, download any relevant results to your local computer. You can specify where you would like to save the file as well.

Congratulations! You have now completed your very first run of the MGS-Fast pipeline!

# Creating a custom genome index and importing data in Galaxy

There are two approaches through which users can create a Bowtie index for a custom genome of their choice. The first option is during the initial run of the MGS-Fast container, where a text based menu requests whether a location with a custom local genome will be provided (shown in the screenshots below), or the default will be downloaded from our data repository. Building the index with the custom genome is automatically handled by the scripts inside the container, and it will become available in the pipelines on the Galaxy interface without any effort by the user.

In the second option, even if the users have selected the default Bowtie2 index during the initial setup, they can at any point add custom indexes using our additional MGS-Fast pipeline in Galaxy, called “Custom Genome MGS-Fast”. This is identical with the workflow used for MGS-Fast, but with the option for users to provide a FASTA file containing the sequence(s) of the custom genome. The Bowtie2 index with the custom genome FASTA file is automatically build during the first run of the workflow, and then it will available for the users in subsequent runs of the customized workflow.

https://img.jgi.doe.gov/cgi-bin/m/main.cgi?section=TreeFile&page=domain&domain=all&domainfile=treenovpg94055_f603a60ef5a249490ea2d14f328a0c24&openfile=treestate94256_f603a60ef5a249490ea2d14f328a0c24&selectedfile=treestate94055_f603a60ef5a249490ea2d14f328a0c24&selectid=e126-&selectlevel=5#e126-

Furthermore, users can even create custom FASTA files for WGS metagenomes by creating collections of bacterial genomes through the NCBI web search interface, and send the collections to a single FASTA: https://support.ncbi.nlm.nih.gov/link/portal/28045/28049/Article/1928/How-do-I-download-sequence-records-from-the-web-in-the-Nucleotide-and-Protein-databases. An example containing custom sets of WGS genome for Bacteria and Archaea, which can be ownloaded as single file using the “Send To” function at the NCBI Entrez interface would be for example: https://www.ncbi.nlm.nih.gov/nuccore/?term=biomol_genomic%5BPROP%5D+%22bacteria%22+%5BOrganism%5D+OR+%22archaea%22%5BOrganism%5D The full documentation for the search parameters is available here https://www.ncbi.nlm.nih.gov/books/NBK49540/. Following the “Example” link on this tutorial and the “Send To FASTA” on the NCBI web interface, we were able to create a FASTA file with 30,000+ sequences through the NCBI web page. The downloaded FASTA will then given as an input to the workflow for building the custom genome as described above.


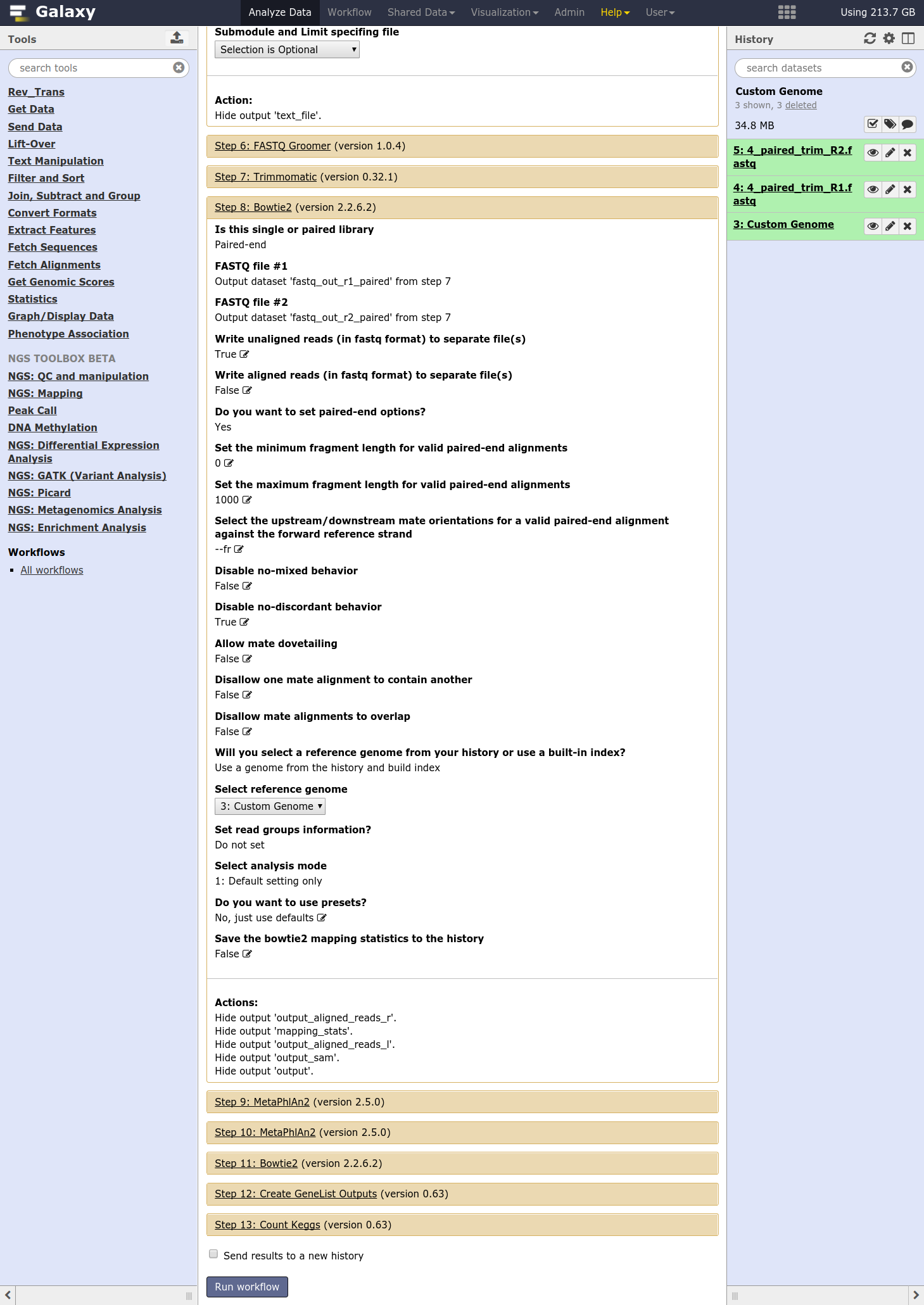


**Importing local data-sets into Galaxy for analysis with the MGS-Fast workflow.**

Following login into Galaxy, users can go to the “Admin” menu and select “Manage Data Libraries”, and then “Create new data library”:


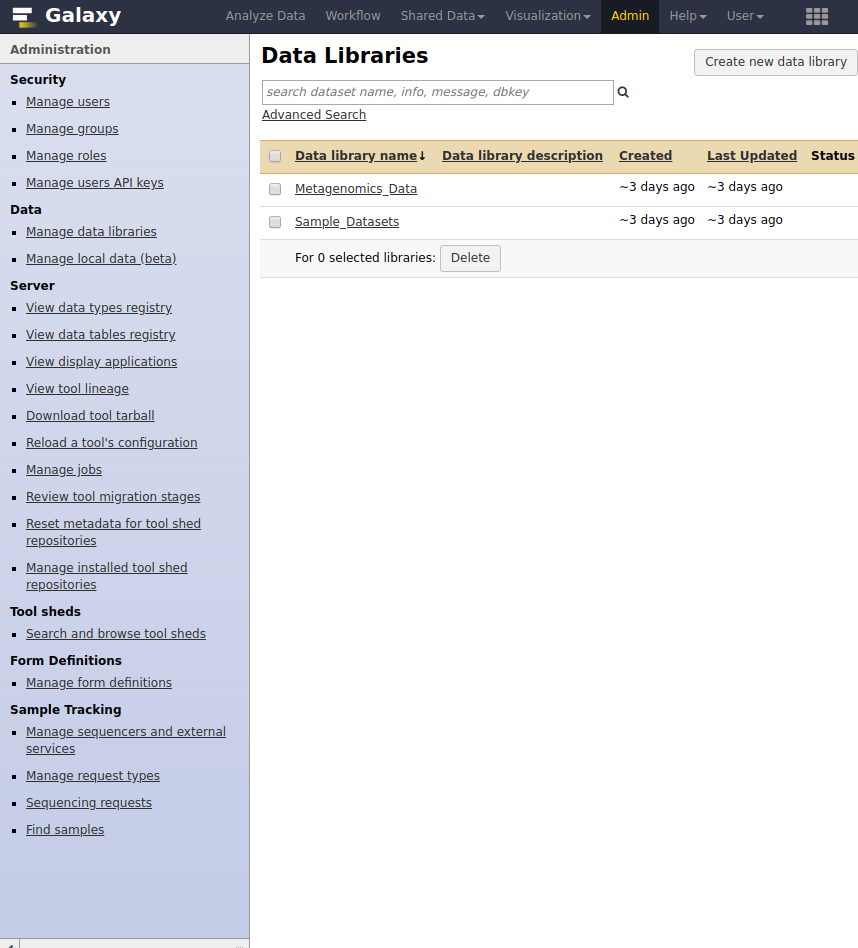


After specifying the name and description of the new data library and click “Create”:


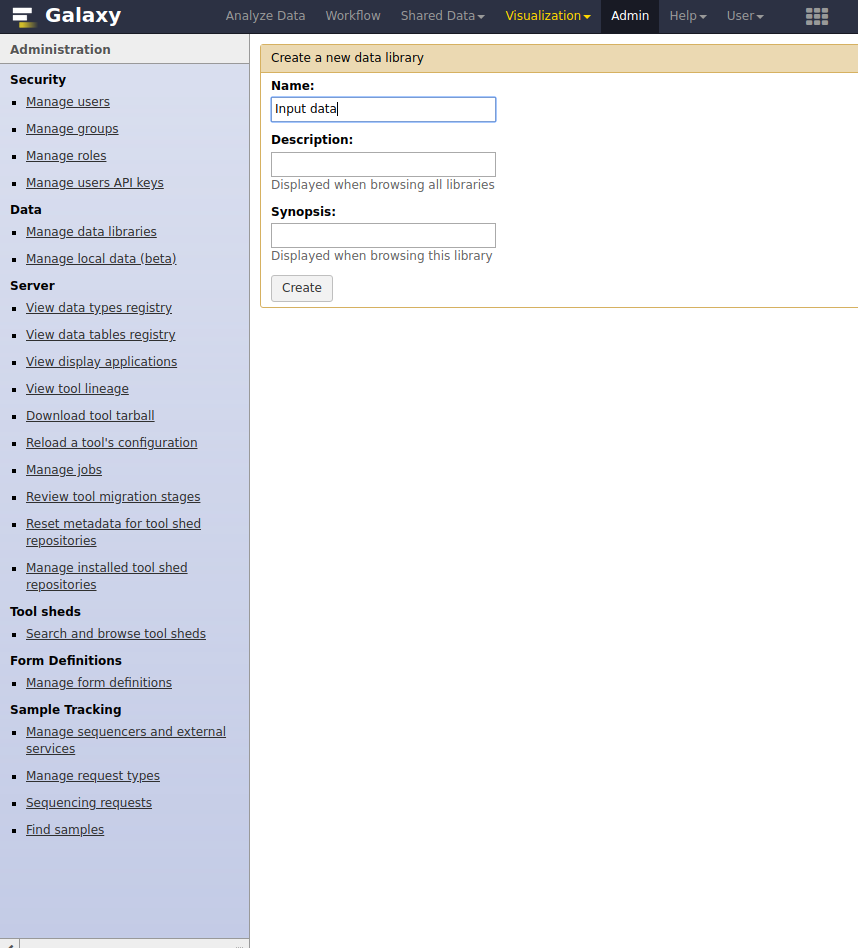


The screen will change and users will be in the screen for the new library, where they can “Add datasets”:


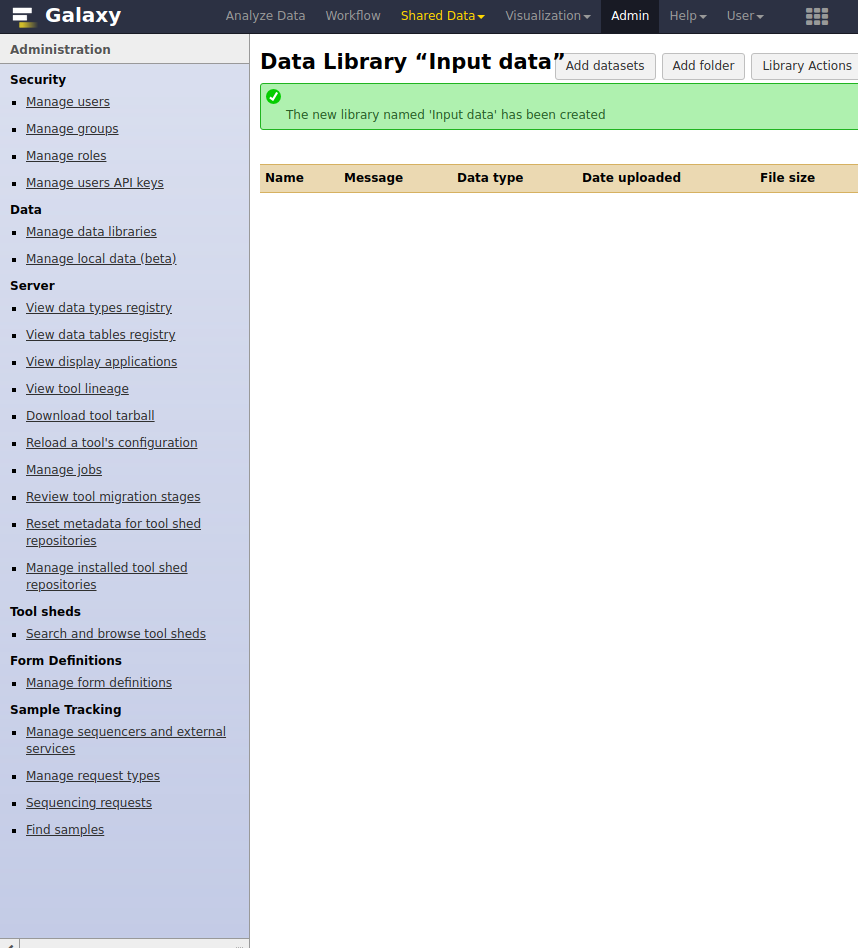


The “Add datasets” will open a new screen with multiple options, where it is key the the users select the option “Upload a directory of files” and then “Link to files without copying to Galaxy” from the drop down menu. This is key in order to have speed in importing the data, and avoid duplication within the Docker container given that the NGS read files are multiple GB in size. Finally, the users click the “Upload to Library” button at the bottom page.


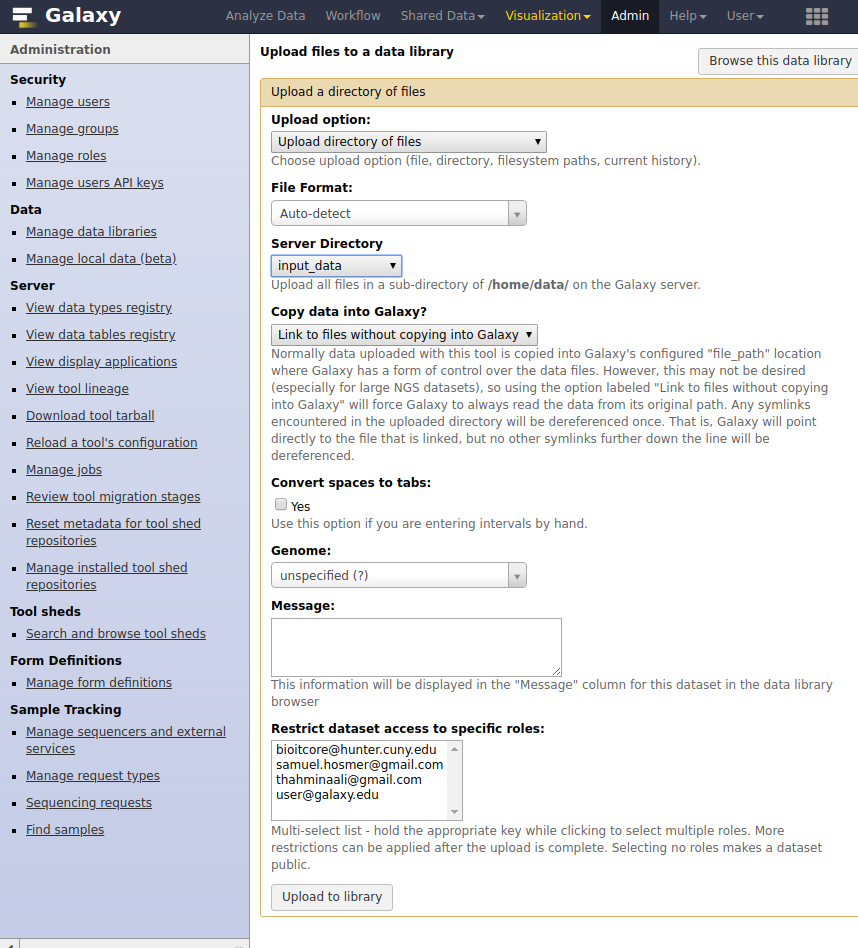


Then the page will change, and while the data are being linked and imported to Galaxy the “job is queued” progress bar will be displayed.


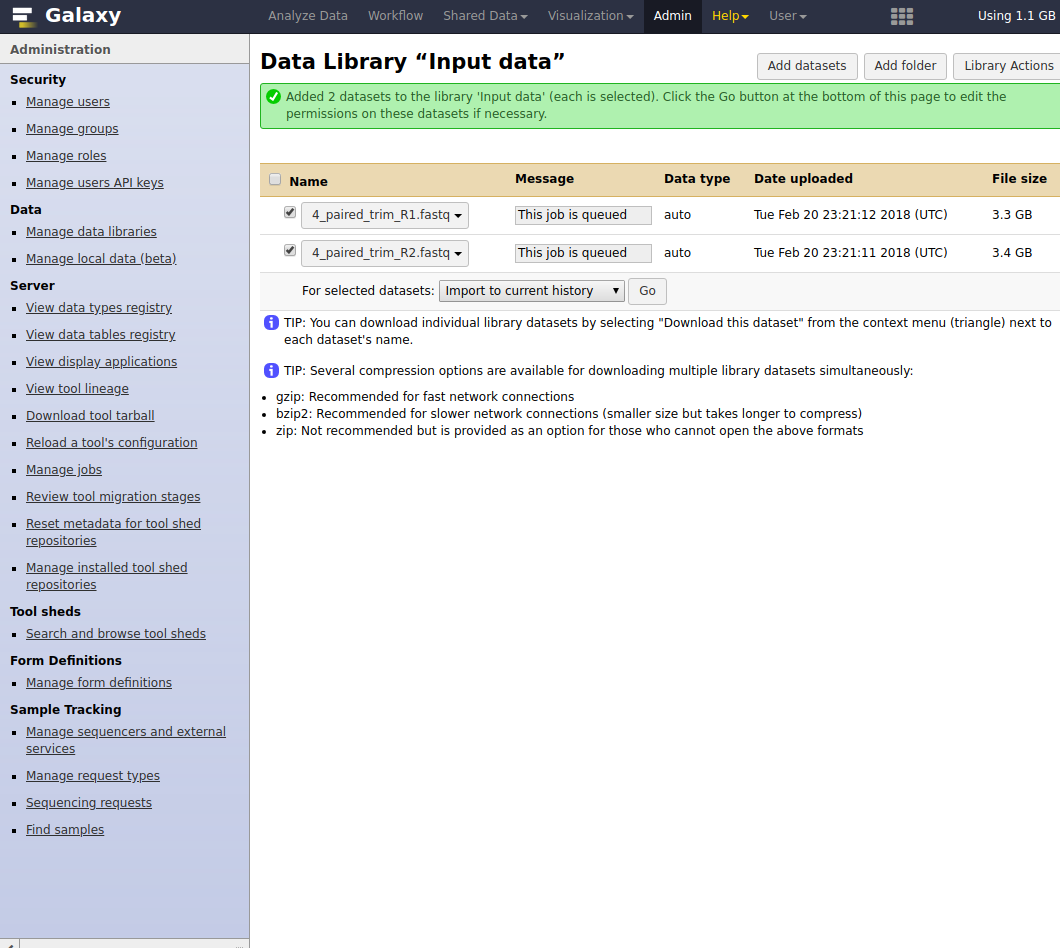


Once this has been been completed, users can click “Go” next to the “Import to current history”, and then the data are available and users can follow the same steps for running the MGS-Fast workflow as described earlier in this document.


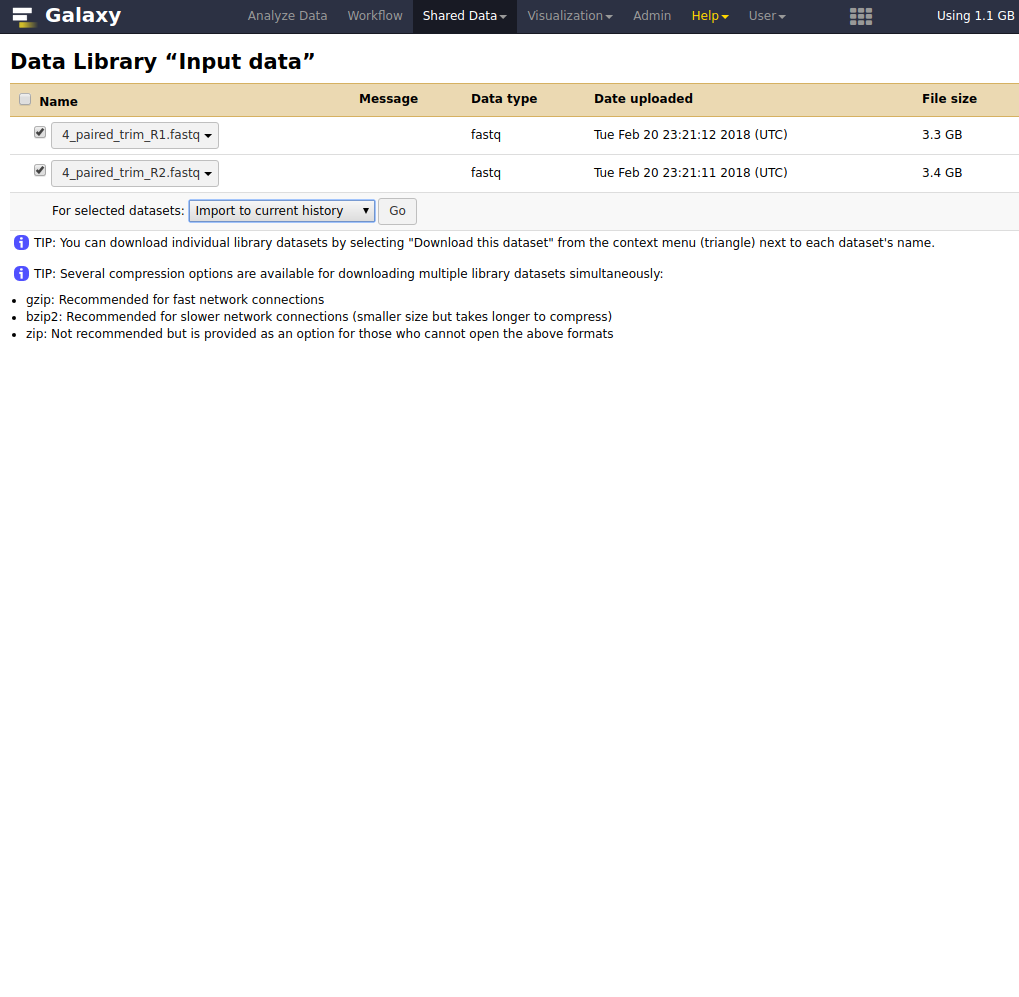


# Running MGS-Fast with multiple datasets in parallel.

If the users have a computational setup with adequate capacity, they can run multiple instances of the MGS-Fast pipeline in parallel for large-scale data analysis, by using data collections constructs in Galaxy (https://galaxyproject.org/tutorials/collections/), explained in detail in the following pictures. After selecting the MGS-Fast pipeline from the left Galaxy panel column as described previously, user can select “Collections” in the input (top tool in the list of tools of MGS-Fast displayed in the center of the Galaxy interface). The the screen will update as shown below, and users can select the first set (“_1”) of the reads in the pair by clicking the multiple files
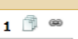
 button, and then holding down the control keyboard button and highlighting the files with the mouse, and similarly the files for the corresponding files in the read pair (“_2”).

#
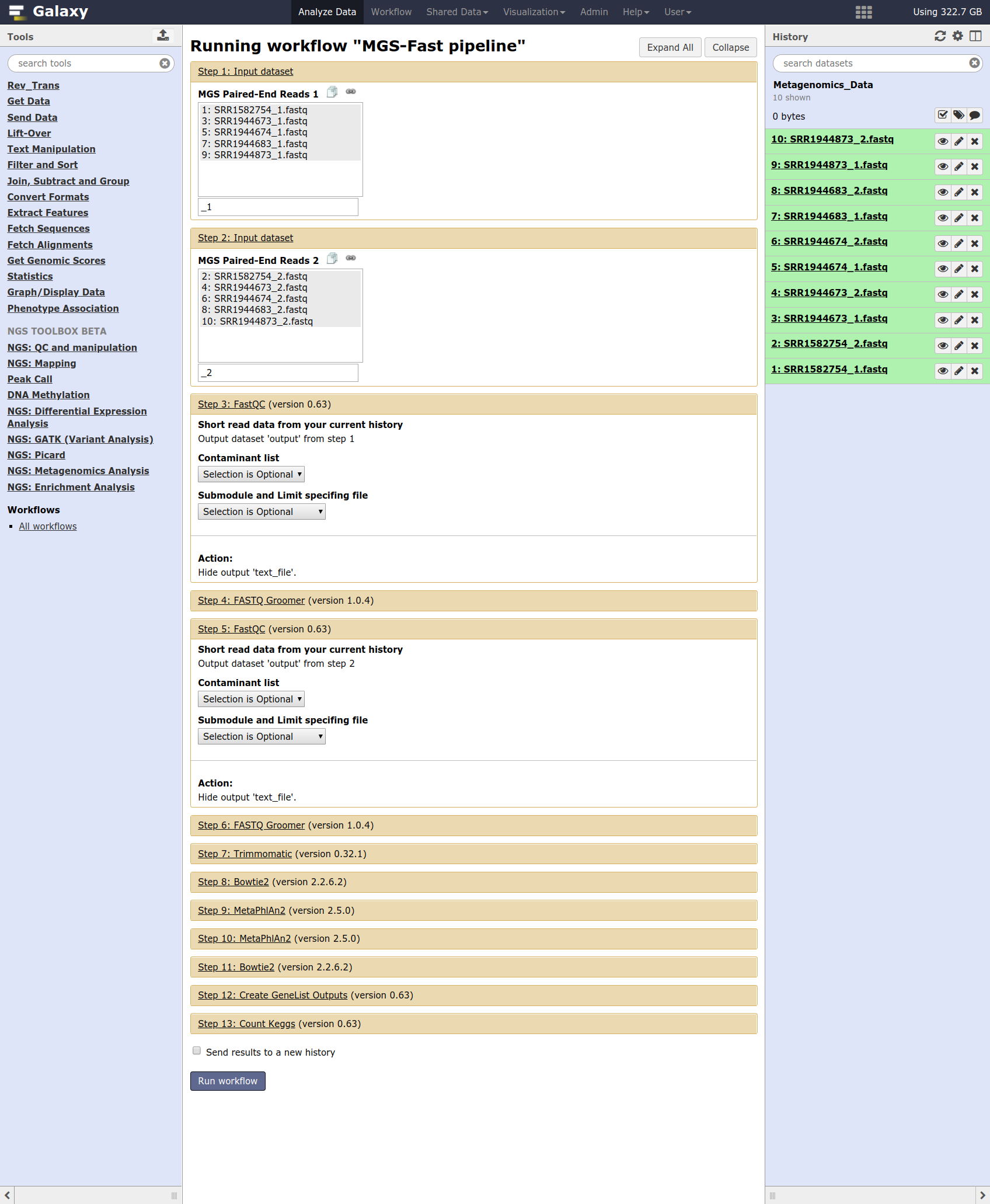


# Details of tools and parameters used in the MGS-Fast workflow.

The version of the tools in the workflow are the following: FASTQC 0.11.6 , Trimmomatic (0.32.1), Bowtie 2.2.6, MetaPhlAn 2.5.0. In addition the parameters used for each tool are shown below and these information have been also added in the Supplementary Material document.

*The Trimmomatic parameters used are the following:*

| *Perform initial ILLUMINACLIP step?* | *no* |  |
| --- | --- | --- |
| *Select Trimmomatic operation to perform* | *SLIDINGWINDOW* |  |
| *Number of bases to average across* | *4* |  |
| *Average quality required* | *20* |  |

*The Bowtie parameters for the filtering step with the human genome used are the following:*

| Set the minimum fragment length for valid paired-end alignments | 0 |  |
| --- | --- | --- |
| Set the maximum fragment length for valid paired-end alignments | 1000 |  |
| Select the upstream/downstream mate orientations for a valid paired-end alignment against the forward reference strand | --fr |  |
| Disable no-mixed behavior | False |  |
| Disable no-discordant behavior | True |  |
| Allow mate dovetailing | False |  |
| Disallow one mate alignment to contain another | False |  |
| Disallow mate alignments to overlap | False |  |
| Set read groups information? | do_not_set |  |
| Select analysis mode | simple |  |

# Troubleshooting

| **Issue** | **Error Message** | **Solution** |
| --- | --- | --- |
| If a user is already login to the Docker Hub, he/she may encounter errors while trying to run the docker login command again | docker login  Warning: failed to get default registry endpoint from daemon (Cannot connect to the Docker daemon at unix:///var/run/docker.sock. Is the docker daemon running?). Using system default: https://index.docker.io/v1/  Login with your Docker ID to push and pull images from Docker Hub. If you don't have a Docker ID, head over to https://hub.docker.com to create one.  Username (carloslijeron): carloslijeron  Password:  Cannot connect to the Docker daemon at unix:///var/run/docker.sock. Is the docker daemon running? | It is possible that you may be logged in already. Try moving on to the next step and if you can run your image, you have sucessfully logged into Docker. |

# Appendix

## About Docker

Docker is a software engine used for deploying applications in a virtual environment that allows portability and reproducibility of the application to be used on different machines and operating systems.

Some common docker commands are as follows:

| **Docker Command Line Options** | **Function** |
| --- | --- |
| -v | To mount the volume data |
| docker pull | Pull the container |
| docker run | Run the container |
| -i | Interactive |
| -t | Allocate a pseudo-TTY |
| -p | Publish a container's port to the host |

*For more docker command options:

<https://docs.docker.com/engine/reference/commandline/create/>

For additional information and documentation about Docker, please visit:

<https://docs.docker.com/>

##

##

## About Galaxy

Galaxy is an open source, web-based platform for data intensive biomedical research. The public instance of the Galaxy platform is provided on:

<https://usegalaxy.org/>

Using Docker, we have provided users with a private instance of the Galaxy platform in our container.

1. Note that pre-computed Bowtie2 indexes are much larger (and therefore slower to download) than the raw FASTA file, but require no additional indexing once downloaded. FASTA files must be indexed by the *bowtie-build* program, which can take substantial computing time. For further information about indices, take a look at [About the Metagenomics Workflow in Galaxy](#_3whwml4) [↑](#footnote-ref-0)
2. The Docker for Windows App only supports Windows 10 Professional Edition or Educational Edition. The Docker Toolbox supports older Windows versions, such as 7 or 8. For more information on Docker, take a look at [About Docker](#_49x2ik5) [↑](#footnote-ref-1)
3. You can get further information about Docker in the Appendix of this manual in the [About Docker](#_49x2ik5) section [↑](#footnote-ref-2)
4. You can get further information about Galaxy in the Appendix of this manual in the [About Galaxy](#_1pxezwc) section [↑](#footnote-ref-3)
5. Take a look at the setup of our Workflow in the [About the Metagenomics Workflow in Galaxy](#_3whwml4) section later in the manual [↑](#footnote-ref-4)
6. You can get further information about Galaxy in the Appendix of this manual in the [About Galaxy](#_1pxezwc) section. [↑](#footnote-ref-5)
