# Supplementary material for: MGS-Fast: Metagenomic shotgun data fast annotation using microbial gene catalogs
Source: Gigascience. 2019 Apr 3;8(4):giz020. doi: 10.1093/gigascience/giz020 (PMC6446249; doi:10.1093/gigascience/giz020)
Supplement: Response-to-Reviewer-Comments_Original-Submission.pdf [file giz020_response-to-reviewer-comments_original-submission.pdf]

# Author's Response To Reviewer Comments

Close

ATTACHED RESPONSE TO REVIEWERS AS SUPPLEMENTARY .doc FOR CLEARER FORMATTING.

Close
