# Supplementary material for: MGS-Fast: Metagenomic shotgun data fast annotation using microbial gene catalogs
Source: Gigascience. 2019 Apr 3;8(4):giz020. doi: 10.1093/gigascience/giz020 (PMC6446249; doi:10.1093/gigascience/giz020)
Supplement: Reviewer-2-Report-Original-Submission -- Nicola Segata — 8/10/2018 Reviewed [file giz020_reviewer-2-report-original-submission.pdf]

Reviewer Report

Title: MGS-Fast: MetaGenomic Shotgun data Fast annotation using microbial gene catalogs

Version: Original Submission **Date:** 8/10/2018

Reviewer name: Nicola Segata

Reviewer Comments to Author:

The authors present a work on a tool for metagenomic data analysis. Because this is a re-submission of a previous version of the work, I was expecting a point-by-point response to the issues raised by the reviewers, but I didn't find such document in the submission material.
The main strength of the work is that the authors provide a pipeline embedded in Galaxy and Docker that can be used by other users. However, the main issue I see with the work is that the methodology is not novel. The core of the method is the mapping of the metagenomic reads against a custom functional database using BowTie2 which is a pretty standard approach for metagenomic analysis. Moreover, there are now software like Diamond (and others) that are able to perform translated search (reads to proteins) very efficiently and faster than BowTie2.
Other major issues:
- The authors applied the method on 20 samples from a larger study with hundreds of samples. Current metagenomic studies generally have hundreds of samples. To be convincing the authors need to apply their pipeline to full datasets and compare their results with those obtained by the original work.
- For a fair comparison on the performances, the authors should have used the same database for all the used methods. It is known that Kraken is faster than BowTie2, so I would expect the proposed pipeline to be slower when using the same database.
- The validation of the method is not convincing, as basically only the mapping rate is reported. A validation with mock communities on the task of functional profiling is needed to convince a potential user.
Other comments
- Line 57. It i not true that "BLAST [4], is the most commonly used" approach to map reads against references. I would say that BLAST is not used at all for metagenomic reads.
- The second paragraph of the "Background" is a bit confusing. There are advantages in using assembly to profile genes, and the real advantages of the mapping approach are not clearly state. One strong point should also be that by mapping it is possible to recover the presence of a gene even when the coverage for that gene is not sufficient for assembling it.

Level of Interest

Please indicate how interesting you found the manuscript: Choose an item.

Quality of Written English

Please indicate the quality of language in the manuscript: Choose an item.

Declaration of Competing Interests

Please complete a declaration of competing interests, considering the following questions:

- Have you in the past five years received reimbursements, fees, funding, or salary from an organisation that may in any way gain or lose financially from the publication of this manuscript, either now or in the future?
- Do you hold any stocks or shares in an organisation that may in any way gain or lose financially from the publication of this manuscript, either now or in the future?
- Do you hold or are you currently applying for any patents relating to the content of the manuscript?
- Have you received reimbursements, fees, funding, or salary from an organization that holds or has applied for patents relating to the content of the manuscript?
- Do you have any other financial competing interests?
- Do you have any non-financial competing interests in relation to this paper?

If you can answer no to all of the above, write 'I declare that I have no competing interests' below. If your reply is yes to any, please give details below.

I declare that I have no competing interests

I agree to the open peer review policy of the journal. I understand that my name will be included on my report to the authors and, if the manuscript is accepted for publication, my named report including any attachments I upload will be posted on the website along with the authors' responses. I agree for my report to be made available under an Open Access Creative Commons CC-BY license (http://creativecommons.org/licenses/by/4.0/). I understand that any comments which I do not wish to be included in my named report can be included as confidential comments to the editors, which will not be published.

I agree to the open peer review policy of the journal

To further support our reviewers, we have joined with Publons, where you can gain additional credit to further highlight your hard work (see: https://publons.com/journal/530/gigascience). On publication of this paper, your review will be automatically added to Publons, you can then choose whether or not to claim your Publons credit. I understand this statement.

Yes Choose an item.
